# Defect and structural evolution under high-energy ion irradiation informs battery materials design for extreme environments

**DOI:** 10.1038/s41467-020-18345-4

**Published:** 2020-09-11

**Authors:** Muhammad Mominur Rahman, Wei-Ying Chen, Linqin Mu, Zhengrui Xu, Ziqi Xiao, Meimei Li, Xian-Ming Bai, Feng Lin

**Affiliations:** 1 Department of Chemistry, Virginia Tech, Blacksburg, VA 24061 USA; 2 Argonne National Laboratory, Nuclear Science and Engineering Division, Lemont, IL 60439 USA; 3 Department of Materials Science and Engineering, Virginia Tech, Blacksburg, VA 24061 USA

**Keywords:** Energy science and technology, Materials science

## Abstract

Understanding defect evolution and structural transformations constitutes a prominent research frontier for ultimately controlling the electrochemical properties of advanced battery materials. Herein, for the first time, we utilize in situ high-energy Kr ion irradiation with transmission electron microscopy to monitor how defects and microstructures evolve in Na- and Li-layered cathodes with 3d transition metals. Our experimental and theoretical analyses reveal that Li-layered cathodes are more resistant to radiation-induced structural transformations, such as amorphization than Na-layered cathodes. The underlying mechanism is the facile formation of Li-transition metal antisite defects in Li-layered cathodes. The quantitative mathematical analysis of the dynamic bright-field imaging shows that defect clusters preferentially align along the Na/Li ion diffusion channels (*a-b* planes), which is likely governed by the formation of dislocation loops. Our study provides critical insights into designing battery materials for extreme irradiation environments and understanding fundamental defect dynamics in layered oxides.

## Abstract (web summary)

Defect and structural evolution are critical in determining the stability of battery materials. Here, the authors use high-energy Kr ion irradiation to induce rapid defect and study structural evolution in Li- and Na-layered cathodes to find that Li-layered cathodes are more resilient under irradiation.

## Introduction

Crystal defects play a critical role in influencing the physicochemical properties of metal oxides^1,2^, such as catalytic activity^3^, optical absorption^4^, electronic^5^, and electromagnetic properties^6^. Thus, defect engineering has gained broad attention as a method of tailoring metal oxide characteristics^7^. Layered transition metal oxides are extensively utilized as cathodes for the state-of-the-art rechargeable batteries^8,9^. Defects in these materials can be induced by the high-temperature synthesis^10^ and electrochemical cycling^11^ and can broadly influence battery properties. For example, defect dynamics is related to capacity loss^12^, ion migration^13,14^, voltage hysteresis^15^, and structural transformations during cycling^16^. Voltage fade and oxygen loss in Li-rich layered cathodes are directly correlated to the defect evolution^17^. However, it has also been reported that defects can relieve the strain by acting as an interface between two phases during phase transformation^18^. Defect engineering can enhance electrochemical performance in certain cases^19,20^. Thus, a recent incentive is to control and monitor the defect evolution to enhance the electrochemical performance of battery electrodes. However, efficient monitoring of defect dynamics is still a challenging task. Researchers have been developing techniques that can track defects under operating conditions^21,22^. Ulvestad et al.^23^ and Singer et al.^17^ utilized Bragg coherent diffraction imaging to monitor dislocation dynamics in spinel LiNi_0.5_Mn_1.5_O_4_ and layered Li_1.2_Ni_0.333_Mn_0.333_Co_0.333_O_2_ cathodes, respectively. However, a limited resolution of the technique means that point defects or small defect clusters are difficult to characterize^24^. Transmission electron microscopy (TEM) with high spatial/temporal resolution may provide a solution in this regard^22^.

Defect and structural evolution can be accelerated in complex oxides through high-energy ion irradiation^25,26^. Ion irradiation in conjunction with TEM has been utilized to understand the irradiation damage in nuclear reactor materials and fuels^27–30^. Alkali-ion batteries have the potential to be utilized in extreme environments, such as outer space and nuclear power industries, where high-energy irradiation can impart significant damage to materials^31,32^. Accelerated degradation of cell components, such as cathode and electrolyte, has been observed under neutron and gamma irradiation^31,33^. Radiation-induced hardness is observed in perovskite tandem solar cells^34^. Structural transformation, for example, amorphization can take place in a crystalline material under extreme irradiation^35^. For the reliable performance of battery materials in extreme environments, these materials are required to be resistant to such structural damage. Under irradiation, high-energy particles, such as neutron or Kr ions, can displace atoms away from their lattice sites and form a locally disordered region, called cascade^36–38^. A cascade can recover in a few picoseconds (10^−12^ s), but some displaced atoms can form defects, such as interstitials and vacancies. The aggregation of these point defects can form extended defects, such as dislocation loops and voids^39^. Dislocation loop and void formation will require the diffusion of interstitials and vacancies at the temperature of irradiation, respectively. In comparison, interstitial-type defects are also formed during electrochemical cycling through transition metal migration in the interlayer space^40,41^. Such migration can lead to structural transformation^42^ and voltage fading^43,44^. Vacancy cluster formation in Na_0.75_Li_0.25_Mn_0.75_O_2_ is reported in as early as the first cycle^15^. Since vacancies and interstitials are also formed under ion irradiation, the material damage due to ion irradiation shares some similarities with the electrochemical cycling. Furthermore, the ability to create high-density defects in a short time through ion irradiation enables studying defect and structural evolution in situ^45^, thus overcoming the limitation of slow defect evolution through electrochemical cycling.

In this study, we explore the defect and structural evolution in layered cathodes with 3*d* transition metals (A_*x*_TMO_2_, where A is akali ion, TM is transition metal ion, and *x* is ≤1) under high-energy Kr ion irradiation. Kr ion irradiation can induce observable damage within a short period of time^46^. The cascade damage profile produced by Kr ion irradiation is similar to neutron irradiation in a nuclear reactor. Hence, efficient mirroring of the defect and structural evolution throughout the actual service life in extreme environments is possible within the timescale of a laboratory experiment. Layered P2-Na_2/3_Fe_1/2_Mn_1/2_O_2_ (space group: *P*6_3_/*mmc*) and O3-LiNiO_2_ (space group: *R*$\bar 3$*m*) are utilized as the model materials for this study. P2-Na_2/3_Fe_1/2_Mn_1/2_O_2_ has received broad attention because it contains only earth-abundant elements and delivers high discharge capacity^47^. LiNiO_2_ has been revitalized recently because of the incentive to eliminate high cost and child labor-intensive Co from cathodes^48^. In situ TEM imaging, electron diffraction, and density functional theory (DFT)-based calculations have revealed that Li-layered oxides are more resistant to irradiation-induced structural transformation (e.g., amorphization) than Na-layered oxides. Our comprehensive mathematical analysis on the bright-field two-beam images of the irradiated materials shows that defect clusters tend to aggregate preferentially along the *a*–*b* planes of the irradiated materials. Electrochemically cycled cathodes also exhibit similar behavior as exemplified by the similarity between irradiated LiNiO_2_ and delithiated LiNiO_2_.

## Results

### Physical and electrochemical characterization of layered cathodes

**Fig. 1:**
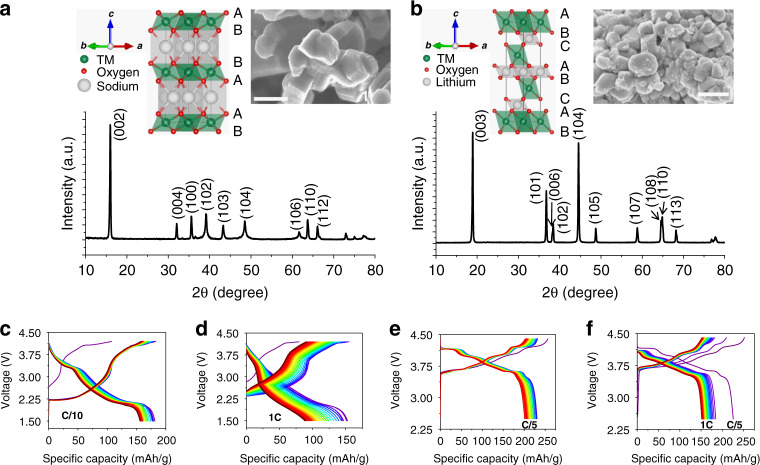
Physical and electrochemical characterization of pristine materials. **a** XRD pattern of Na_2/3_Fe_1/2_Mn_1/2_O_2_. The inset shows the crystal structure and SEM image of the material. The scale bar in the SEM image corresponds to a length of 500 nm. **b** XRD pattern of LiNiO_2_. The inset shows the crystal structure and SEM image of the material. The scale bar in the SEM image corresponds to a length of 500 nm. **c** Charge and discharge curves of Na half cell containing the Na_2/3_Fe_1/2_Mn_1/2_O_2_ as the cathode material at a rate of C/10. **d** Charge and discharge curves of Na half cell containing the Na_2/3_Fe_1/2_Mn_1/2_O_2_ as the cathode material at a rate of 1C. **e** Charge and discharge curves of Li half cell containing the LiNiO_2_ as the cathode material at a rate of C/5. **f** Charge and discharge curves of Li half cell containing the LiNiO_2_ as the cathode material at a rate of 1C. The first cycle is at C/5 rate. The charge and discharge curves for both materials are plotted for up to 20 cycles.

Na_2/3_Fe_1/2_Mn_1/2_O_2_ crystallizes into a layered structure with ABBA-type oxygen stacking (P2 type) and the Na ion in the interlayer space is in prismatic coordination with the oxygen ions (inset of Fig. 1a). All diffraction peaks in the X-ray powder diffraction (XRD) pattern can be indexed towards a pure hexagonal lattice with a *P*6_3_/*mmc* space group (Fig. 1a), isostructural to P2-Na_*x*_CoO_2_^49^. LiNiO_2_ crystallizes into a layered structure with ABCABC-type oxygen stacking (O3 type). Li ion is in octahedral coordination with oxygen ions (inset of Fig. 1b). The diffraction peaks in the XRD pattern can be indexed towards a pure rhombohedral lattice with *R*$\bar 3$*m* space group^50^, isostructural to α-NaFeO_2_ (Fig. 1b). The primary particles of both materials have random morphology (inset of Fig. 1a, b). Na_2/3_Fe_1/2_Mn_1/2_O_2_ delivers a specific discharge capacity of 185–190 mAh/g at C/10 rate (Fig. 1c) and 150–155 mAh/g at 1C rate (Fig. 1d) in Na half cells. LiNiO_2_ delivers 225 mAh/g capacity at C/5 rate (Fig. 1e) and 185 mAh/g at 1C rate (Fig. 1f) in Li half cells. The capacity and capacity retention (Supplementary Fig. 1) delivered by these materials are comparable to those reported in the literature^47,48^. In summary, the phase pure crystal structure along with the electrochemical performance shows that these materials are representative and can provide a good platform for studying the defect and structural evolution of Li- and Na-layered cathodes under extreme environments.

### Structural transformation under in situ Kr ion irradiation

Kr ion with an energy of 1 MeV at room temperature is used to irradiate Na_2/3_Fe_1/2_Mn_1/2_O_2_ and LiNiO_2_ to induce defects and structural transformations. SRIM (stopping and range of ions in the matter) simulation^51^ is performed to understand the Kr ion concentrations and damage profiles within the materials (Supplementary Fig. 2). The simulation shows that for a particle with a 1000 nm thickness, the maximum Kr ion concentration is at a depth of ~400 nm for both Na_2/3_Fe_1/2_Mn_1/2_O_2_ and LiNiO_2_ (Supplementary Fig. 2a, c). The maximum number of vacancies (peak damage) is produced within the depth of ~300 nm of both Na_2/3_Fe_1/2_Mn_1/2_O_2_ and LiNiO_2_ (Supplementary Fig. 2b, d).

**Fig. 2:**
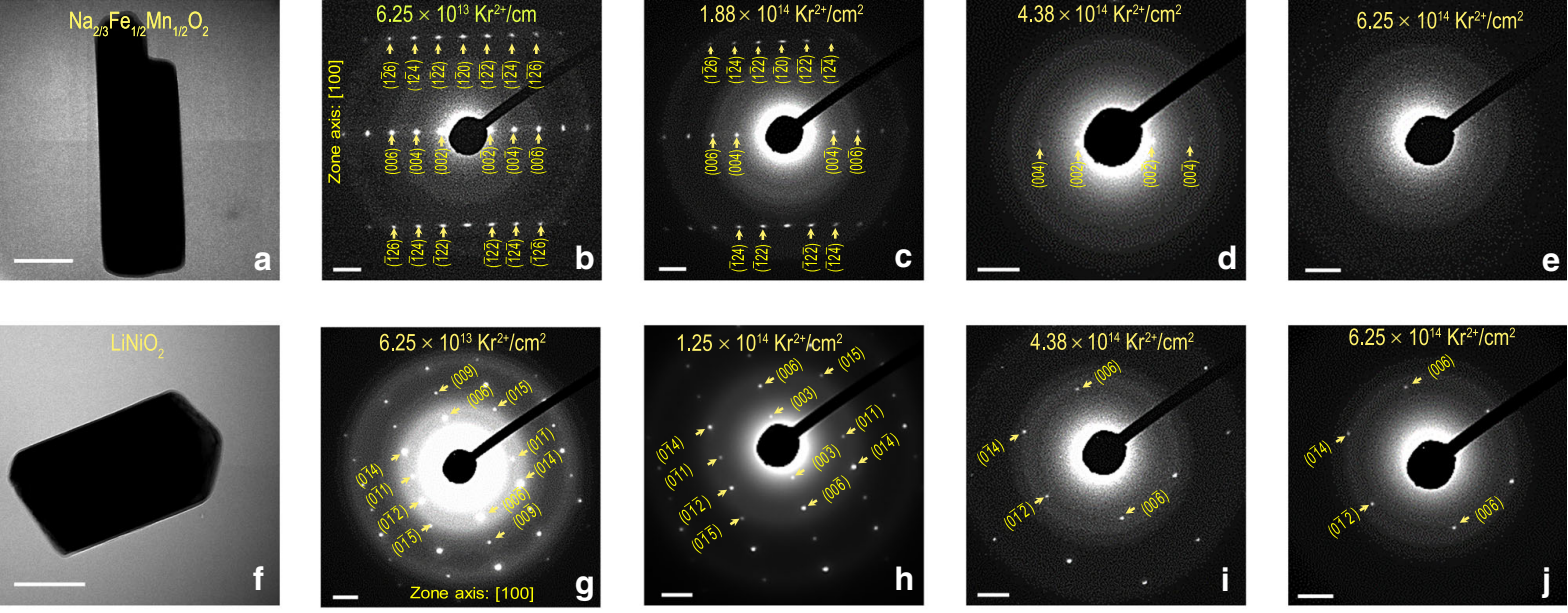
In situ structural evolution of layered cathodes under Kr ion irradiation. **a** The Na_2/3_Fe_1/2_Mn_1/2_O_2_ particle that is irradiated at room temperature. The scale bar corresponds to a length of 100 nm. Electron diffraction of Na_2/3_Fe_1/2_Mn_1/2_O_2_ at the fluence of **b** 6.25 × 10^13^ Kr^2+^/cm^2^, **c** 1.88 × 10^14^ Kr^2+^/cm^2^, **d** 4.38 × 10^14^ Kr^2+^/cm^2^, and **e** 6.25 × 10^14^ Kr^2+^/cm^2^. The scale bars in **b**–**e** are equivalent to 2 1/nm. **f** The LiNiO_2_ particle that is irradiated at room temperature. The scale bar corresponds to a length of 100 nm. Electron diffraction of LiNiO_2_ at the fluence of **g** 6.25 × 10^13^ Kr^2+^/cm^2^, **h** 1.25 × 10^14^ Kr^2+^/cm^2^, **i** 4.38 × 10^14^ Kr^2+^/cm^2^, and **j** 6.25 × 10^14^ Kr^2+^/cm^2^. The scale bars in **g**–**j** are equivalent to 2 1/nm.

Structural evolution is monitored in situ by electron diffraction (ED) with increasing fluence of Kr ion irradiation at room temperature (Fig. 2). The ED of Na_2/3_Fe_1/2_Mn_1/2_O_2_ can be indexed as lattice planes from a hexagonal lattice with *P*6_3_/*mmc* space group when viewed from the [100] zone axis (Fig. 2b), in corroboration with the global crystal structure deciphered from the XRD pattern. The diffraction spots from the particle of LiNiO_2_ can be indexed as lattice planes from the rhombohedral lattice with the space group *R*$\bar 3$*m* when viewed from the [100] zone axis (Fig. 2g), in corroboration with the global XRD pattern of the material. The brightness of the diffraction spots can be a measure of the crystallinity of the materials. For irradiated Na_2/3_Fe_1/2_Mn_1/2_O_2_ and LiNiO_2_, the spots get dimmer with increasing fluence of Kr ion irradiation. We measured the brightness of the spots in terms of pixel values of a grayscale image (black being 0 and white being 255 in pixel value). Starting with a range of pixel values from 200 to 255 within the spot, the spots for the lattice plane (004) of Na_2/3_Fe_1/2_Mn_1/2_O_2_ and (006) plane of LiNiO_2_ contain increasingly less number of pixels within the same range (Supplementary Figs. 3 and  4), indicating that the materials are losing crystallinity with increasing fluence of Kr ion irradiation. However, a striking dissimilarity is observed when we compare the resistance to loss of crystallinity between Na_2/3_Fe_1/2_Mn_1/2_O_2_ and LiNiO_2_. At a fluence of 4.38 × 10^14^ Kr^2+^/cm^2^, many of the diffraction spots of Na_2/3_Fe_1/2_Mn_1/2_O_2_ disappear and only those from the (00*l*) lattice planes remain (Fig. 2d). At 6.25 × 10^14^ Kr^2+^/cm^2^, the particle of Na_2/3_Fe_1/2_Mn_1/2_O_2_ becomes completely amorphous since all the spots from the lattice planes disappear (Fig. 2e). However, the particle of LiNiO_2_ at that particular fluence still maintains some of its crystallinity since some of the spots from both (0*kl*) and (00*l*) are observable (Fig. 2j). In fact, even at 1.25 × 10^15^ Kr^2+^/cm^2^, that is, double the fluence of 6.25 × 10^14^ Kr^2+^/cm^2^, LiNiO_2_ still maintains some crystallinity (Supplementary Fig. 5b). Hence, LiNiO_2_ is more resistant to amorphization than Na_2/3_Fe_1/2_Mn_1/2_O_2_ when irradiated with high-energy ion beam to the same fluence. Similar to pristine LiNiO_2_, electrochemically delithiated LiNiO_2_ (charged to 4.5 V against Li^+^/Li) is also more resistant to radiation-induced structural damage than Na_2/3_Fe_1/2_Mn_1/2_O_2_ (Supplementary Fig. 6). It should be noted that, in some cases, the electron beam utilized for imaging has been reported to induce structural transformations in a material^52,53^. However, upon prolonged exposure to electron beam irradiation alone (up to 1 h), no significant microstructural evolution in Na_2/3_Fe_1/2_Mn_1/2_O_2_ is observed in this work (Supplementary Fig. 7a–f). In comparison, the structural damage induced by Kr ion irradiation is significantly larger and accounts for the most structural changes observed in the material (Fig. 2 and Supplementary Fig. 7g–l).

**Fig. 3:**
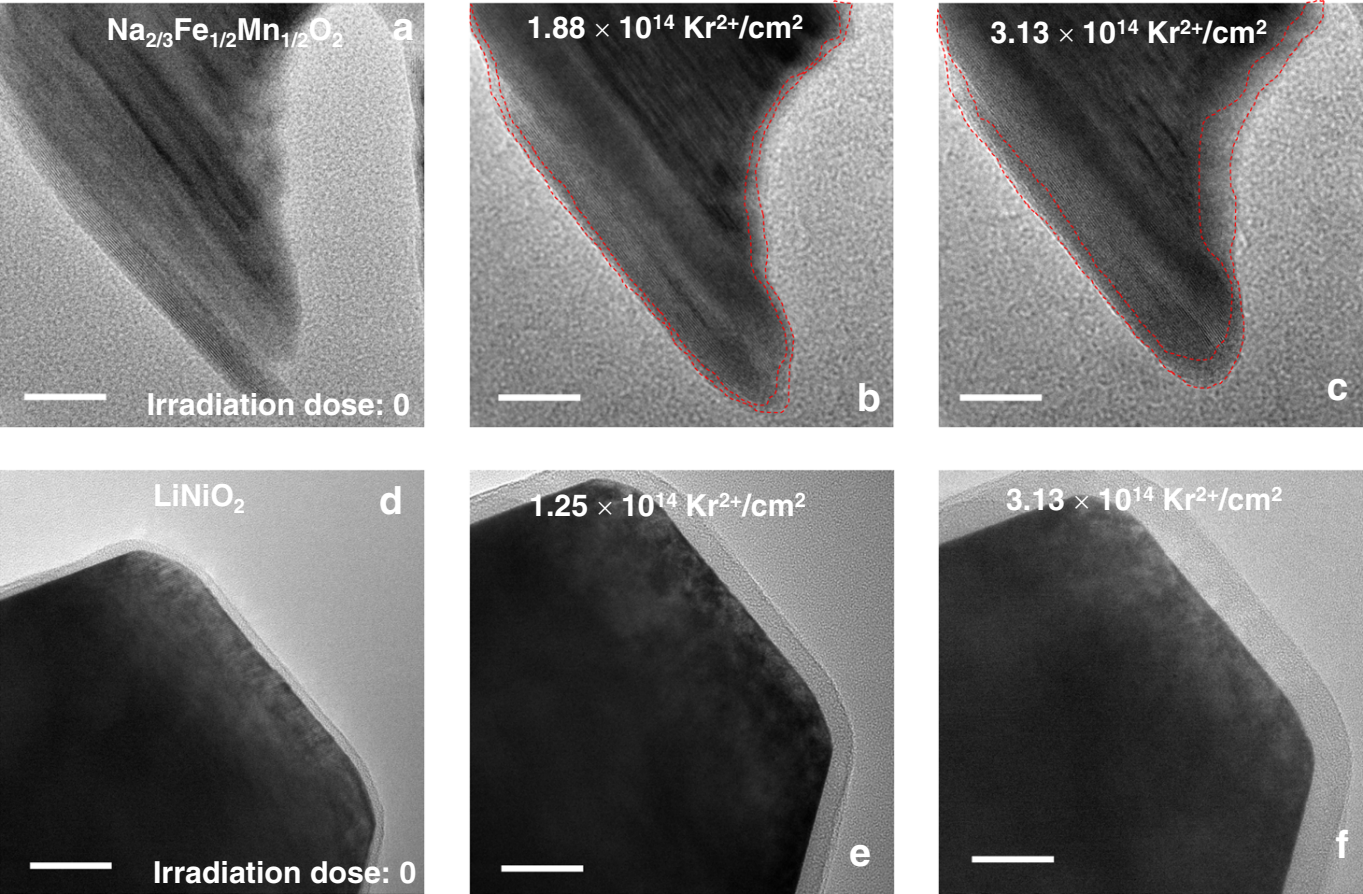
TEM images of Na_2/3_Fe_1/2_Mn_1/2_O_2_ and LiNiO_2_ under Kr ion irradiation. **a** TEM image of Na_2/3_Fe_1/2_Mn_1/2_O_2_ before irradiation. TEM images of Na_2/3_Fe_1/2_Mn_1/2_O_2_ at the fluence of **b** 1.88 × 10^14^ Kr^2+^/cm^2^, and **c** 3.13 × 10^14^ Kr^2+^/cm^2^ at room temperature. The red dashed lines in (**b**–**c**) indicate the growth of the amorphous layer upon irradiation. **d** TEM image of LiNiO_2_ before irradiation. TEM images of LiNiO_2_ at the fluence of **e** 1.25 × 10^14^ Kr^2+^/cm^2^, and **f** 3.13 × 10^14^ Kr^2+^/cm^2^ at room temperature. All the scale bars correspond to a length of 20 nm.

Loss of crystallinity in Na_2/3_Fe_1/2_Mn_1/2_O_2_ under Kr ion irradiation (Fig. 2) is accompanied by the formation of amorphous regions on the particles (Fig. 3). Figure 3a–c and Supplementary Fig. 8 show the microstructural evolution of Na_2/3_Fe_1/2_Mn_1/2_O_2_ particle. The area of the amorphous region increases with irradiation (Fig. 3a–c, and Supplementary Fig. 9) until the particle becomes fully amorphous at the fluence of 6.25 × 10^14^ Kr^2+^/cm^2^ (Supplementary Fig. 10g), in corroboration with Fig. 2e. The area of the amorphous layer on the surface of LiNiO_2_ particle also seems to increase with irradiation (Fig. 3d–f and Supplementary Fig. 11), although complete amorphization is not observed. It must be noted that the amorphous layers on these two materials are fundamentally distinct from each other. The growth of the amorphous layer within the particle of Na_2/3_Fe_1/2_Mn_1/2_O_2_ indicates a transformation from the crystalline to the amorphous phase, which is supported by the ED results (Fig. 2a–e). Meanwhile, the transparent amorphous layer on the surface of LiNiO_2_ indicates that the growth of this layer is due to the entrapment of trace carbon by electrons inside the TEM column^54,55^.

The observed differences in structural transformations between the Na- and Li-layered cathode can possibly be explained based on the previous studies of other metal oxides^29,30^. These studies indicated that in pyrochlores (A_2_B_2_O_7_, where A and B indicate two different cations), the formation energy of the cationic antisite defect pair is inversely related to the resistance to radiation tolerance. The formation energy would depend on the difference in ionic radius between two types of cations in pyrochlores. A large difference in ionic radius will have a high energy for cationic antisite defect formation and vice versa. The difference in ionic radius between Li^+^ and Ni^3+^ in LiNiO_2_ is smaller than the difference in ionic radius between Na^+^ and the transition metal ions (Fe^3+^ and Mn^4+^) in Na_2/3_Fe_1/2_Mn_1/2_O_2_, using the ionic radii provided by Shannon and Prewitt^56^. Hence, LiNiO_2_ should be more radiation-resistant than Na_2/3_Fe_1/2_Mn_1/2_O_2_ based on this argument from an earlier study^29^, which is consistent with our experimental observations. However, it is yet to be determined if antisite formation energy can be directly correlated with the radiation tolerance in layered oxide materials. A detailed account of the relationship between the cationic antisite defect formation energy and resistance to radiation damage of layered oxides is provided later by DFT calculations.

Structural transformations also depend on the temperature. At a high temperature (e.g., 200 °C), Na_2/3_Fe_1/2_Mn_1/2_O_2_ displays more resistance to amorphization than at room temperature (Supplementary Fig. 12). At 200 °C, Na_2/3_Fe_1/2_Mn_1/2_O_2_ still maintains some crystallinity when irradiated at the fluence of 6.25 × 10^14^ Kr^2+^/cm^2^ (Supplementary Fig. 12d), which is the dose required for amorphization at room temperature (see Fig. 2e). However, instead of going through a direct layered to amorphous transformation observed at room temperature, an intermediate spinel phase (space group: *Fd*$\bar 3m$) is observed at 200 °C (Supplementary Fig. 12b). The spots for the spinel phase start to form partial rings at higher fluence (Supplementary Fig. 12c–f), indicating the development of a polycrystalline nature of the emerging spinel phase. In fact, from the TEM image, a number of small domains of the spinel phase are observed at the fluence of 3.13 × 10^14^ Kr^2+^/cm^2^ (Supplementary Fig. 13c). Formation of the spinel phase may indicate oxygen evolution in order to form a cation densified state, according to previously reported literature^57^. Meanwhile at a low temperature (−173 °C), the resistance to amorphization of Na_2/3_Fe_1/2_Mn_1/2_O_2_ decreases significantly (Supplementary Fig. 14). The material becomes completely amorphous even at a fluence as low as 1.25 × 10^14^ Kr^2+^/cm^2^ (Supplementary Fig. 14c). Thus, it is evident that the critical dose for complete amorphization of layered materials strongly depends on temperature and increases with the elevation of temperature, which is similar to other ceramics^58,59^. This is because defect annihilation typically accelerates as temperature increases, thus increasing the critical dose of amorphization^60,61^.

### Dynamic defect evolution under in situ Kr ion irradiation

**Fig. 4:**
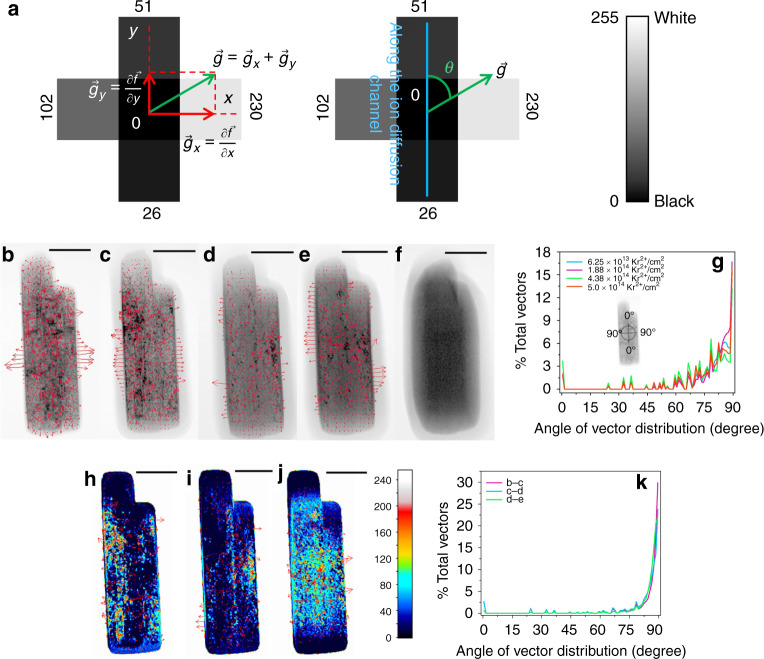
Defect clusters evolution in Na_2/3_Fe_1/2_Mn_1/2_O_2_ under Kr ion irradiation. Grayscale bright-field two-beam images are acquired to study the defect clusters distribution and evolution. **a** Scheme presenting the calculation of the gradient vector from a certain pixel of a bright-field two-beam image. The gradient vector points to the overall directional change in pixel value. Gradient vector calculated and superimposed on the bright-field two-beam image of a Na_2/3_Fe_1/2_Mn_1/2_O_2_ particle irradiated at the total fluence of **b** 6.25 × 10^13^ Kr^2+^/cm^2^, **c** 1.88 × 10^14^ Kr^2+^/cm^2^, **d** 4.38 × 10^14^ Kr^2+^/cm^2^, and **e** 5.0 × 10^14^ Kr^2+^/cm^2^ at room temperature. Bright-field two-beam image of a Na_2/3_Fe_1/2_Mn_1/2_O_2_ particle irradiated at the total fluence of **f** 6.25 × 10^14^ Kr^2+^/cm^2^ at room temperature. The bright-field images are taken from the [100] zone axis. All the scale bars in the image b–f correspond to a length of 100 nm. **g** Distribution of the gradient vectors of image b–e against the angle of the gradient vector. The inset shows the scheme of how the angle of the gradient vector is defined. Dynamic defect evolution in a Na_2/3_Fe_1/2_Mn_1/2_O_2_ particle with increasing fluence of Kr ion irradiation  (**h**–**j**). The dynamic defect evolution is studied through the subtraction of the image acquired at higher irradiation dose from that of the lower irradiation dose (e.g., image c subtracted from image b). Defect evolution from **h** image b–c, **i** image c–d, and **j** image d–e. All the scale bars from images h–j correspond to a length of 100 nm. The color bar shows the corresponding values of the subtracted pixels after the subtracted grayscale image is converted to an RGB image. **k** Distribution of the gradient vectors of images h–j against the angle of the gradient vector.

**Fig. 5:**
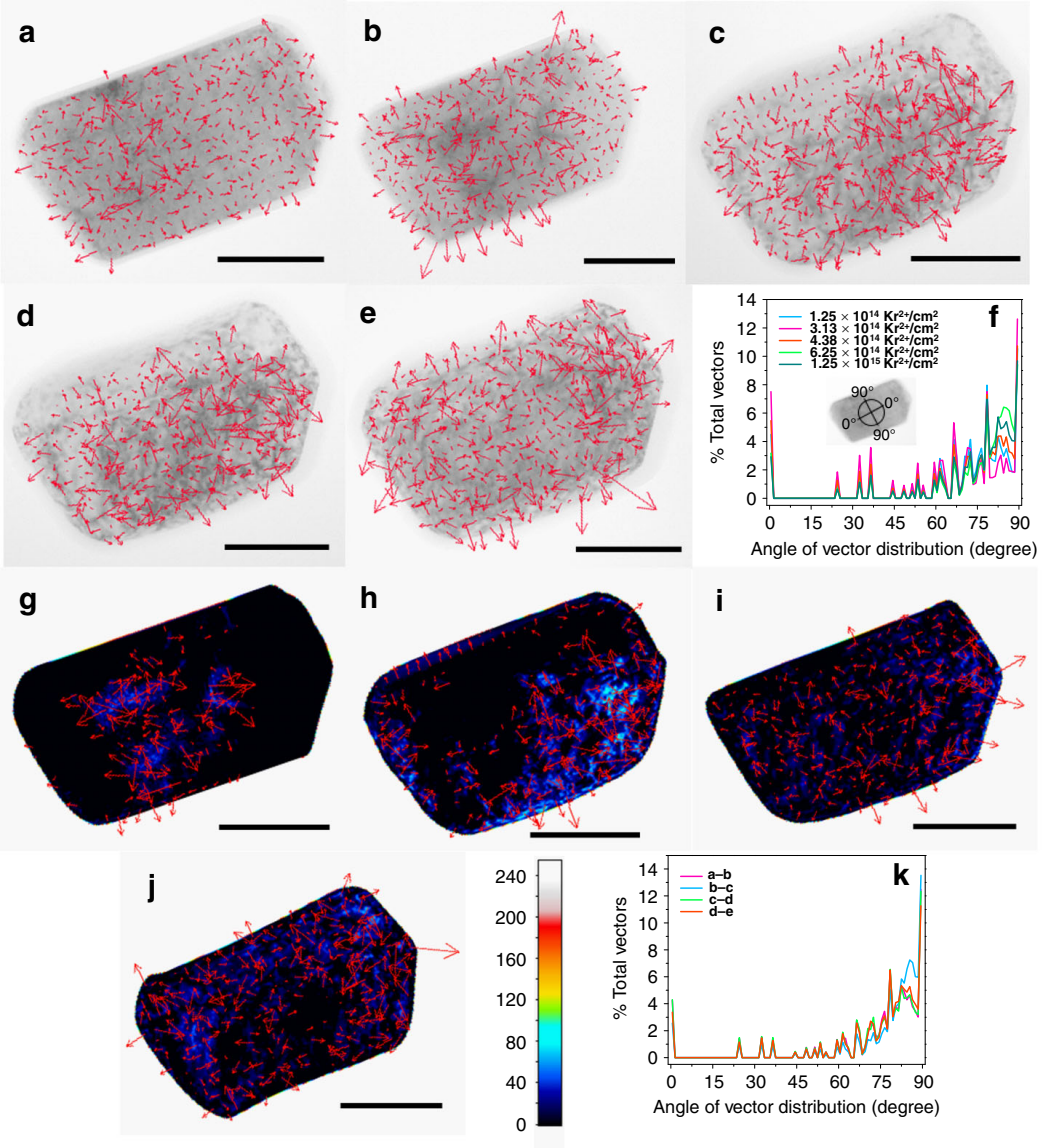
Defect clusters evolution in LiNiO_2_ under Kr ion irradiation. Gradient vector calculated and superimposed on the bright-field two-beam image of a LiNiO_2_ particle irradiated at the fluence of **a** 1.25 × 10^14^ Kr^2+^/cm^2^, **b** 3.13 × 10^14^ Kr^2+^/cm^2^, **c** 4.38 × 10^14^ Kr^2+^/cm^2^, **d** 6.25 × 10^14^ Kr^2+^/cm^2^, and **e** 1.25 × 10^15^ Kr^2+^/cm^2^ at room temperature. The bright-field images are taken from the [100] zone axis. All the scale bars in the image a–e correspond to a length of 100 nm. **f** Distribution of the gradient vectors of images a–e against the angle of the gradient vector. The inset shows the scheme of how the angle of the gradient vector was defined. Dynamic defect evolution in a LiNiO_2_ particle with increasing fluence of Kr ion irradiation (**g**–**j**). The dynamic defect evolution is studied through the subtraction of the image acquired at higher irradiation dose from that of the lower irradiation dose (e.g., image b subtracted from image a). Defect evolution (**g**) from image a to image b, **h** from image b to image c, **i** from image c to image d, and **j** from image d to image e. All the scale bars from image g to image j correspond to a length of 100 nm. The color bar shows the corresponding values of the subtracted pixels after the subtracted grayscale image was converted to an RGB image. **k** Distribution of the gradient vector of images g–j against the angle of the gradient vector.

Defect evolutions in Na_2/3_Fe_1/2_Mn_1/2_O_2_ and LiNiO_2_ are monitored through bright-field two-beam imaging with increasing fluence of Kr ion irradiation. The defect clusters are manifested as black spots in the images since they diffract more beam away from the particle^62^. These grayscale images enable the mapping of defect clusters distribution and propagation under irradiation by performing statistical analysis through pixel by pixel gradient vector computation (Figs. 4 and 5). In a grayscale image, the pixels are composed of either black, white or various shades of gray colors. A number is assigned to the pixels with black color having a value of 0, white color having a value of 255, and different shades of gray colors being assigned values in between (color bar in Fig. 4a). A certain pixel will be surrounded by two pixels in each of the *x*- and *y*-direction (Fig. 4a). Each gradient vector is computed by the partial gradient vectors in both directions. The partial gradient vectors represent brightness changes (calculated in terms of pixel values) in either the *x*-direction or the *y*-direction. The final vector ($\vec g$) is the sum of the two partial vectors (Fig. 4a). This gradient vector represents the overall directional change in brightness from a certain pixel in consideration. The equations listed below define the partial gradient vectors, the final gradient vector, and the size of the final vector:

1 $$\vec g_x = {\frac{\delta \vec f} {\delta x}} \left( {{\mathrm{gradient}}\,{\mathrm{in}}\,{\mathrm{the}}\,{x}\,{\mathrm{direction}}} \right),$$

2 $$\vec g_y ={\frac{\delta \vec f} {\delta y}} \left( {{\mathrm{gradient}}\,{\mathrm{in}}\,{\mathrm{the}}\,{y}\,{\mathrm{direction}}} \right),$$

3 $$\vec g = \vec g_{x} + \vec g_{y}\;(\vec g\,\,{\mathrm{is}}\,{\mathrm{the}}\,{\mathrm{final}}\,{\mathrm{gradient}}\,{\mathrm{vector}}),$$

4 $$\left| {\vec g} \right| = (|\vec g_{x}|^2 + |\vec g_{y}|^2)^{1/2}\;\left( {{\mathrm{size}}\,{\mathrm{of}}\,{\mathrm{the}}\,{\mathrm{gradient}}\,{\mathrm{vector}}} \right).$$

Here, $\frac{{\partial \vec f}}{{\partial x}}$ and $\frac{{\partial \vec f}}{{\partial y}}$ mean the change in pixel values in the *x*-direction and *y*-direction, respectively. The angle (*θ*) of the gradient vector is defined with respect to the Na/Li ion diffusion channel (along the *y*-direction in Fig. 4a). The angle of the gradient vector is defined in such a way that if any vector is along the Na/Li ion diffusion channel, the angle will be 0°. If the vector is perpendicular to the diffusion channel, the angle will be 90° (inset of Fig. 4g and Supplementary Fig. 15). The size of the gradient vectors depends on the magnitudes of the partial gradient vectors (Eq. 4).

The physical meaning of the gradient vector is explained in more details in the Supplementary Discussion and Supplementary Figs. 16–21. In short, the gradient vector is pointing to the direction of non-defect to defect transition or defect to non-defect transition because it shows the direction of the most change in the pixel value, that is, the brightness. The angle of the gradient vector (*θ*) with respect to the Na ion diffusion channel (*y*-direction in Fig. 4a and along 0° in the inset of Fig. 4g) enables the statistical representation of the defect cluster distribution and propagation (Fig. 4g, k). Since every pixel has only two directions associated with it (*x*- and *y*-direction), an angle of the gradient vector of >45° (defined according to the inset in Fig. 4g) means that the larger partial gradient of the pixels is along the *x*-direction, causing the vector to lean closer to the *x*-direction than to the *y*-direction (see Supplementary Discussion and Supplementary Figs. 18 and 21 for more details). Then, it can be conferred that the defect clusters are more likely to terminate in the *x*-direction and align along the *y*-direction because the most change in brightness (calculated in terms of pixel values) is along the *x*-direction. For angles lesser than 45°, the alignment of the defect clusters will be the opposite.

First, we compute the gradient vectors on the bright-field two-beam images of a particle of Na_2/3_Fe_1/2_Mn_1/2_O_2_ (Fig. 4b–e) at various fluences of Kr ion irradiation at room temperature in order to understand the distribution of the defect clusters. Gradient analysis is not performed on the particle at the fluence of 6.25 × 10^14^ Kr^2+^/cm^2^ because the particle is fully amorphous at that irradiation dose (Fig. 4f). The population of the gradient vectors against the angle of the vectors shows if there is any preferential direction of the defect cluster distribution on the particle (Fig. 4g). Inspecting the population of the gradient vectors against the angle, one can notice that most of the vectors have an angle of >45°, with almost 90% of the vectors having an angle of 60° or higher (Supplementary Fig. 22a). This means that most of the defect clusters are more preferentially distributed in the direction of the Na ion diffusion channel (along the *y*-direction) because the larger gradient is in the other direction (Supplementary Figs. 17–21). The higher fluence of Kr ion irradiation can induce more defects and the defects can be diffusive in nature as well^63,64^. Hence, it is important to understand the propagation of the defect clusters under irradiation. We analyzed the defect cluster propagation by the subtraction of the image at a higher fluence from that of a lower fluence (e.g., Fig. 4c subtracted from Fig. 4b) according to the scheme shown in Supplementary Fig. 23. This is followed by similar gradient vector computation and conversion of the grayscale image to an RGB image (Fig. 4h–j). The distribution of the gradient vectors against the angle (Fig. 4k) shows that most of the vectors have an angle >45°, meaning the propagation of the defect clusters is also preferred on the direction of the Na ion diffusion channel (Supplementary Figs. 17–21). The size of the gradient vectors against the angle can provide further justification on the preferential distribution and propagation of the defect clusters. Supplementary Fig. 24a, b show the size of the gradient vectors against the angle from the gradient computation in Fig. 4b–e and h–j, respectively. The larger sized vectors in these distributions are at angles >45° and the largest vectors in size are at the angle of 90°. The largest vectors at 90° suggest that the biggest gradients among all the vectors are at this angle and the magnitude of the vectors is entirely because of the pixel difference along the *x*-direction (see Supplementary Fig. 18). Furthermore, comparing Fig. 4g, k with Supplementary Fig. 24a, b respectively, one can notice that the largest vectors in size at the angle 90° are also the most substantial in population. These facts combined indicate that many defect clusters prefer the distribution and propagation shown in Supplementary Fig. 18, further providing justification to the preferential alignment of the defect clusters in the direction of the Na ion diffusion channel.

Similar gradient analysis on the particle of LiNiO_2_ is performed at various fluences of Kr ion irradiation at room temperature (Fig. 5a–e). The angle of the gradient vector is defined similarly to that of Na_2/3_Fe_1/2_Mn_1/2_O_2_ (inset of Fig. 5f and Supplementary Fig. 15b). The population of the gradient vector against the angle shows that the majority of vectors have an angle >45°. Similar to Na_2/3_Fe_1/2_Mn_1/2_O_2_, such gradient vector distribution again points towards a preferential distribution of the defect clusters along the direction of the Li ion diffusion channel (Fig. 5f and Supplementary Fig. 22b). Delithiated LiNiO_2_ (charged to 4.5 V against Li^+^/Li) particles also have a similar trend of defect cluster distribution (Supplementary Fig. 25). The gradient analysis on the subtracted images (Fig. 5g–j) and the distribution of the vectors against the angle (Fig. 5k) reveal that the defect clusters tend to propagate preferably in the direction of the Li ion diffusion channel, similar to what we have observed for Na_2/3_Fe_1/2_Mn_1/2_O_2_. Size of the vectors against the angle (Supplementary Fig. 24c,d) shows that the larger sized vectors are distributed at angles >45°, with the largest sized vectors being at the angle of 90°. The largest vectors are also the most substantial in population (compare Fig. 5f,k with that of Supplementary Fig. 24c, d), further suggesting the preferential alignment of the defect clusters along the Li ion diffusion channel. This similar trend of preferential defect evolution in both layered materials points to the possible formation of interstitial-type defect clusters and potentially dislocation loops that are parallel to the Na ion or Li ion layers. Here the interstitial-type defect is broadly defined as TM occupying the interlayer space, similar to that reported for graphite^65,66^. The reason may be that in each material the interlayer space between two transition metal layers is large (Fig. 1a, b). The large space provides free volume to accommodate the radiation-induced interstitial atoms. When interstitials accumulate in the interlayer space, they can form interstitial-type clusters or even an extra plane (dislocation loop) (see schematic in Supplementary Fig. 26). This mechanism is similar to the dislocation loop formation mechanisms in some other layered materials, such as graphite under irradiation^65,66^. In graphite, accumulation of interstitials in between basal planes (graphene layers) can form prismatic dislocation loops that are parallel to the basal planes, leading to lattice expansion in *c*-direction and contraction in *a*-direction^65,66^. The defect clusters or loops can cause lattice distortion^67^, which will cause different contrast in the bright-field images. Therefore, we believe that the large interlayer space in the layered oxide cathodes provides the needed free volume for the growth of the defect clusters or dislocation loops along the Na/Li ion diffusion channel. Furthermore, our conclusion is consistent with the experimental observation of edge dislocations in alkali-ion-layered oxides^68,69^.

### Theoretical explanation of the radiation damage behavior

In line with the earlier works on pyrochlores^29,30^, we attempt to understand the radiation damage behavior of layered oxide cathodes in terms of the antisite defect formation under irradiation. In complex oxides with two types of cations (A and B), antisite defects are formed by exchanging the cations^30^,

5 $${\mathrm{A}}_{\mathrm{A}} + {\mathrm{B}}_{\mathrm{B}} \to + {\mathrm{A}}_{\mathrm{B}} + {\mathrm{B}}_{\mathrm{A}},$$

where the A and B in the normal text represent the two different cations and their subscripts represent the cation sites. The formation of antisite defects is also referred to as “cation disorder”^30^. In pyrochlores (A_2_B_2_O_7_), the lower the antisite defect formation energy, the better the resistance to radiation-induced amorphization^29,30^. This is because if the formation energy is low, the crystal lattice can effectively accommodate a substantial amount of cation disorders by still maintaining the crystallinity. Likewise, if the antisite formation energy is high, the system energy increases significantly with the increasing disorder, which can lead to amorphization. Interestingly, the use of antisite formation energy as a criterion for predicting the radiation tolerance of complex oxides may be materials dependent as exemplified by the opposite correlation of amorphization to antisite formation in MgAl_2_O_4_^70^. For the layered oxide cathodes, it is unknown if such a correlation between the antisite formation energy and radiation tolerance exists.

To establish such case, DFT calculations are conducted to calculate the formation energy of an antisite pair in the layered cathodes. Four simulation systems are used: O3-LiNiO_2_, P2-NaFeO_2_, O3-NaFeO_2_, and P2-Na_2/3_Fe_1/2_Mn_1/2_O_2_. These materials are used as model systems for the two materials studied in our experiment. In addition, P2-NaFeO_2_ and O3-NaFeO_2_ are used to check if the antisite formation energy is sensitive to material polymorph.

**Table 1:** DFT results of the lattice parameters, bandgaps, and antisite formation energies in four model systems.

Materials	System size (atoms)	*a* (Å)	*c* (Å)	Bandgap (eV)	Antisite pair distance (Å)	Antisite pair formation energy (eV)
LiNiO_2_ (O3)	96	2.88 (this work)	14.35 (this work)	2.07	12.0	−0.54/0.23^a^
		2.88 (Exp.)^71^	14.19 (Exp.)^71^			
NaFeO_2_ (O3)	96	3.04 (this work)	16.09 (this work)	1.85	13.5	4.32
		3.03 (Exp.)^72^	16.10 (Exp.)^72^			
NaFeO_2_ (P2)	64	3.03 (this work)	10.81 (this work)	1.84	8.8	4.52
		2.96 (DFT)^73^	10.68 (DFT)^73^			
Na_2/3_Fe_1/2_Mn_1/2_O_2_ (P2)	88	2.97 (this work)	11.15 (this work)	0.54	Fe_1_–Na_1_: 8.6	2.73
		2.93 (Exp.)^47^	11.22 (Exp.)^47^		Fe_2_–Na_2_: 8.9	3.22
					Fe_3_–Na_2_: 9.8	3.00
					Mn_1_–Na_1_: 8.9	4.04
					Mn_2_–Na_1_: 9.0	4.33
					Mn_3_–Na_2_: 8.9	5.05

^a^The 0.23 eV is obtained using a 48-atom system.

We begin with examining the first three systems in which the alkali cations have the full occupancy. Table 1 shows the calculated lattice parameters in the three systems after structural optimization. For O3-LiNiO_2_ and O3-NaFeO_2_, both *a* and *c* lattice constants are in very good agreement with experimental values. For P2-NaFeO_2_, our DFT results are slightly larger than previous DFT results^73^. Although there are no experimental data of perfect P2-NaFeO_2_ for direct comparison, our DFT results are in reasonable agreement with the experimentally determined lattice parameters of P2-Na_2/3_Fe_1/2_M_1/2_O_2_ (*a* = 2.93 Å, *c* = 11.22 Å)^47^. To introduce a pair of antisite defects with a maximized distance between them (to minimize the interaction between the two antisite defects), a Li (or Na) atom near the bottom of each simulation system in the *c*-direction is swapped with a Ni (or Fe) atom at the center (Supplementary Fig. 27). The distance between the two antisite defects in each system is shown in Table 1. The formation energy of an antisite pair (or cation disorder energy) is defined as,

6 $$\Delta E = {E_{\mathrm{antisite}}} - {E_{\mathrm{perfect}}},$$

where *E*_antisite_ is the total energy of the simulation system containing one antisite pair and *E*_perfect_ is the total energy of the perfect system of the same system size. For O3-LiNiO_2_, the antisite pair formation energy is −0.54 eV, indicating a slightly favorable antisite pair formation in this 96-atom system (Table 1), in which the antisite defect concentration is 4.2% (=1/24). Note that the negative antisite formation energy (−0.54 eV in 96-atom system) indicates that a perfect LiNiO_2_ is difficult to obtain due to the spontaneous formation of Li-Ni antisite defects, even in the pristine state. In fact, a few percent of Ni sitting in the Li site is widely reported in the literature^48,74^. In some other LiNiO_2_-based materials, the antisite concentration can be as high as 11.8% (Table 2 in ref. ^75^). Therefore, our DFT results are consistent with these experimental observations. In a separate DFT calculation using a smaller O3-LiNiO_2_ with 48 total atoms, in which the concentration of antisite defects is doubled (i.e., 8.3%), the antisite pair formation energy is 0.23 eV, indicating that antisite defect formation is slightly unfavorable at high antisite concentrations. In either case, the formation of an antisite pair in O3-LiNiO_2_ does not change the system energy significantly, suggesting that O3-LiNiO_2_ can efficiently accommodate radiation-induced antisite defects. In comparison, the calculated formation energy of an antisite pair is much larger in O3-NaFeO_2_ (4.32 eV) and P2-NaFeO_2_ (4.52 eV) (Table 1), regardless of material polymorph. Therefore, from the energetics viewpoint, LiNiO_2_ can accommodate much more radiation-induced antisite defects than NaFeO_2_. In turn, O3-LiNiO_2_ should be more radiation tolerant than either O3 or P2-NaFeO_2_. As discussed below, if we assume P2-NaFeO_2_ can be used as a model system for P2-Na_2/3_Fe_1/2_Mn_1/2_O_2_, our DFT results can be used to explain our experimental observation (see Fig. 2).

Previously, it has been shown that the antisite formation energy (and thus, radiation tolerance) can be correlated with the ionic radius difference between A and B cations in pyrochlores^30^. If the difference is large, the antisite formation energy is high and thus the radiation tolerance is low. As shown below, such rationalization can be extended to layered oxides to predict the resistance to radiation damage and design layered oxide cathodes that are stable under irradiation. For the cations in our battery materials, the effective ionic radii are: Li^+^ (0.76 Å), Ni^3+^ (0.56 Å, 0.60 Å), Na^+^ (1.02 Å), and Fe^3+^ (0.55 Å, 0.645 Å), where the two values for each of Ni^3+^ and Fe^3+^ correspond to low spin and high spin states, respectively^76^. The much smaller ionic radius difference between Li^+^ and Ni^3+^ in LiNiO_2_ than that between Na^+^ and Fe^3+^ in NaFeO_2_ is indeed consistent with the difference in the antisite formation energy between the two systems.

**Fig. 6:**
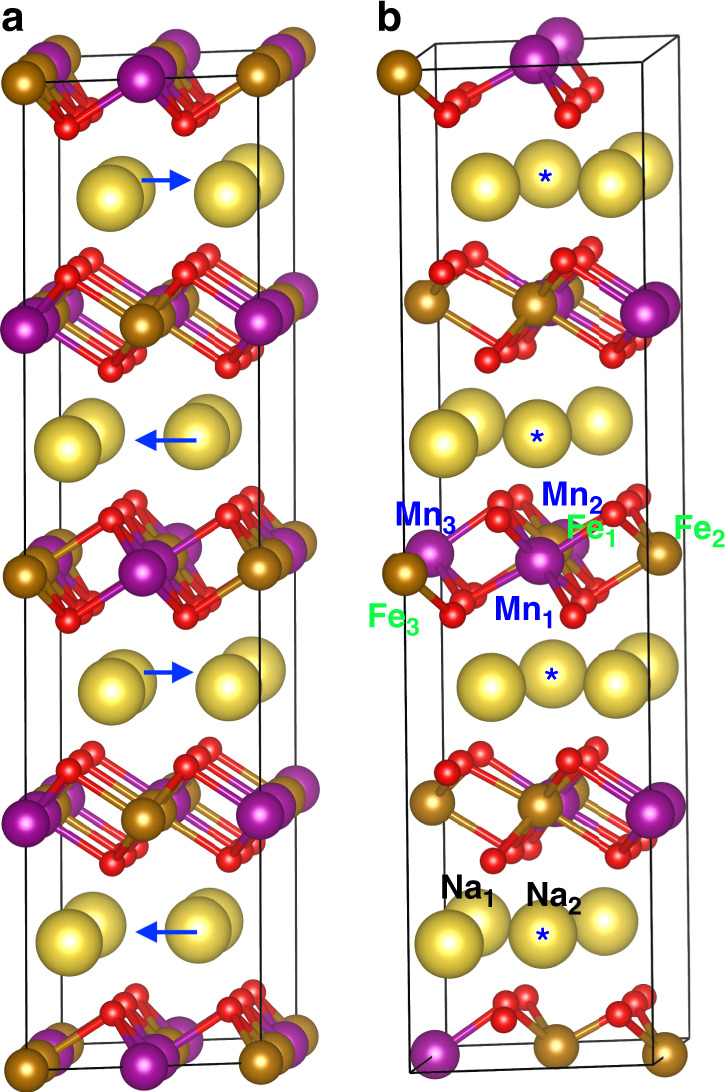
Atomic configurations of P2-Na_2/3_Fe_1/2_Mn_1/2_O_2_ and antisite defect positions. **a** Before structural relaxation. All Na cations are placed at 2*d* sites initially. The blue arrows indicate the moving directions of some Na cations after relaxation. **b** After structural relaxation. The Na cations with an asterisk (*) are those moving to the new 2b sites. The labeled TM and Na cations are those used to create antisite pairs. The two figures show some additional atoms at simulation box boundaries for visualization purpose (based on periodic boundary conditions). Large yellow spheres: Na; medium brown spheres: Fe; medium purple spheres: Mn; small red spheres: O.

As to Mn^3+^, its ionic radius (0.58 Å, 0.645 Å) is nearly identical as Fe^3+^ for each spin state^76^. In P2-Na_2/3_Fe_*x*_Mn_1 − *x*_O_2_, Mn^4+^ and Fe^4+^ may also exist according to the X-ray absorption spectroscopy measurements^77^ and their ionic radii are also similar (0.585 vs. 0.53 Å) ^76^. Therefore, if the ionic radius difference between alkali and TM cations is the key factor for affecting the antisite formation energy (and thus the radiation tolerance), an Mn–Na antisite pair should also have a high antisite formation energy. To prove this hypothesis, Mn–Na and Fe–Na antisite formation energies are directly calculated in P2-Na_2/3_Fe_1/2_Mn_1/2_O_2_. More complex than the ideal P2-NaFeO_2_, the Na cations in Na_2/3_Fe_1/2_Mn_1/2_O_2_ do not have a full site occupancy and the TM layer consists of both Mn and Fe cations. Moreover, it has been shown experimentally that Na cations can stay in two different sites in Na_2/3_Fe_*x*_Mn_1 − *x*_O_2_: 2b (0, 0, 1/4) and 2d (2/3, 1/3, 1/4)^77^, although it is unclear the exact arrangement of Na cations at the two sites. To predict the atomic configuration of P2-Na_2/3_Fe_1/2_Mn_1/2_O_2_, a P2-NaFeO_2_ consisting of 3 × 2 × 2 unit cells (96 atoms in total) is created initially. In each of four TM layers, three out of six Fe cations are replaced by Mn cations so that the Fe:Mn ratio is 1:1 in each TM layers (Fig. 6a). All Na cations are initially placed at the 2*d* sites. Then two out of six Na cations in each of four Na layers are removed. Now the system has 88 atoms in total (16 Na, 12 Fe, 12 Mn, 48 O), which has the same stoichiometry as Na_2/3_Fe_1/2_Mn_1/2_O_2_. After structural relaxation, interestingly, one Na cation in each of four Na layers moves from a 2*d* site to a 2*b* site. The moving directions of these Na cations are illustrated in Fig. 6a and the final configuration is shown in Fig. 6b. The final Na site occupancy factors are 0.5 for 2*d* site and 0.17 for 2*b* site in Na_2/3_Fe_1/2_Mn_1/2_O_2_, which are similar to 0.43 for 2*d* site and 0.26 for 2*b* site in Na_2/3_Fe_1/3_Mn_2/3_O_2_ as determined by experiments^77^. Therefore, our DFT calculation predicts reasonable Na site occupancy factors without any a priori assumptions. In addition, the predicted lattice parameters are also similar to the experimental values, as shown in Table 1.

Due to the complex atomic configuration of P2-Na_2/3_Fe_1/2_Mn_1/2_O_2_, it is expected that the antisite pair formation energy depends on the local atomic environment of each antisite defect. To ensure that our conclusion is not specific to a certain antisite defect configuration, three Fe–Na antisite pairs (Fe_1_–Na_1_, Fe_2_–Na_2_, Fe_3_–Na_2_) and three Mn–Na antisite pairs (Mn_1_–Na_1_, Mn_2_–Na_1_, Mn_3_–Na_2_) are modeled and the original positions of these cations are shown in Fig. 6b. The calculated antisite pair formation energies are shown in Table 1. Similar to O3 or P2-NaFeO_2_, the formation energy of an Fe–Na antisite pair is still high: in the range of 2.73–3.22 eV; the formation energy of a Mn–Na antisite pair is even higher: in the range of 4.04–5.05 eV. The exact cause for the discrepancy between the two types of antisite pairs is unclear. It could be due to different charge states of Fe and Mn cations in P2-Na_2/3_Fe_1/2_Mn_1/2_O_2_, or different local atomic environment of these defects, or the actual ionic radii of Fe and Mn are slightly different from the theoretical predictions by Shannon^76^. Nevertheless, the formation energy of an antisite pair in P2-Na_2/3_Fe_1/2_Mn_1/2_O_2_ is significantly higher than in O3-LiNiO_2_, regardless of the antisite defect type (Table 1). Therefore, our DFT results of antisite pair formation energy as well as the ionic radius difference can be well applied to explain why O3-LiNiO_2_ has a better radiation tolerance than P2-Na_2/3_Fe_1/2_Mn_1/2_O_2_. The DFT results from the P2-Na_2/3_Fe_1/2_Mn_1/2_O_2_ are qualitatively similar as that from the ideal NaFeO_2_ (although the magnitudes are different), indicating the ionic radius difference between TM and alkali cations is a key factor for predicting the antisite formation energy and radiation tolerance. This justifies NaFeO_2_ can be used as a model system for Na_2/3_Fe_1/2_Mn_1/2_O_2_ in terms of radiation tolerance.

**Fig. 7:**
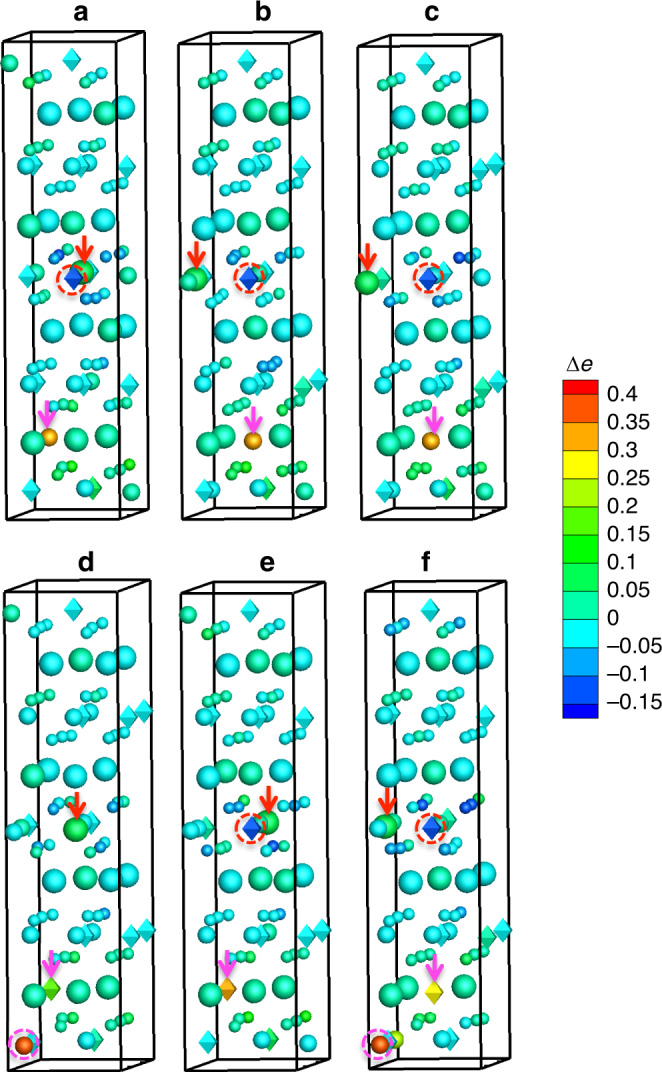
Charge transfer distribution due to antisite defects in P2-Na_2/3_Fe_1/2_Mn_1/2_O_2_. Each atom is colored by the change of its valence electrons with respect to its counterpart in the pristine system. Red and magenta arrows indicate the Na_TM_ and TM_Na_ antisite defects, respectively. Red dashed circles indicate a significant loss of electrons of some nearby Mn cations. Magenta dashed circles indicate a large gain of electrons of some nearby Fe cations. Large spheres: Na; medium spheres: Fe; medium diamonds: Mn; small spheres: O. The antisite defect pairs are: **a** Fe_1_–Na_1_, **b** Fe_2_–Na_2_, **c** Fe_3_–Na_2_, **d** Mn_1_–Na_1_, **e** Mn_2_–Na_1_, and **f** Mn_3_–Na_2_.

Bader charge analysis^78^ for both perfect and defective systems is performed to explain the charge transfer between an antisite defect and its surrounding atoms. Charge transfer takes place due to the antisite defect formation. Hence, it is important to understand whether there is a correlation between the charge transfer and radiation tolerance of a material. Note that in Bader charge analysis, the charge of each atom is represented by the effective amount of valence electrons. Here the charge of each atom in the perfect system is subtracted from its counterpart in the defective system. Such change in valence electrons is used as a qualitative measure to analyze the charge transfer due to the formation of an antisite pair. A positive value in our charge transfer analysis means that the atom gains extra electrons and thus its oxidation state is lowered and vice versa. The results of the first three model systems are shown in Supplementary Fig. 27. In O3-LiNiO_2_, when a Li^+^ replaces Ni^3+^ (Li_Ni_, center of Supplementary Fig. 27a), some nearby Ni and O atoms lose electrons slightly to accommodate the charge difference at the antisite. However, it seems that the charge transfer around the Li_Ni_ antisite is not localized. Similarly, for the Ni_Li_ antisite (bottom of Supplementary Fig. 27a), the charge transfer is also delocalized. Here localized charge transfer means that the charge transfer is mainly concentrated at the antisite defect itself or its nearest neighbors; delocalized charge transfer means that the charge transfer spreads beyond this range. In O3-NaFeO_2_, the oxygen atoms around the Na_Fe_ (center of Supplementary Fig. 27b) lose electrons to accommodate the change from Fe^3+^ to Na^+^. The charge transfer is more localized than that near Li_Ni_. The result suggests that the oxidation state of some oxygen atoms may change from O^2−^ to O^−^. For the Fe_Na_ antisite (bottom of Supplementary Fig. 27b), the charge transfer is also localized and the Fe gains electrons. In addition, another nearby Fe atom also gains electrons. The result suggests that the oxidation state of Fe at or near the Fe_Na_ antisite may change from Fe^3+^ to Fe^2+^ to accommodate the antisite defect. In the P2-NaFeO_2_, interestingly, the charge transfer has a mixed behavior. Near the Na_Fe_ (center of Supplementary Fig. 27c), the charge transfer seems to be delocalized. At the Fe_Na_ (bottom of Supplementary Fig. 27c), the charge transfer seems to be localized at the antisite—Fe gains electrons and its oxidation state may change from Fe^3+^ to Fe^2+^. The charge transfer in P2-Na_2/3_Fe_1/2_Mn_1/2_O_2_ is more complex, as shown in Fig. 7. For the systems containing an Fe–Na antisite pair (Fig. 7a–c), some nearby oxygen anions around the Na_Fe_ (at the middle of each figure in the vertical direction) lose electrons. Interestingly, one nearby Mn cation also loses some electrons, as indicated by the red dashed circle in each figure. This suggests that the oxidation state of the nearby Mn cation may increase to accommodate the charge difference between Na^+^ and Fe^3+^. At the Fe_Na_ antisite (near the bottom of each figure), the Fe_Na_ antisite defect gains some electrons, suggesting the oxidation state of Fe at the antisite may decrease. For the systems containing an Mn–Na antisite pair (Fig. 7d–f), oxygen anions behave similarly as the cases with a Fe–Na antisite pair. Near the Na_Mn_ antisite (at the middle of each figure), a nearby Mn also tends to lose electrons, except in Fig. 7d. At the Mn_Na_ antisite (near the bottom of each figure), the Mn_Na_ antisite defect gains some electrons, indicating the Mn may lower the oxidation state. In two cases (bottom of Fig. 7d, f), a nearby Fe also gains some electrons. Overall, it seems that the oxidation state of Mn can either increase or decrease to accommodate antisite defects, while the oxidation state of Fe always tends to decrease. The different charge transfer behavior between Fe and Mn cations may shed a light on the experimental observation that Fe^4+^ is more difficult to form than Mn^4+^ in P2-Na_2/3_Fe_1/2_Mn_1/2_O_2_ during charging^77^. The above analysis shows that the detailed charge transfer/redistribution mechanism is material specific. We have not observed a clear correlation between the detailed charge transfer mechanism and antisite formation energy. If other electronic configurations are used in our DFT modeling, the details of the charge transfer process may change somewhat. However, the trend of the antisite formation energy should not change significantly because the difference in ionic radius between TM and alkali cations is the key factor for determining the antisite defect formation energy. Meanwhile, our density of states calculations suggest that the introduction of antisite defects might give all these defective materials more metallic-like characteristics as their bandgaps disappear (Supplementary Figs. 28 and 29). However, such a prediction needs further experimental validation, which is beyond the scope of this work.

## Discussion

In summary, our work has unveiled the fundamental mechanisms of defect evolution and structural transformations in Na- and Li-layered cathodes, promoted by high-energy Kr ion irradiation. High-energy ion irradiation such as Kr ion is different from electron irradiation in TEM characterization. The structural damage due to electron irradiation on battery materials has been reported to mostly induce structural transformations on the surface and near-surface region of the particle^53,79^. As evidenced in our work, Kr ion irradiation can induce structural transformations within hundreds of nanometers of a cathode particle at a short duration of time. Moreover, electron irradiation mostly produces point defects or small defect clusters^80,81^. However, Kr ion irradiation can produce much larger dislocation loops and voids^26^. Hence, utilizing Kr ion irradiation allows us to truly compare the radiation tolerance of the Li- and Na-layered cathodes in extreme environments. Our experimental results suggest that Li-layered cathode, for example, LiNiO_2_ is more resistant to Kr ion irradiation-induced structural damage than Na-layered cathode, for example, Na_2/3_Fe_1/2_Mn_1/2_O_2_, which can be associated with the easiness of the cationic antisite defect formation in the former. Our theoretical analysis has revealed that the antisite defect formation energy is significantly smaller in LiNiO_2_ because of the much smaller difference in ionic radius between Li^+^ and Ni^3+^ than those between Na^+^ and Fe^3+^/Mn^3+^/Mn^4+^, allowing better accommodation of radiation damage than P2-Na_2/3_Fe_1/2_Mn_1/2_O_2_. The findings suggest that structural transformations in both Li- and Na-layered cathodes under irradiation follow the similar principle of cationic antisite defect formations, similar to pyrochlore oxides. Hence, our study provides a valuable guideline for designing stable layered cathodes under extreme conditions, such as outer space exploration and nuclear power industries. Between different layered oxides (A_*x*_TMO_2_, where A is alkali ion, and TM is transition metal ion), a material with a smaller difference in the ionic size between A and TM will have a smaller cationic antisite defect formation energy and will be more resistant to radiation damage. Resistance to radiation damage is also closely related to the temperature^82,83^. Like in many other oxide ceramics^35^, high temperature can lessen the severity of structural transformations of Na-layered oxide by accelerating the annihilation of radiation-induced defects through the recombination of vacancies and interstitials^82^. Enhanced defect annihilation at high temperature should enhance the structural stability of Li-layered oxide as well. Instead of a direct crystalline to amorphous transformation, our study shows that Na-layered oxide undergoes a phase transformation to a spinel-type structure at high temperature. Some irradiation-resistant pyrochlores, for example, Gd_2_Zr_2_O_7_ also undergoes a phase transformation to a fluorite-type structure^25^. Such phase transformation is indicative of an intermediate phase formation rather than full disordering to an amorphous phase. Our study informs the radiation damage of battery materials at a broad range of temperatures and establishes the fact that the resistance to radiation damage of layered cathodes increases with the elevation of temperature. Thus, our findings provide a comprehensive guideline for predicting radiation tolerance of layered cathodes. Meanwhile, our mathematical analysis on the bright-field images quantitatively mapped the distribution and propagation of defect clusters under irradiation and revealed that defect clusters tend to align along the direction of the Na/Li ion diffusion channels (*a*–*b* plane). The preferential defect alignment is likely due to the formation of interstitial-type dislocation loops in the interlayer space between transition metal layers, in which a large free volume is available to accommodate the accumulation of the interstitials. Such dynamics of defect evolution (e.g., the formation and accumulation of vacancies and interstitials) under ion irradiation shares similar attributes to that of defect evolution in layered cathodes on electrochemical cycling (e.g., vacancies and interstitials formation through oxygen evolution and ion migration)^57,84,85^. Point defects such as vacancies and interstitials can largely influence the electrochemical performance of layered cathodes. Interstitials resulting from the transition metal migration are reported to cause voltage decay in high-energy Li-rich layered cathode materials^43^. Voltage decay results in subpar energy efficiency, which hinders the commercialization of these promising cathode materials. A large quantity of interstitial defects can cause phase transformation from layered to spinel or rocksalt phase^42^, leading to transition metal dissolution, cathode particle cracking, and high electrochemical impedance development^86^. Extensive material damage due to phase transformation and oxygen evolution may induce amorphization, leading to accelerated electrochemical performance degradation^87^. The aforementioned structural and chemical stability issues can be alleviated to some degree through doping chemistry^48^. Radiation creates a high concentration of point defects. The impacts of irradiation-induced defects on the electrochemical performance of Li- and Na-layered cathodes and whether doping can play a role in the stability under irradiation deserve further studies in the future.

## Methods

### Materials synthesis

Na_2/3_Fe_1/2_Mn_1/2_O_2_ was synthesized by a simple solid-state synthesis method with the stoichiometric amount of precursors Na_2_CO_3_, Fe_2_O_3_, and Mn_2_O_3_ being ball milled at a rate of 35 Hz for 6 h. The precursor was calcined in a box furnace at 900 °C for 12 h, followed by rapid quenching and stored in the glovebox. The precursor of the LiNiO_2_ was synthesized by the precipitation of the salt solution of NiSO_4_·6H_2_O by a base solution of NaOH and NH_4_OH. The precipitated Ni(OH)_2_ was collected and dried in a vacuum oven overnight at 105 °C. The precursor powder was mixed with stoichiometric amount of LiOH and calcined in a tube furnace under airflow at 450 °C for 2 h, followed by 675 °C for 6 h to get the final LiNiO_2_ powder. The powder of LiNiO_2_ was stored in the glovebox for further usage.

### Electrochemical characterization

Electrodes of Na_2/3_Fe_1/2_Mn_1/2_O_2_ were casted on a carbon-coated aluminum foil by making a slurry of 80% active material, 15% carbon black, and 5% poly(vinylidene difluoride) (PVDF). Discs of 10 mm diameter were cut from the casted slurry and dried in a vacuum oven overnight at 120 °C. Electrodes of LiNiO_2_ were casted in a similar way with a slurry of 90% active material, 5% carbon black, and 5% PVDF and discs of 10 mm diameter were cut and dried in a vacuum oven overnight at 120 °C. CR2032 coin cells with Na anode and Na_2/3_Fe_1/2_Mn_1/2_O_2_ cathode were assembled with 1.0 M NaClO_4_ in propylene carbonate as the electrolyte and Whatman glass fiber (1827-047934-AH) as the separator. A specific current density of 180 mA/g (defined as 1C) was used to calculate the charge and discharge current density. Li half cells were assembled from CR2032 coin cell parts with Li metal as the anode, LiNiO_2_ as the cathode, and the Whatman glass fiber as the separator. One mole of LiPF6 dissolved in ethylene carbonate and ethyl methyl carbonate with 2 wt% vinylene carbonate was utilized as the electrolyte. A specific current density of 200 mA/g was used to calculate the current density at 1 C. LANDT battery cycler was utilized to collect the electrochemical cycling data.

### Materials irradiation and characterization

In situ Kr ion irradiation and simultaneous TEM observation was performed in an intermediate voltage electron microscope (IVEM-Tandem facility) at Argonne National Laboratory. A Kr ion energy of 1 MeV was utilized for irradiation and an electron beam energy of 300 keV (Hitachi-9000) was utilized for imaging. Na_2/3_Fe_1/2_Mn_1/2_O_2_ was irradiated at a total fluence of 6.25 × 10^14^ Kr^2+^/cm^2^ and LiNiO_2_ was irradiated at a total fluence of 1.25 × 10^15^ Kr^2+^/cm^2^ at room temperature. A total fluence of 1.25 × 10^14^ Kr^2+^/cm^2^ was utilized for irradiation at −173 °C and a total fluence of 1.25 × 10^15^ Kr^2+^/cm^2^ was utilized for irradiation at 200 °C. Electron irradiation for TEM imaging was in the direction “into the plane of the paper.” Kr ion irradiation was incident at an angle of 30° with respect to the electron irradiation. The fluence rate of Kr ion irradiation was 6.25 × 10^10^ Kr^2+^/cm^2^/s. The charge of Kr ion is marked with 2+ (++) but the charge number does not impact the material damage. Irradiation was stopped at various intermediate fluence for defect imaging and acquiring ED patterns. Morphology of the materials was acquired in a scanning electron microscope (LEO FESEM) operating with an accelerating voltage of 5 kV. The XRD patterns of the materials were collected in a benchtop Rigaku Miniflex II X-ray diffractometer utilizing a Cu Kα radiation at a wavelength of 1.54 Å. For acquiring the pattern, a step size of 0.02° and a scan rate of 1°/min were used.

### Theoretical calculation

All DFT calculations were conducted in Vienna Ab initio Simulation Package (VASP) software. The projector-augmented-wave (PAW) pseudopotential^88^ was used to describe the electron-core interaction. The Perdew–Burke–Ernzerhof (PBE) functional^89^ of gradient approximation was used for the electron exchange-correlation energy. In this work, the standard PAW-PBE potentials for Li, Na, Ni, Fe, Mn, and O available in VASP were utilized. In all calculations, the plane wave cutoff energy was set to 520 eV, Gaussian smearing was used with a smear width of 0.05 eV, and the energy convergence criterion was set to 10^−4^ eV. Spin polarization effect is included and the initial magnetic moment is set to 2*μ*_B_ for Ni, 6*μ*_B_ for Fe, and 6*μ*_B_ for Mn. To treat the strongly correlated *d* electrons in Ni, Fe, and Mn, DFT + U method was used in which the Hubbard correction parameter (*U*_eff_) was set to 5.96 eV for Ni^89^, 5.2 eV for Fe^90^, and 4.0 eV for Mn^91^. Each of the first three simulation systems consists of 2 × 2 × 2 unit cells and its number of total atoms was shown in Table 1. The P2-Na_2/3_Fe_1/2_Mn_1/2_O_2_ consists of 3 × 2 × 2 unit cells (88 atoms). The *k*-point mesh was a gamma-centered grid with 5 × 5 × 2 for O3-LiNiO_2_, 5 × 5 × 2 for O3-NaFeO_2_, and 4 × 4 × 2 for P2-NaFeO_2_ and P2-Na_2/3_Fe_1/2_Mn_1/2_O_2_.

The gradient vector calculation was performed in the commercial software package Avizo and the vector size and distribution against the angle histograms were calculated in MATLAB. The size of each pixel on the gradient vector computation was 1.124 nm × 1.124 nm.

## Supplementary information

Supplementary Information

Peer Review

## Data Availability

The data supporting the findings of the study are available from the corresponding authors upon reasonable request.

## Code availability

The codes supporting the findings of the study are available in the following link: http://github.com/mrahman4179/Defect-Gradient-vector-analysis/blob/master/Gradient%20vector%20analysis.m.
