# Supplementary material for: Defect and structural evolution under high-energy ion irradiation informs battery materials design for extreme environments
Source: Nat Commun. 2020 Sep 11;11:4548. doi: 10.1038/s41467-020-18345-4 (PMC7486889; doi:10.1038/s41467-020-18345-4)
Supplement: Supplementary file 1 — Supplementary Information [file 41467_2020_18345_MOESM1_ESM.pdf]

Defect and structural evolution under high-energy ion irradiation informs battery materials design for extreme environments

Rahman et al.

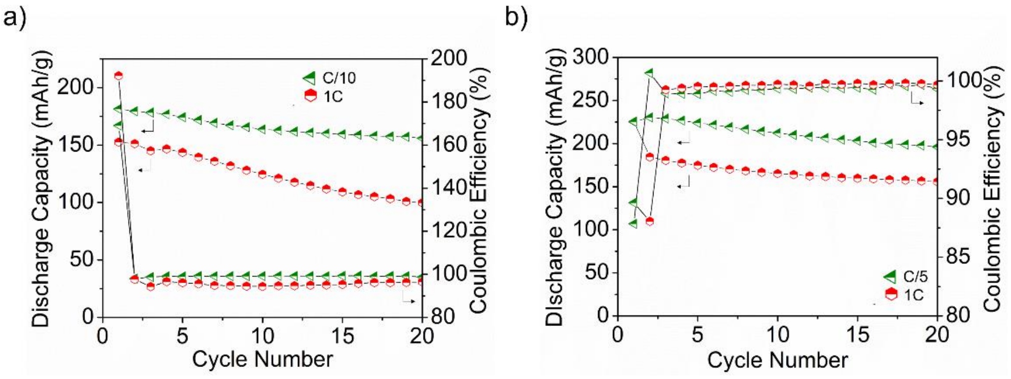

Supplementary Figure 1. Capacity retention and Coulombic efficiency of (a) $Na_{2/3}Fe_{1/2}Mn_{1/2}O_2$, and (b) $LiNiO_2$ in Na half cell and Li half cell, respectively.

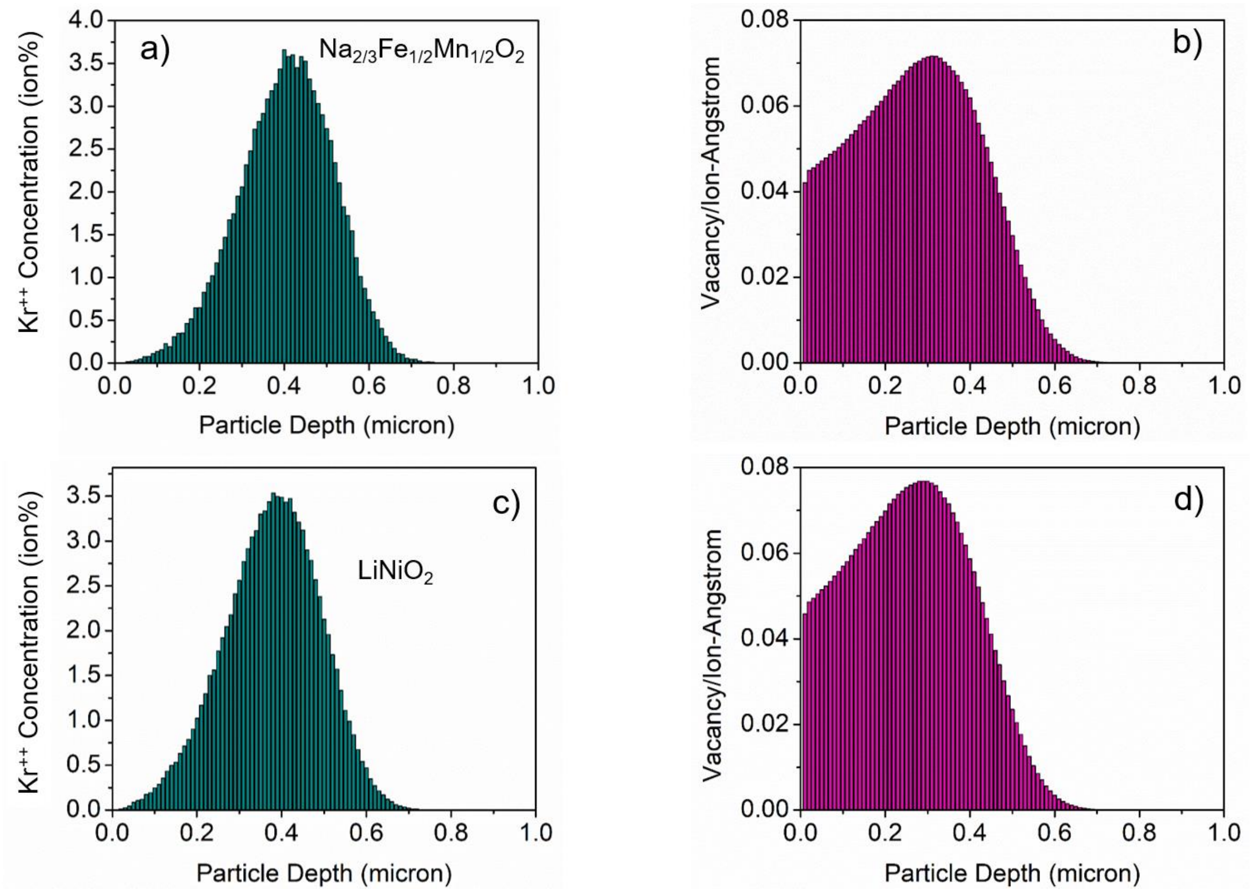

Supplementary Figure 2. SRIM simulation of Kr ion concentration (a) and damage profile (b) of $Na_{2/3}Fe_{1/2}Mn_{1/2}O_2$. SRIM simulation of Kr ion concentration (c) and damage profile (d) of $LiNiO_2$. A total number of $1.0x10^5$ Kr ions were utilized for simulation in $Na_{2/3}Fe_{1/2}Mn_{1/2}O_2$ and a total number of $2.0x10^5$ Kr ions were utilized for simulation in $LiNiO_2$. The energy of the Kr ion was 1 MeV.

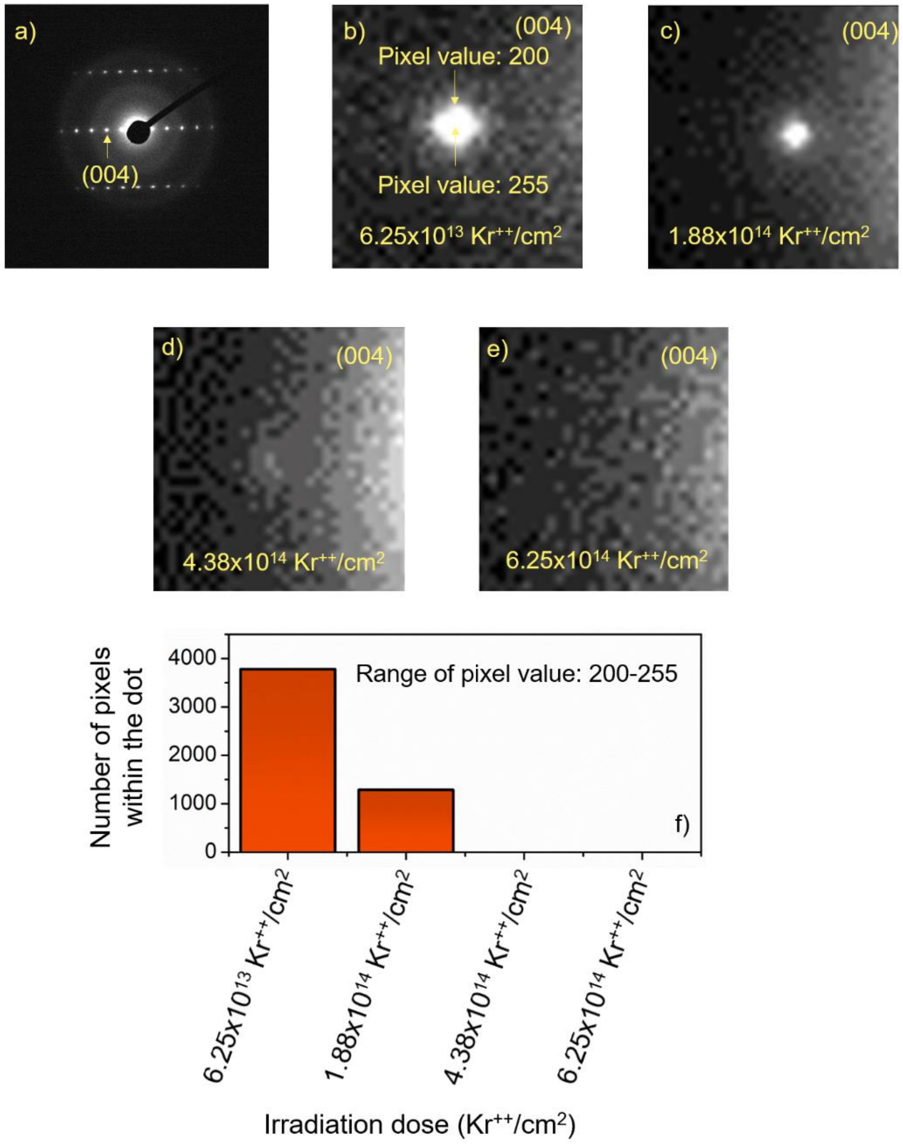

Supplementary Figure 3. Evolution of the crystallinity in $Na_{2/3}Fe_{1/2}Mn_{1/2}O_2$ measured as a function of the brightness of the diffraction dots with increasing fluence of Kr ion irradiation. The images are grayscale images with the brightest pixel corresponds to a white color with the pixel value of 255 and the darkest pixel corresponds to a black color with the pixel value of 0. (a) Electron diffraction of a particle of $Na_{2/3}Fe_{1/2}Mn_{1/2}O_2$. Evolution of the brightness of the dot corresponding to the (004) lattice plane at the fluence of (b) 6.25×10^{13} Kr^{++}/cm^2, (c) 1.88×10^{14} Kr^{++}/cm^2, (d) 4.38×10^{14} Kr^{++}/cm^2, and (e) 6.25×10^{14} Kr^{++}/cm^2. (f) Histogram showing the evolution of the brightness of the diffraction dot for the (004) lattice plane. In image b, the range of pixel value within the dot for (004) plane was 200-255. The brightness is measured in terms of how many pixels the dot contains within the same range starting from image b to image e.

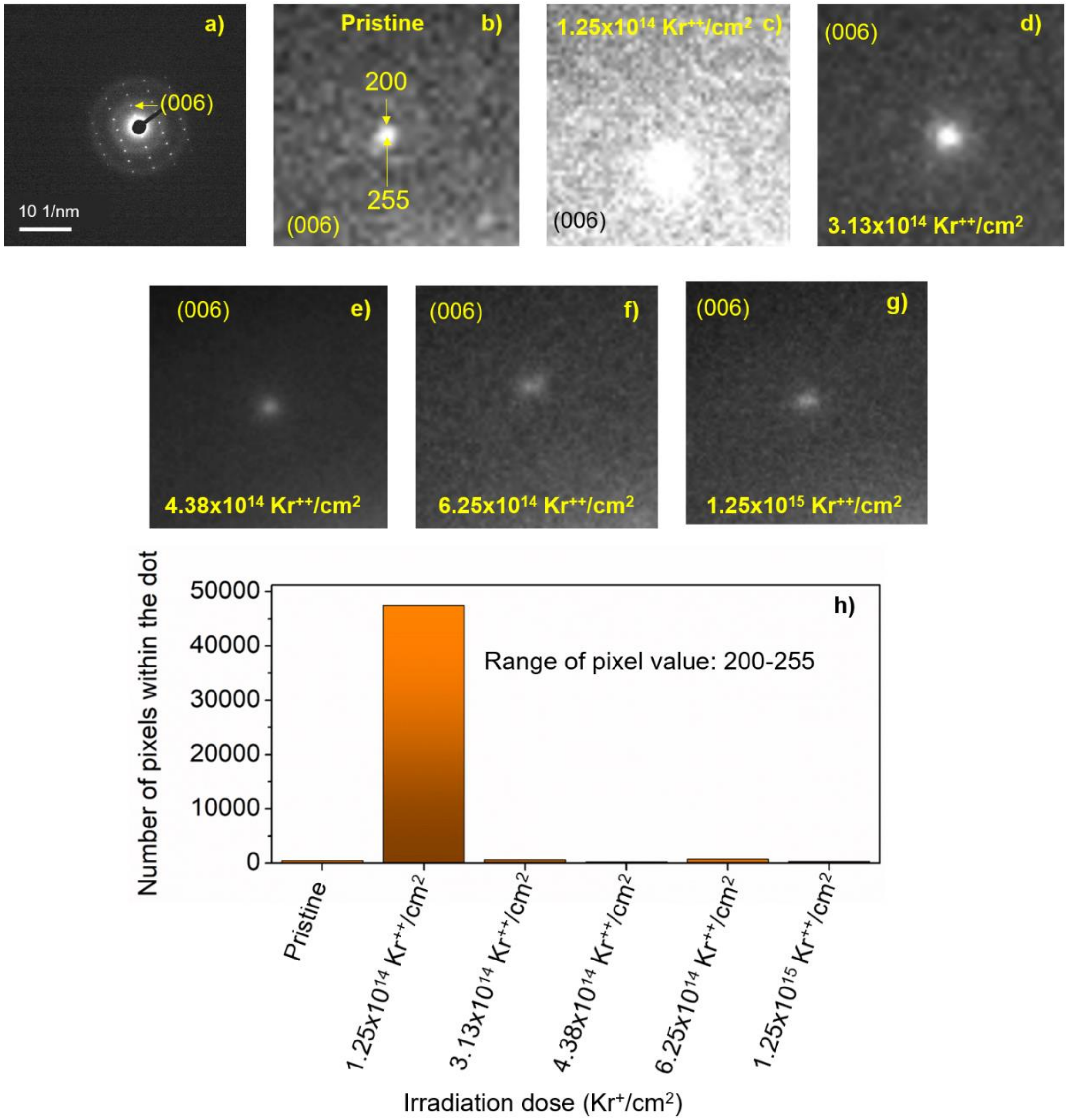

Supplementary Figure 4. Evolution of the crystallinity in $LiNiO_2$ measured as a function of the brightness of the diffraction dots with increasing fluences of Kr ion irradiation. The images are grayscale images with the brightest pixel corresponds to a white color with the pixel value of 255 and the darkest pixel corresponds to a black color with the pixel value of 0. (a) Electron diffraction of a particle of $LiNiO_2$. Evolution of the brightness of the dot corresponding to the (006) lattice plane (b) before irradiation and at the fluence of (c) 1.25x10^{14} Kr^{++}/cm^2, (d) 3.13x10^{14} Kr^{++}/cm^2, (e) 4.38x10^{14} Kr^{++}/cm^2, (f) 6.25x10^{14} Kr^{++}/cm^2, and (g) 1.25x10^{15} Kr^{++}/cm^2. (h) Histogram showing the evolution of the brightness of the diffraction dot for the (006) lattice plane. In image b, the range of pixel value within the dot for (006) plane was 200-255. The brightness is measured in terms of how many pixels the dot contains within the same range starting from image b to image g.

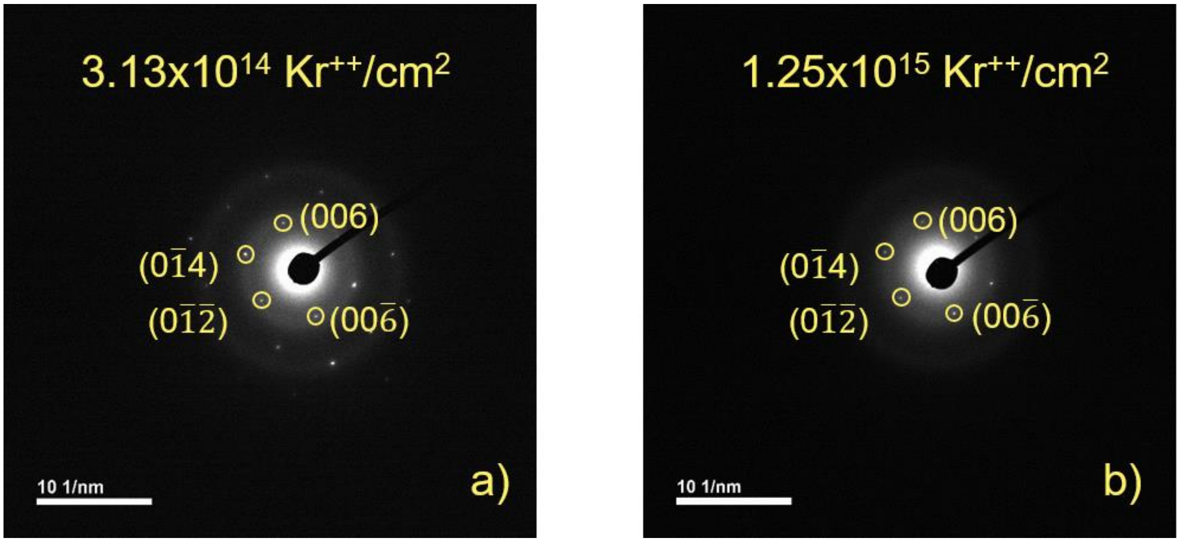

Supplementary Figure 5. Electron diffraction pattern of $LiNiO_2$ at the fluence of (a) $3.13x10^{14}$ Kr^{++}/cm^2, and (b) $1.25x10^{15}$ Kr^{++}/cm^2.

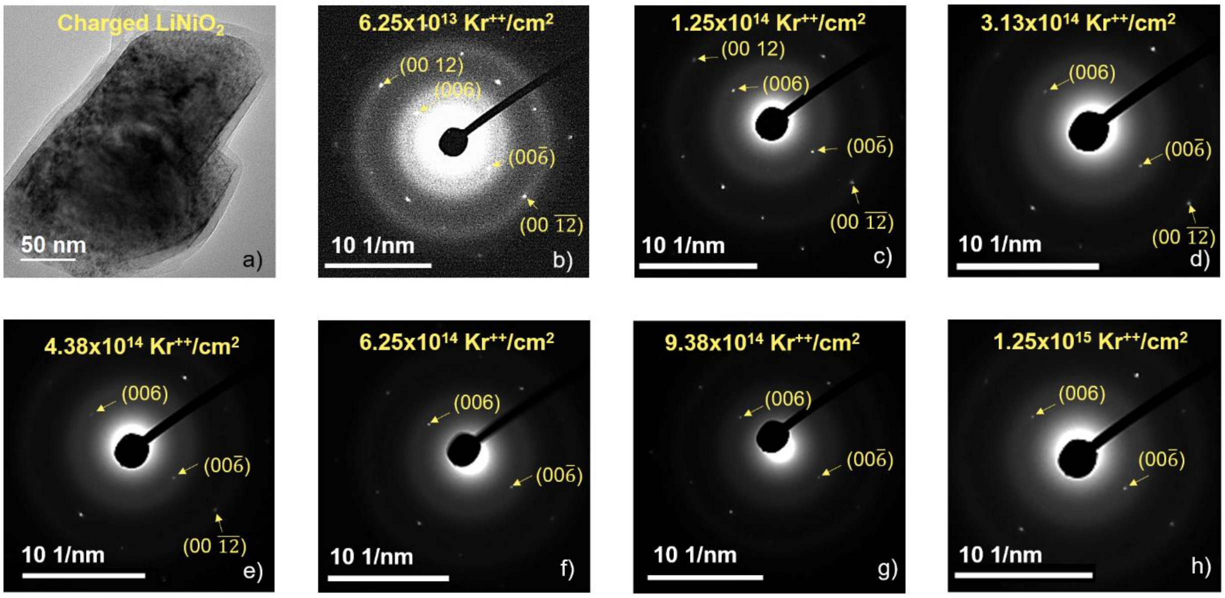

Supplementary Figure 6. Electron diffraction patterns of Kr ion irradiated delithiated $LiNiO_2$ (charged) at room temperature. (a) The delithiated $LiNiO_2$ that was irradiated. Electron diffraction of delithiated $LiNiO_2$ at the fluence of (b) $6.25x10^{13}$ Kr^{++}/cm^2, (c) $1.25x10^{14}$ Kr^{++}/cm^2, (d) $3.13x10^{14}$ Kr^{++}/cm^2, (e) $4.38x10^{14}$ Kr^{++}/cm^2, (f) $6.25x10^{14}$ Kr^{++}/cm^2, (g) $9.38x10^{14}$ Kr^{++}/cm^2, and (h) $1.25x10^{15}$ Kr^{++}/cm^2.

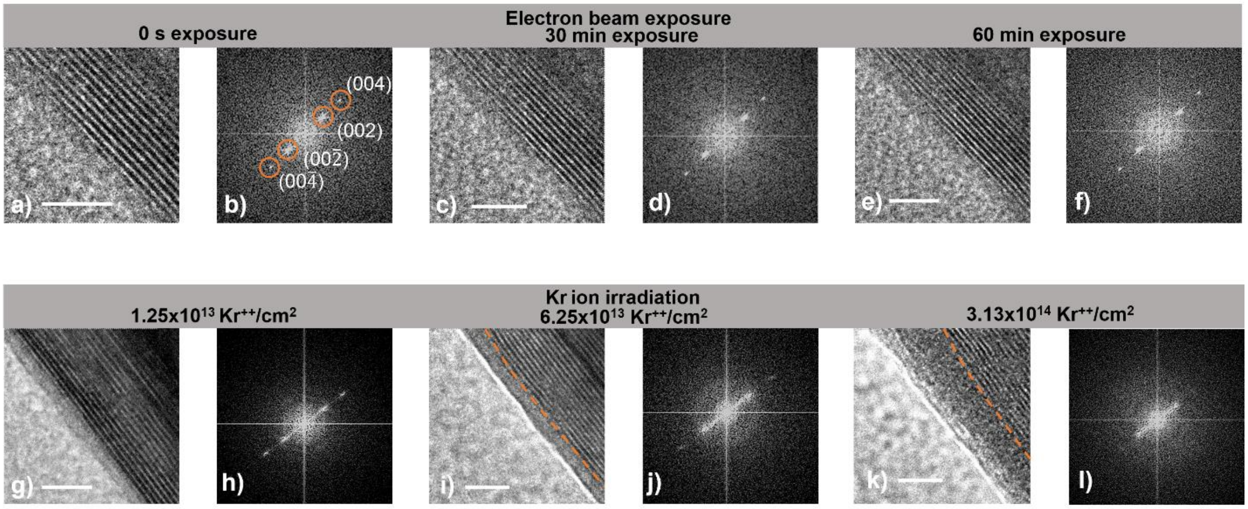

Supplementary Figure 7. Evaluation of the change in crystallinity of $Na_{2/3}Fe_{1/2}Mn_{1/2}O_2$ under electron beam utilized for TEM imaging (a-f) and Kr ion irradiation (g-l). (a) TEM image before prolonged exposure to electron beam is commenced. (b) FFT of the image in a. (c) TEM image after exposure to the electron beam for 30 minutes. (d) FFT of the image in c. (e) TEM image after exposure to the electron beam for 60 minutes. (f) FFT of the image in e. (g) TEM image at the fluence of $1.25x10^{13}$ Kr^{++}/cm^2. (h) FFT of the image in g. (i) TEM image at the fluence of $6.25x10^{13}$ Kr^{++}/cm^2. (j) FFT of the image in i. (k) TEM image at the fluence of $3.13x10^{14}$ Kr^{++}/cm^2. (l) FFT of the image in k. The dashed lines in image i and k are utilized to show the growth of the amorphous region in the particle. All scale bars in the images correspond to a length of 5 nm.

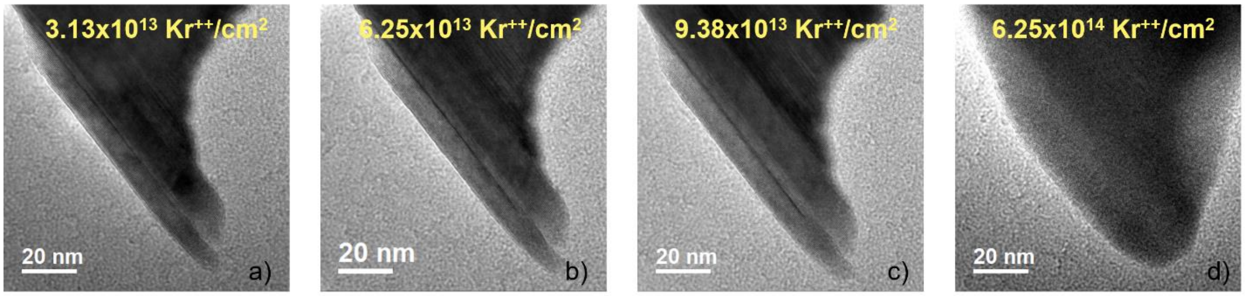

Supplementary Figure 8. TEM images of a particle of $Na_{2/3}Fe_{1/2}Mn_{1/2}O_2$ at the fluence of (a) $3.13x10^{13}$ Kr^{++}/cm^2, (b) $6.25x10^{13}$ Kr^{++}/cm^2, (c) $9.38x10^{13}$ Kr^{++}/cm^2, and (d) $6.25x10^{14}$ Kr^{++}/cm^2.

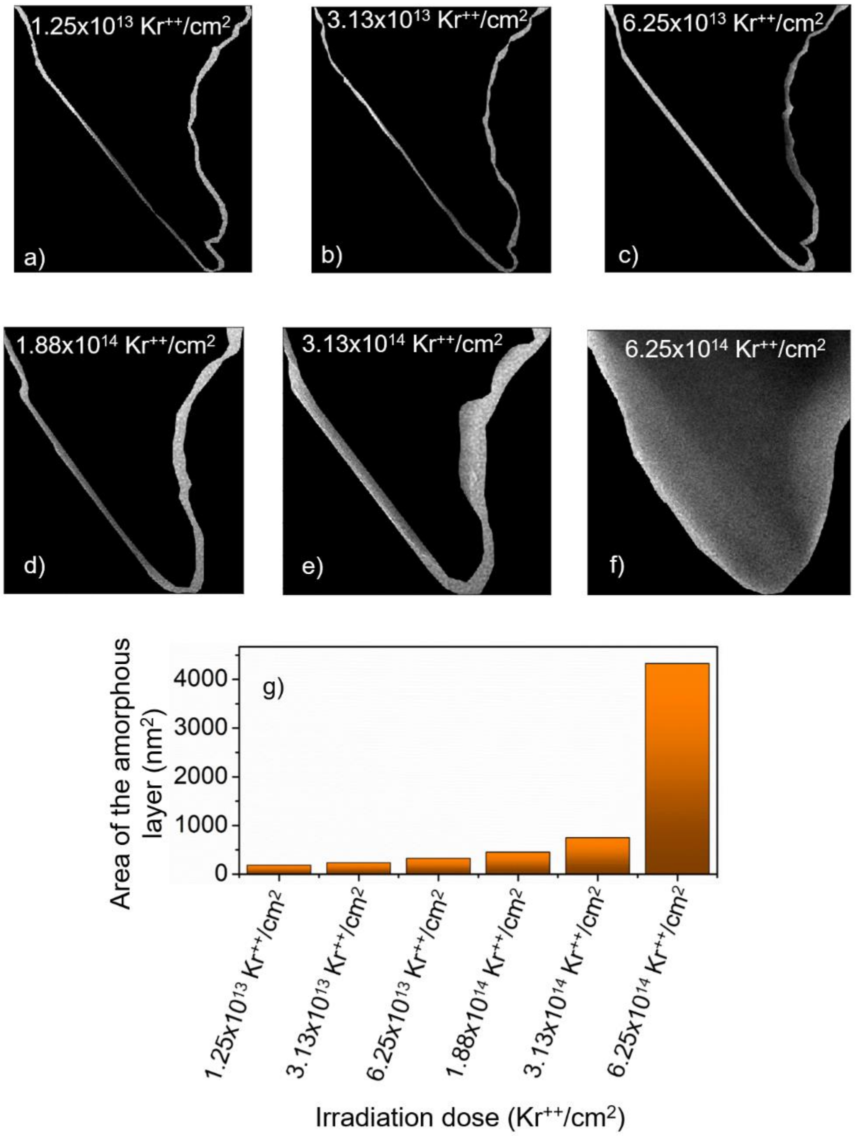

Supplementary Figure 9. Evolution of the amorphous layer of the irradiated $Na_{2/3}Fe_{1/2}Mn_{1/2}O_2$ particle at the fluence of (a) $1.25x10^{13}$ Kr^{++}/cm^2, (b) $3.13x10^{13}$ Kr^{++}/cm^2, (c) $6.25x10^{13}$ Kr^{++}/cm^2, (d) $1.88x10^{14}$ Kr^{++}/cm^2, (e) $3.13x10^{14}$ Kr^{++}/cm^2, and (f) $6.25x10^{14}$ Kr^{++}/cm^2. (g) Histogram showing the evolution of the amorphous layer at various fluences of Kr ion irradiation.

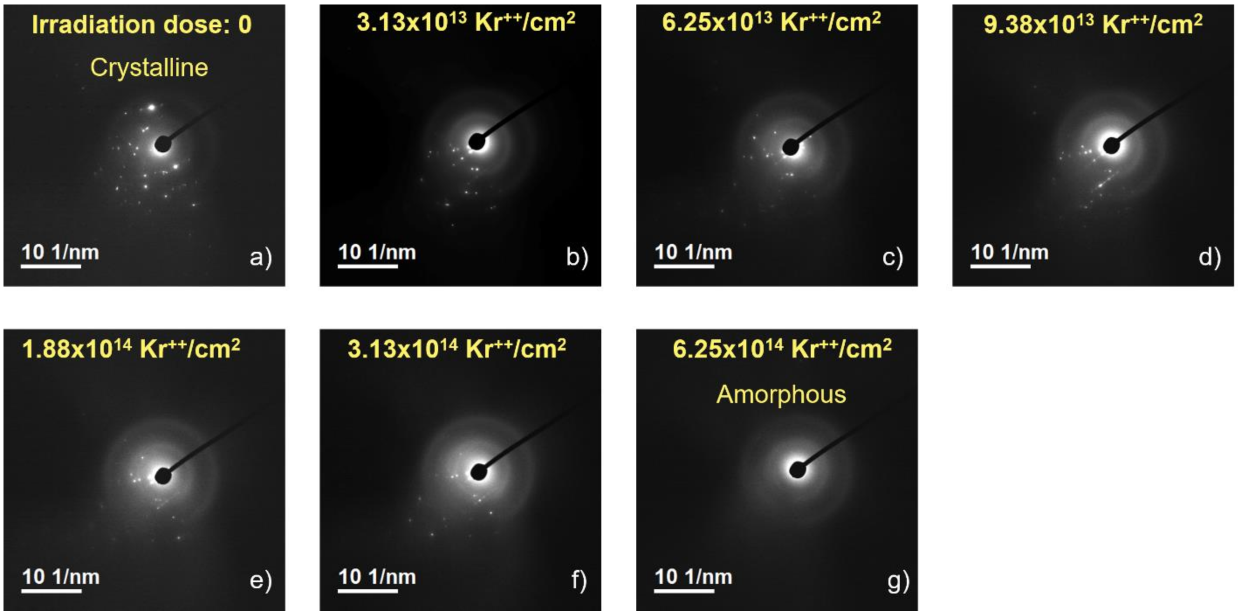

Supplementary Figure 10. Electron diffraction patterns of the $Na_{2/3}Fe_{1/2}Mn_{1/2}O_2$ particle for which the TEM images were taken at various fluence of Kr ion irradiation in Figure 3a-3c and Supplementary Figure 8, (a) before irradiation, (b) $3.13x10^{13}$ Kr^{++}/cm^2, (c) $6.25x10^{13}$ Kr^{++}/cm^2, (d) $9.38x10^{13}$ Kr^{++}/cm^2, (e) $1.88x10^{14}$ Kr^{++}/cm^2, (f) $3.13x10^{14}$ Kr^{++}/cm^2, and (g) $6.25x10^{14}$ Kr^{++}/cm^2.

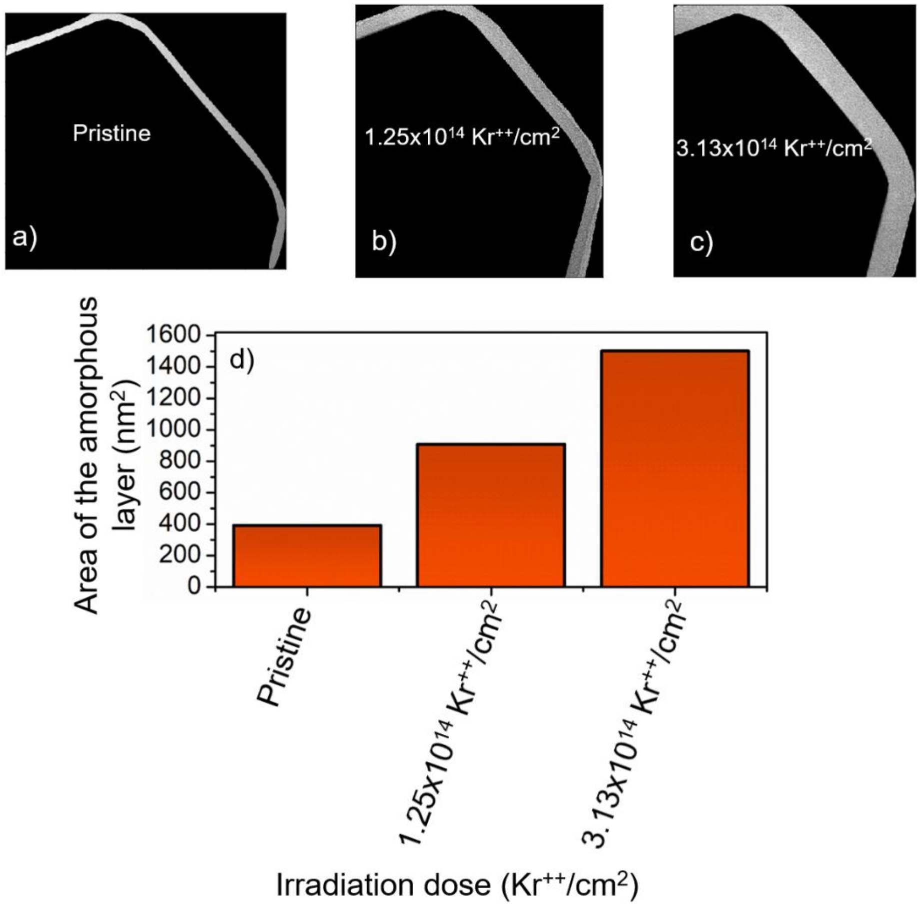

Supplementary Figure 11. Evolution of the amorphous layer on the irradiated $LiNiO_2$ particle (a) before irradiation and at the fluence of (b) $1.25x10^{14}$ Kr^{++}/cm^2, and (c) $3.13x10^{14}$ Kr^{++}/cm^2. (d) Histogram showing the evolution of the amorphous layer at various fluence of Kr ion irradiation.

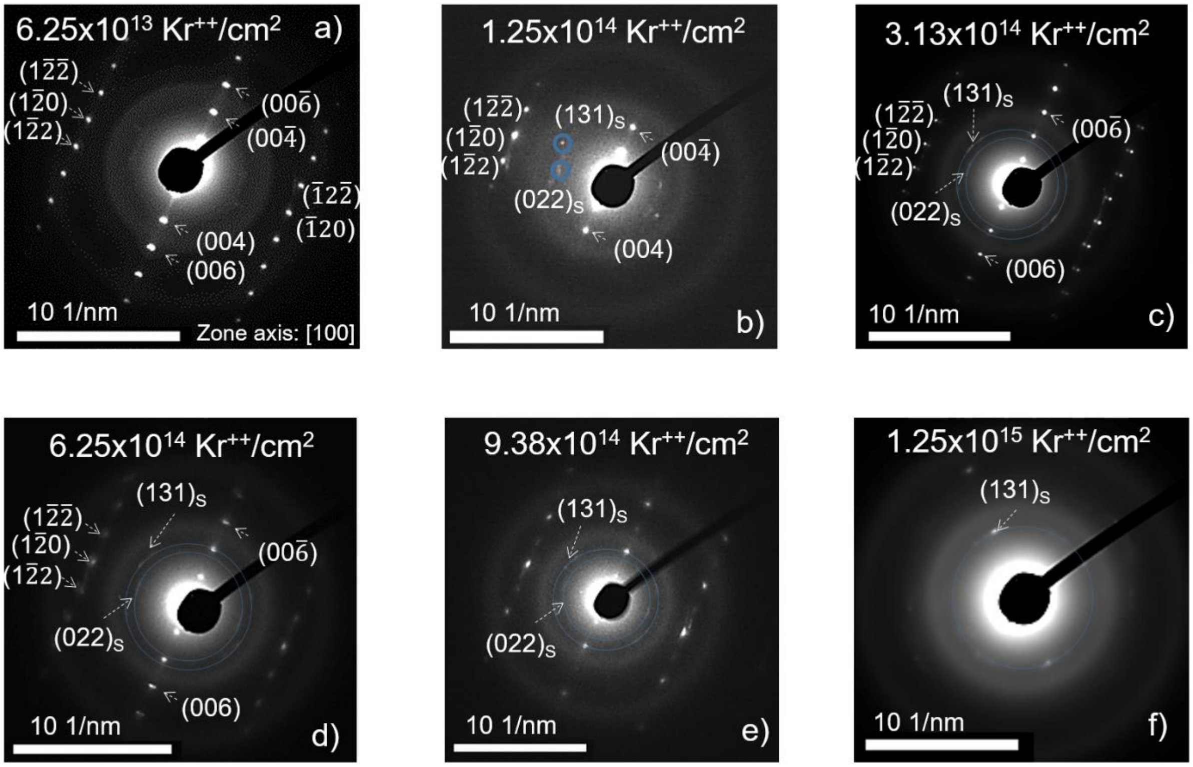

Supplementary Figure 12. Structural evolution at 200 °C of $Na_{2/3}Fe_{1/2}Mn_{1/2}O_2$ at various fluence of Kr ion irradiation. Electron diffraction pattern at the fluence of (a) 6.25x10^{13} Kr^{++}/cm^2, (b) 1.25x10^{14} Kr^{++}/cm^2, (c) 3.13x10^{14} Kr^{++}/cm^2, (d) 6.25x10^{14} Kr^{++}/cm^2, (e) 9.38x10^{14} Kr^{++}/cm^2, and (f) 1.25x10^{15} Kr^{++}/cm^2. The suffix 'S' in the images b, c, d, e, and f stands for the spinel phase.

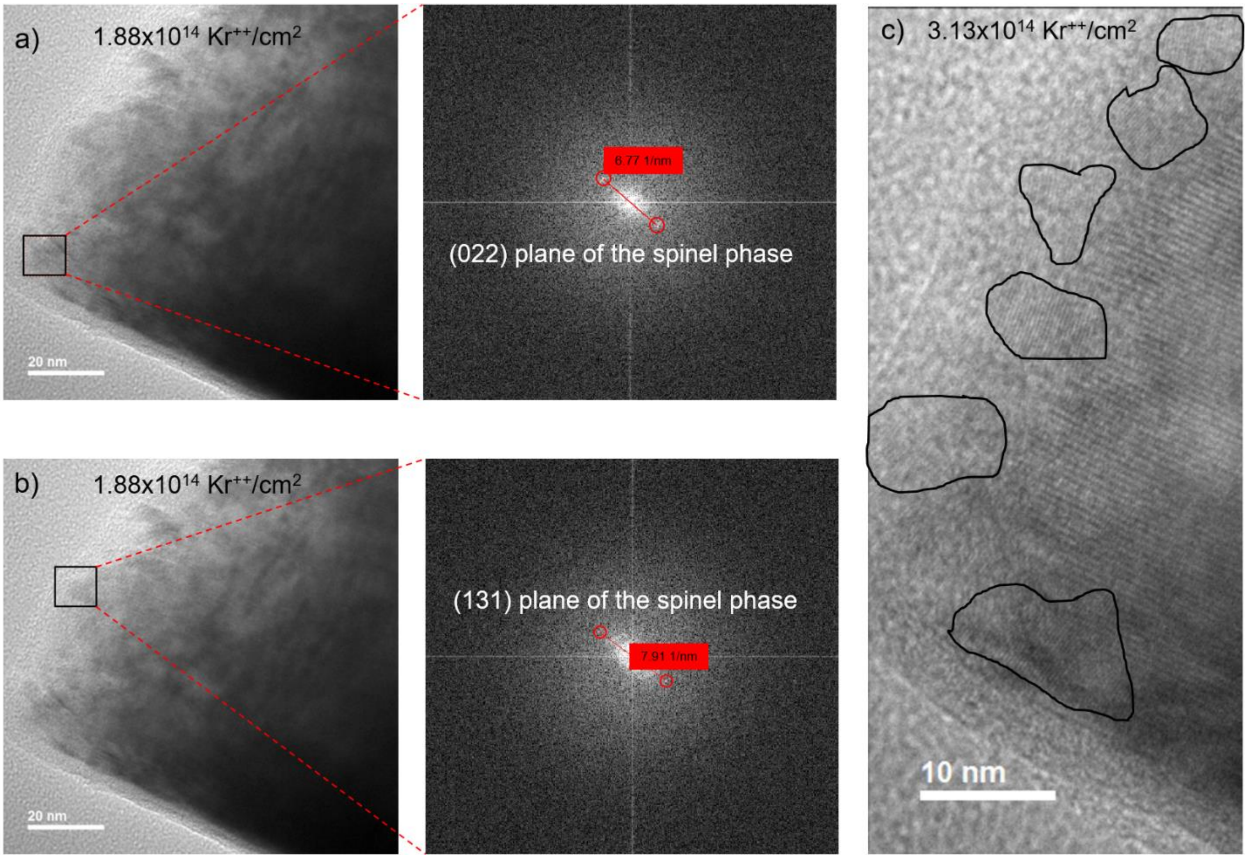

Supplementary Figure 13. (a-b) TEM image of a particle of $Na_{2/3}Fe_{1/2}Mn_{1/2}O_2$ irradiated at the fluence of $1.88x10^{14}$ Kr^{++}/cm^2 (left) and FFT of the boxed portion of the image (right). (c) TEM image of a particle of $Na_{2/3}Fe_{1/2}Mn_{1/2}O_2$ irradiated at the fluence of $3.13x10^{14}$ Kr^{++}/cm^2. The marked regions in the image shows the spinel phases in the particle.

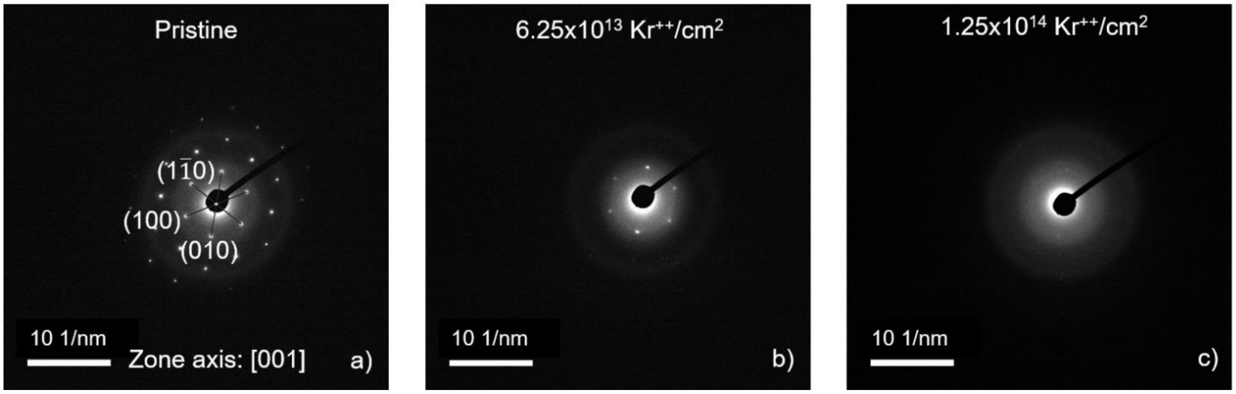

Supplementary Figure 14. Structural evolution of $Na_{2/3}Fe_{1/2}Mn_{1/2}O_2$ under Kr ion irradiation at -173 °C. Electron diffraction pattern (a) before irradiation and at the fluence of (b) $6.25x10^{13}$ Kr^{++}/cm^2 and (c) $1.25x10^{14}$ Kr^{++}/cm^2.

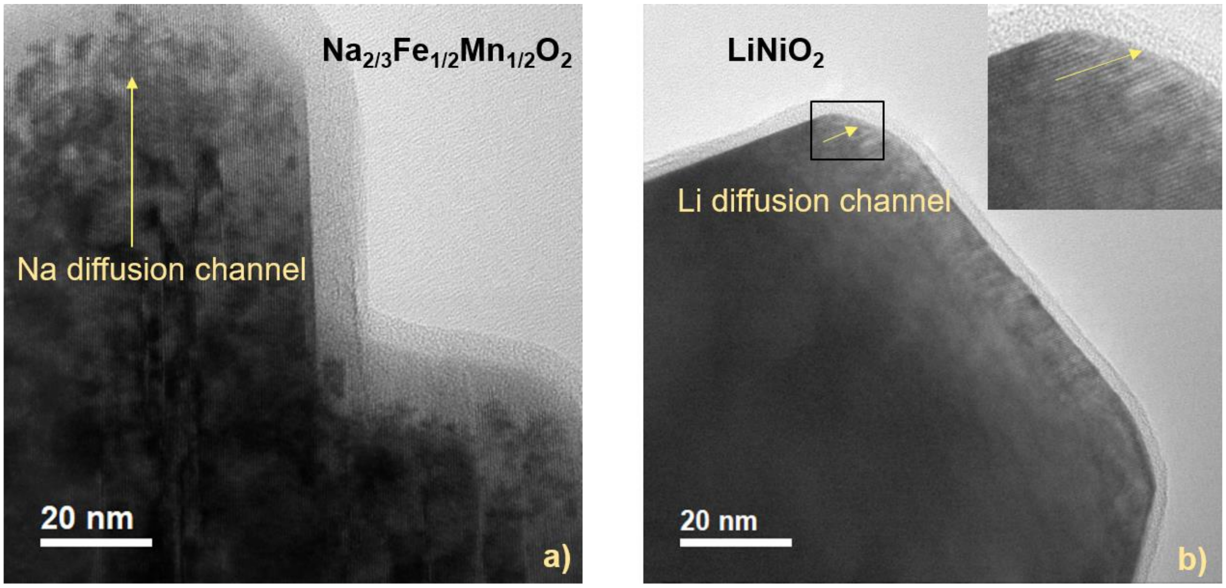

Supplementary Figure 15. The direction of the ion diffusion channels in (a) $Na_{2/3}Fe_{1/2}Mn_{1/2}O_2$, and (b) $LiNiO_2$.

Supplementary Discussion:

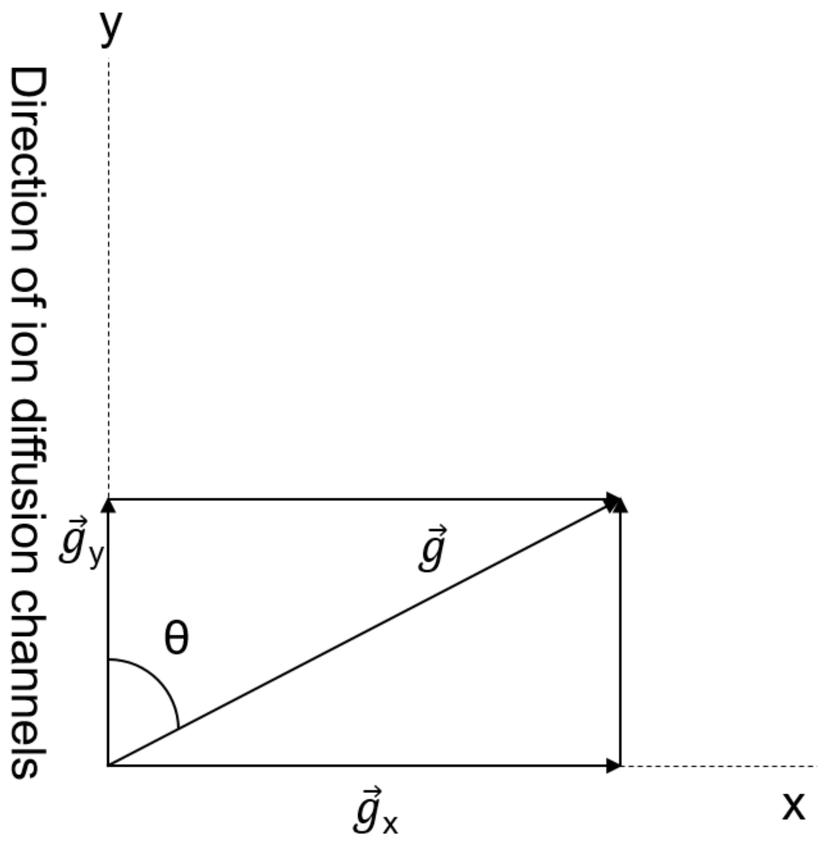

Supplementary Figure 16. Schematic representation of the calculation of the gradient vector.

$\vec{g}_x$ is the partial gradient vector along the x direction and $\vec{g}_y$ is the partial gradient vector along the y direction. The partial vectors show the brightness changes in the respective directions. The summation of the partial vectors gives the final gradient vector $\vec{g}$. The final vector shows the overall directional change in brightness, i.e. the overall direction in which the brightness of a pixel changes the most. The direction of the gradient vector is calculated with respect to the x direction by simple formula of the right angle triangle:

Direction of the gradient vector = arctan ($|\vec{g}_y|/|\vec{g}_x|$); where $|\vec{g}_x|$ and $|\vec{g}_y|$ are the magnitude of the partial vectors along the x and y direction.

The angle of the gradient vector is defined with respect to the Na/Li ion diffusion channel which is along the y direction. Hence the relationship between the direction of the gradient vector to that of the angle of the gradient vector is as follows:

Angle of the gradient vector (θ) = 90° - direction of the gradient vector

We will in principle observe five cases of angle of the gradient vector:

Case 1. Angle of the gradient vector is 0° with respect to the Na/Li ion diffusion channel (along the y direction)

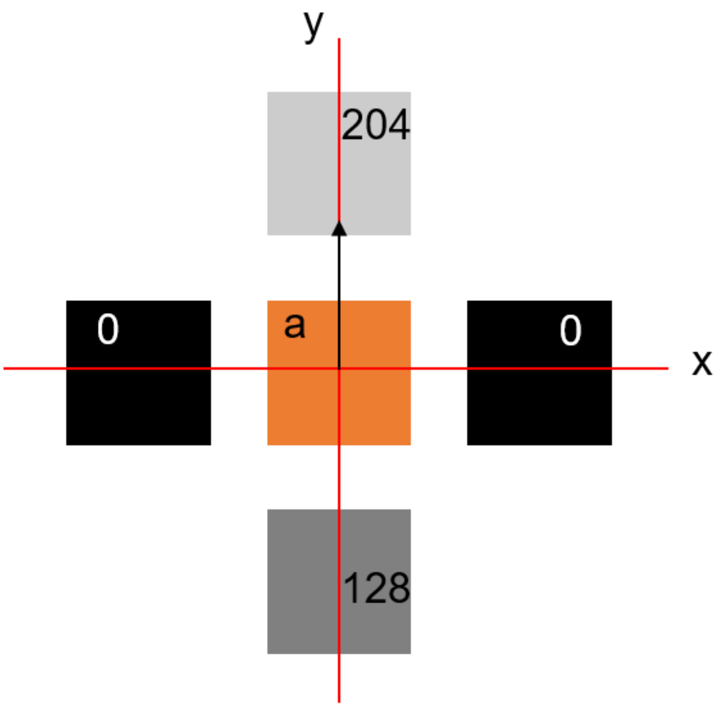

Supplementary Figure 17. An example of the case 1 where the gradient vector of a certain pixel 'a' is along the y direction (the Na/Li ion diffusion channel).

$|\vec{g}_y|$ = (204-128) = 76

$|\vec{g}_x|$ = (0-0) = 0

The direction of the gradient vector = arctan (76/0) = 90°

Angle of the gradient vector (θ) = 90°-90° = 0°

When there is no change in pixel value along the x direction, the gradient vector is located along the y direction. The angle of the gradient vector is then 0°. Now, if the pixels in the x direction represent defects (dark pixels), it means defects are distributed along the x direction.

Case 2. Angle of the gradient vector is 90° with respect to the Na/Li ion diffusion channel (y direction)

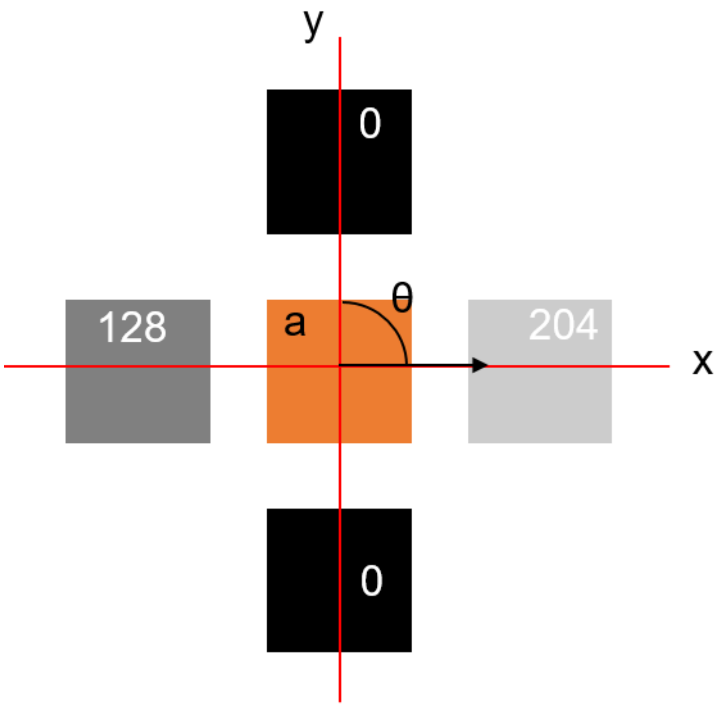

Supplementary Figure 18. An example of the case 2 where the gradient vector of a certain pixel 'a' is oriented perpendicular to the y direction (Na/Li ion diffusion channel).

$|\vec{g}_y|$ = (0-0) = 0

$|\vec{g}_x|$ = (204-128) = 76

The direction of the gradient vector = arctan (0/76) = 0°

Angle of the gradient vector (θ) = 90°-0° = 90°

When there is no change in pixel value along the y direction, the gradient vector is located along the x direction. The angle of the gradient vector is then 90° with respect to the Na/Li ion

diffusion channel. If the y direction (Na/Li ion diffusion channel) has dark pixels, it would mean that the defects are oriented along that direction.

Case 3. No preferential direction of defect orientation

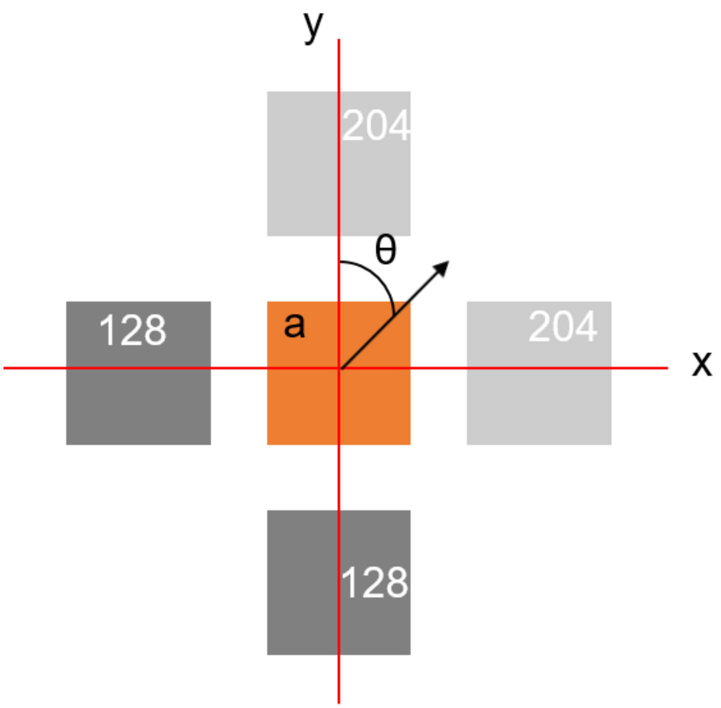

Supplementary Figure 19. An example of no preferential defect orientation. The gradient vector is right in the middle of the x direction and y direction.

$|\vec{g}_y| = (204\text{-}128) = 76$

$|\vec{g}_x| = (204\text{-}128) = 76$

The direction of the gradient vector = arctan (76/76) = 45°

Angle of the gradient vector (θ) = 90°-45° = 45°

In case of same gradient change in x and y directions, there is no preferential direction of defect evolution as the defect prefer both x and y directions.

The remaining two cases will be an intermediate between case 1 and case 2.

Case 4. Angle of gradient vector is less than 45° with respect to the Na/Li ion diffusion channel (y direction)

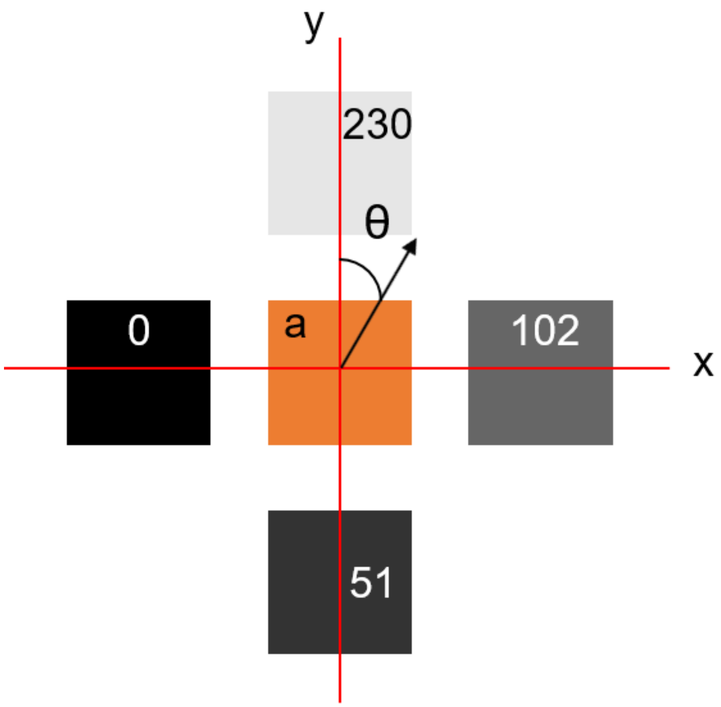

Supplementary Figure 20. An example of the gradient vector leaning more towards the y direction (the Na/Li ion diffusion channel) than towards the x direction.

$|\vec{g}_y| = (230\text{-}51) = 179$

$|\vec{g}_x| = (102\text{-}0) = 102$

The direction of the gradient vector = arctan (179/102) = 60.3°

Angle of the gradient vector (θ) = 90°-60.3° = 29.7°

In this case, there is more change in pixel value in the y direction than the x direction. This points towards defect distribution relatively more towards the x direction.

Case 5. Angle of gradient vector is more than 45° with respect to the Na/Li ion diffusion channel (y direction)

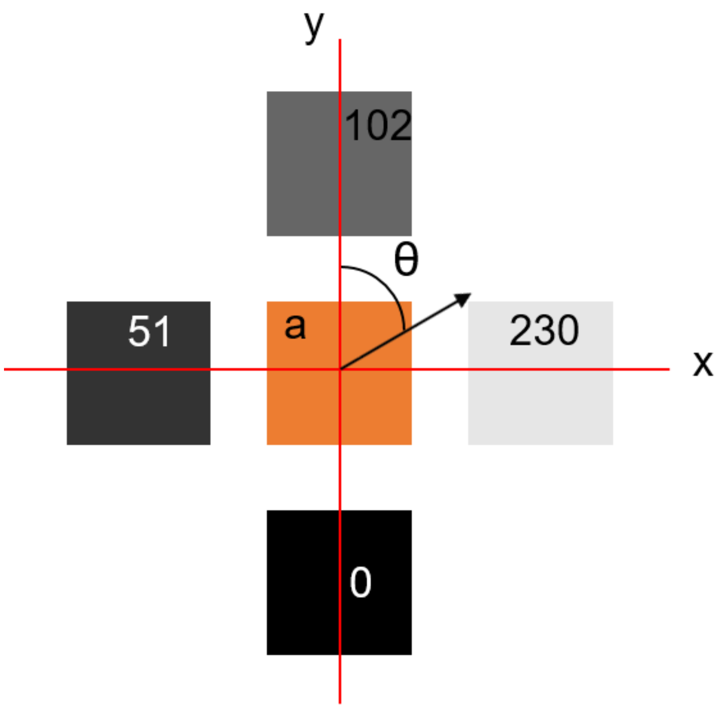

Supplementary Figure 21. An example of angle of gradient vector leaning more towards x direction compared to the y direction.

$|\vec{g}_y|$ = (102-0) = 102

$|\vec{g}_x|$ = (230-51) = 179

The direction of the gradient vector = arctan (102/179) = 29.7°

Angle of the gradient vector (θ) = 90°-29.7° = 60.3°

This case is exact opposite of the case 4. This would translate into less rapid change in pixel values along y direction (Na/Li ion diffusion channel). Now, if the less rapid change is for dark pixels, then the defects are oriented comparatively more towards the Na/Li ion diffusion channel (y direction) than the other direction (x direction).

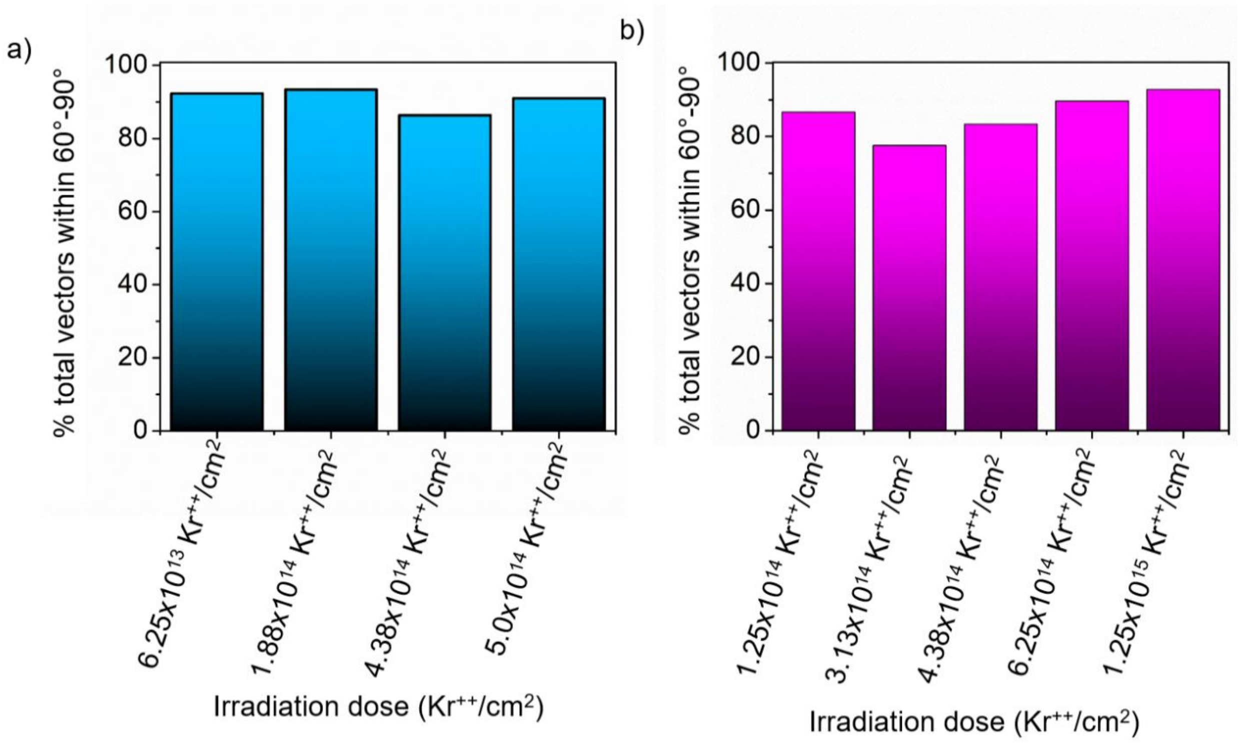

Supplementary Figure 22. Percentage of total vectors within the angle 60°-90° for (a) $Na_{2/3}Fe_{1/2}Mn_{1/2}O_2$ (from gradient vector computation in Figure 4b-4e), and (b) $LiNiO_2$ (from gradient vector computation in Figure 5a-5e).

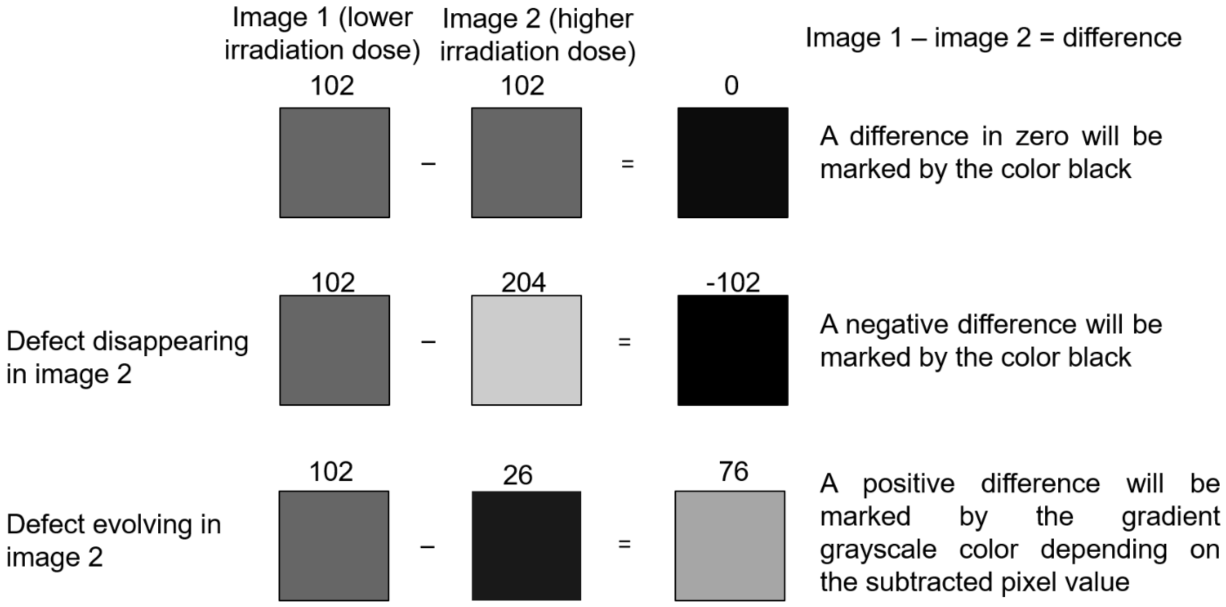

Supplementary Figure 23. Scheme explaining the subtraction of the images in Figure 4b-4e and Figure 5a-5e to understand the defect evolution.

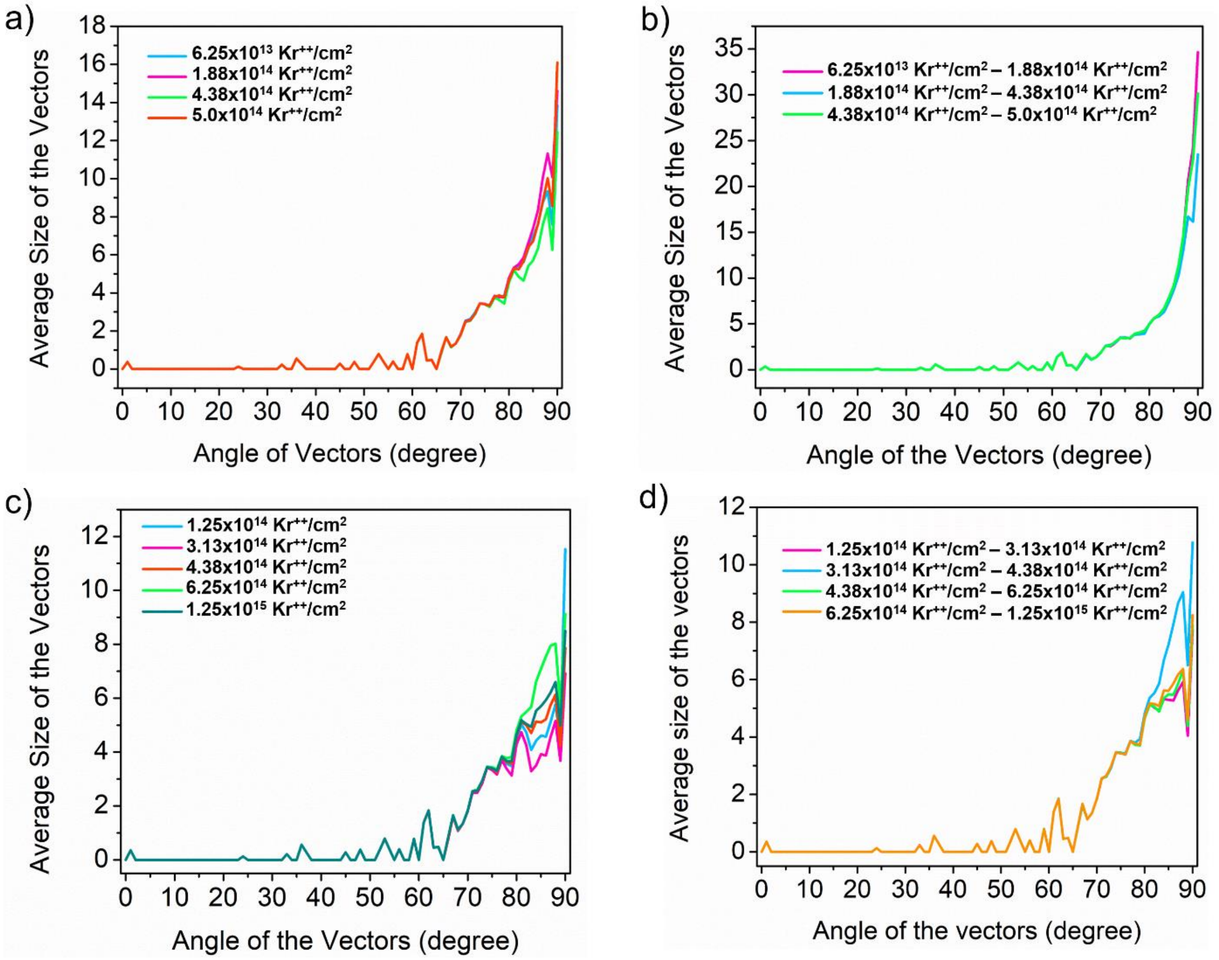

Supplementary Figure 24. Histogram of the average size of the vectors against the angle of the vectors. (a) Size of the vectors against the angle from the gradient computation on an irradiated particle of $Na_{3/2}Fe_{1/2}Mn_{1/2}O_2$ in Figure 4b-4e. (b) Size of the vectors against the angle of the vectors from the gradient computation on an irradiated particle of $Na_{2/3}Fe_{1/2}Mn_{1/2}O_2$ in Figure 4h-4j. (c) Size of the vectors against the angle of the vectors from the gradient computation on an irradiated particle of $LiNiO_2$ in Figure 5a-5e. (d) Size of the vectors against the angle of the vectors from the gradient computation on an irradiated particle of $LiNiO_2$ in Figure 5g-5j.

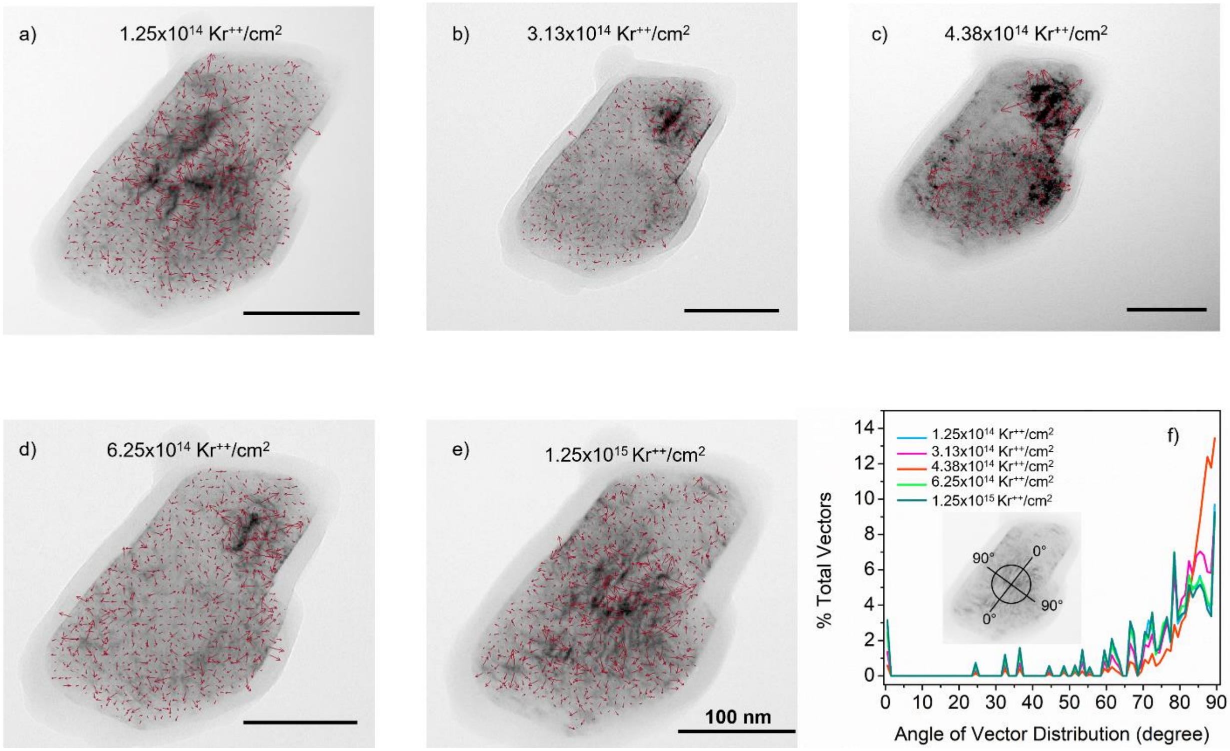

Supplementary Figure 25. Gradient vectors calculated and plotted on the charged $LiNiO_2$ particle at the fluence of (a) $1.25x10^{14}$ Kr^{++}/cm^2, (b) $3.13x10^{14}$ Kr^{++}/cm^2, (c) $4.38x10^{14}$ Kr^{++}/cm^2, (d) $6.25x10^{14}$ Kr^{++}/cm^2, and (e) $1.25x10^{15}$ Kr^{++}/cm^2. (f) Distribution of the gradient vectors against the angle of the gradient vectors of the image a to image e. The inset showed how the angle of the gradient vector was defined.

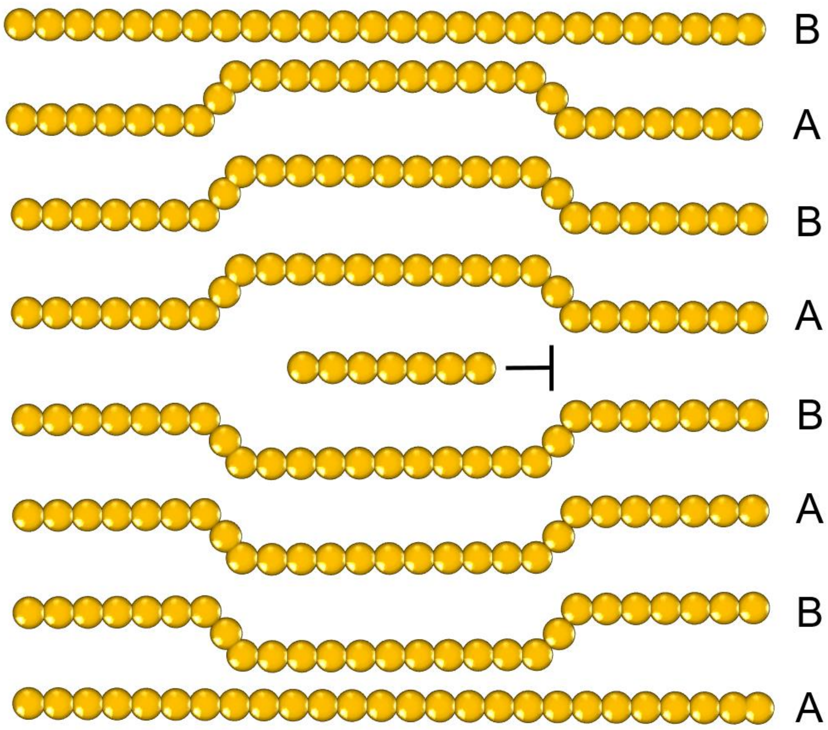

Supplementary Figure 26. Schematic illustration of interstitial type dislocation loop formation in irradiated materials. The formation of an extra plane will cause lattice distortion in the nearby planes.

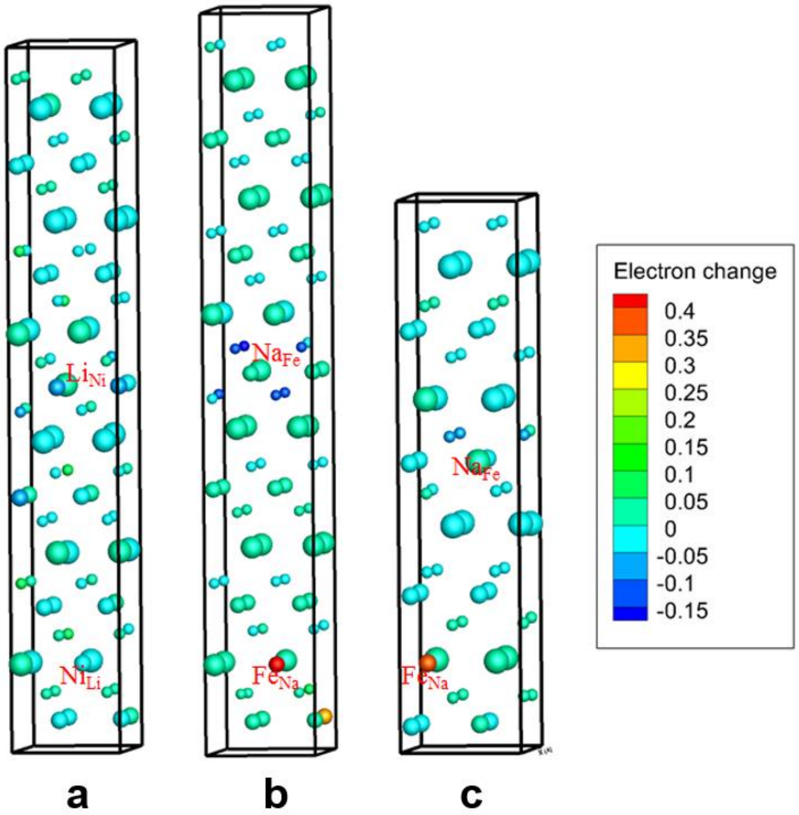

Supplementary Figure 27. Illustration of the charge transfer distribution due to the formation of antisite defects. Each atom is colored by the change of its valence electrons with respect to its counterpart in the perfect system. Large spheres represent Li or Na atoms, medium spheres represent Ni or Fe atoms, and small spheres represent O atoms. (a) O3-$LiNiO_2$. (b) O3-$NaFeO_2$. (c) P2-$NaFeO_2$.

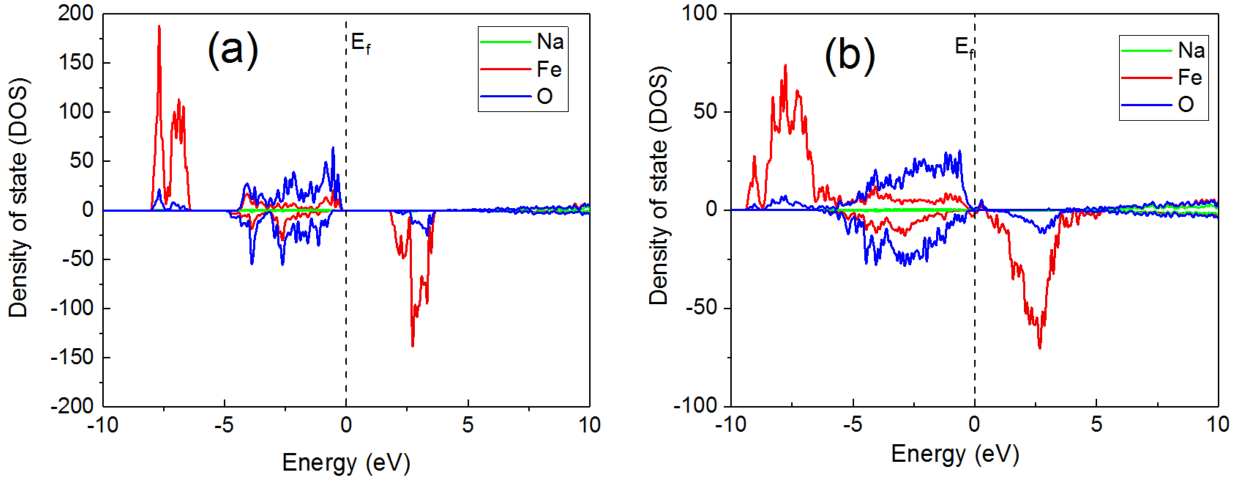

Supplementary Figure 28. Density of states (DOS) for (a) perfect O3-$NaFeO_2$ and (b) defective O3-$NaFeO_2$ containing an antisite pair. To check how the formation of antisite defects modifies the electronic structures of the battery materials, density of states (DOS) are calculated. In both plots, the Fermi level is shifted to zero. In the perfect O3-$NaFeO_2$ (Figure a), a bandgap can be clearly seen between the valance band maximum (VBM) and conduction band minimum (CBM). The asymmetry in the up-spin and down-spin channels indicates that the material also has some magnetism. When the system contains a pair of antisite defects (Figure b), interestingly the VBM and CBM connect to each other and the bandgap disappears. Similar behavior also can be found in P2-$NaFeO_2$ and O3-$LiNiO_2$. The result suggests that the defective system might have a metallic characteristic. However, the insulating to metallic transition may be an outcome of the high antisite defect concentration in DFT calculations. In our simulation, the concentration of antisite defects is about 4% (1/24) with respect to the total cation sites in O3-$NaFeO_2$. It is unclear if such a high antisite concentration is achievable in the irradiation experiments or at the total fluence of 6.25×10^{14} Kr^{++}/cm^2 utilized to irradiate Na-layered cathode at room temperature. Thus, further experimental investigation is needed to validate this theoretical prediction in the future.

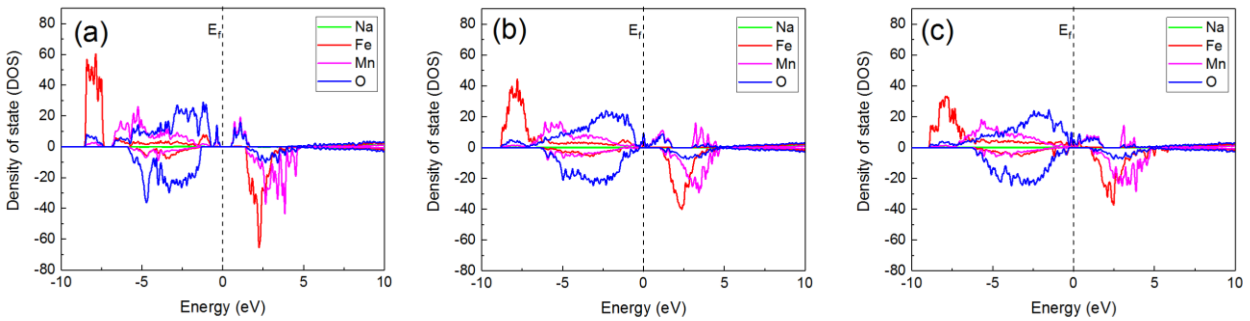

Supplementary Figure 29. Density of states (DOS) for (a) a pristine P2-$Na_{2/3}Fe_{1/2}Mn_{1/2}O_2$, (b) a defective system containing an Fe-Na antisite pair, and (c) a defective system containing a Mn-Na antisite pair. Note that we did not use any hybrid functional to tune the bandgap width because the study of electronic properties is beyond the scope of this work. Similar to Supplementary Figure 28, the bandgap vanishes in the two defective systems.
