# Supplementary material for: Defect and structural evolution under high-energy ion irradiation informs battery materials design for extreme environments
Source: Nat Commun. 2020 Sep 11;11:4548. doi: 10.1038/s41467-020-18345-4 (PMC7486889; doi:10.1038/s41467-020-18345-4)
Supplement: Supplementary file 2 — Peer Review [file 41467_2020_18345_MOESM2_ESM.pdf]

Reviewer #1 (Remarks to the Authors):

In this manuscript, Rahman and the co-authors have reported a investigation on the behaviour of defects and structure evolution in Na- and Li- layered oxides with 3d transition metals, which is conducted for the first time using in situ high-energy Kr ion irradiation with transmission electron microscopy. The obtained experimental and theoretical results on the defect cluster alignment and the resistance of materials to the high-energy beam irradiation could contribute a new insight in fundamental defect dynamics in layered oxide and good concept for designation of new cathode materials.

1. As the discussion of author, the preferential alignment of defect clusters is highly related to the accumulation to form the dislocation loops as illustrated in Figure S26. The expansion in dislocation loops should be demonstrated by an evident

2. In the discussion on effect of temperature, the author mentioned the formation of spinel phase in case of $Na_{2/3}Fe_{1/2}Mn_{1/2}O_2$ at 200 °C. Could the author give more discussion if the phase transition give any effects on enhancement in resistance of materials to beam irradiation at high temperature? Could the similar phenomenon be observed in $LiNiO_2$ case? A further explanation of the reason why high temperature could overcome the amorphization should be addressed

3. Why the Kr^{++} ion irradiation was used in this study? Do the authors consider about the size effect of this ion?

4. In the Bader charge analysis, the “delocalized” and “localized” term should be further described. The supporting viewpoint of this result contributing to the antisite defect formation should be pointed out. The authors also demonstrated that the formation of antisite defect give to the defective materials a more metallic behaviour. Could the author give further explanation on this effect? Could the case of localized or delocalized charge or mixed behaviour give any change in this effect?

5. For explanation of better charge transfer and low formation energy in antisite defect, the simulation of NaFeO2 and LiNiO2 was applied. The reason regarded to ionic radius differences in case of Mn also seem not to be mentioned. The differences in electronic configuration may also give a significant change in charge transfer of antisite defect. Could the author give any comment on this doubting?

6. In the discussion on loss of crystallinity, while the full amorphization of Na2/3Fe1/2Mn1/2¬O2 is shown clearly, the similar result of LiNiO2 is not presented. And the area of amorphous layer in LiNiO2 at ion beam dose of 3.13x1014 Kr++/cm2 is larger than that of Na2/3Fe1/2Mn1/2¬O2 which is conflicted to the conclusion of the author. The further evident and explanation should be added.

Reviewer #2 (Remarks to the Author):

The manuscript entitled “Defect and structural evolution under high energy ion irradiation informs battery materials design for extreme environments” reports in situ TEM characterization of defect evolution in high energy Kr ion irradiated Na- and Li-layered positive electrode materials (Na2/3Fe1/2Mn1/2O2 and O3-LiNiO2). Defect engineering is an emerging field in battery research. However, how defects influence the electrochemical and structural properties of battery

materials is largely unknown because the characterization of defects or defect evolution is very challenging. New advanced characterization techniques or analyses are desired to explore the role of defects. The responses of electrode materials under ion irradiation can be used as an analog to understand the defect evolution within such materials upon electrochemical cycling in battery systems. In this work, the authors applied an unique and comprehensive mathematical analysis on bright field TEM images of the layered electrode materials under Kr+ irradiation at different fluences. The statistical analysis was done through pixel by pixel gradient vector (due to brightness change in image) computation. This is the first time such analysis is applied to understand defect evolution in ion irradiated battery materials. The analysis suggests the preferential alignment of defect clusters along the transport ion diffusion channels. The results are new and significant. They help elucidate defect evolution in electrode materials under ion irradiation. Through in situ TEM, it was also found that LiNiO2 is more resistant to radiation damage. Such phenomenon is explained through theoretical analysis (DFT), which revealed that antisite defect formation energy is smaller in LiNiO2 because of the small difference in ionic radius between Li+ and Ni3+, compared to P2-Na2/3Fe1/2Mn1/2O2 which have larger difference in ionic radius between Na+ and transition metals. The results presented in this work will help to understand the defect evolution in solid state battery materials and therefore it is suggested for publication at Nat. Comm. with minor revision.

(1) The direction of the Kr+ ion beam should be labelled in Figure 3 in order to better understand the propagation of the defects.

(2) The authors are suggested to include a short discussion till the end to relate the responses of the layered electrode materials to ion irradiation and the corresponding defect evolution to their electrochemical performance such as cycling stability.

(3) The authors suggested “the possible formation of interstitial-type defect clusters…” what about the possibility of cation vacancies?

(4) The authors have done significant work in the past to study the effects of doping. Doping in electrode materials are known to improve structural stability. It would be interesting to know how doping will affect the responses of the layered materials under ion irradiation.

Reviewer #3 (Remarks to the Author):

This manuscript reports on defect evolution in layered electrode materials under high energy ion irradiation. Overall, the study is of high quality, although I'm not entirely convinced that a publication in nature communication is warranted. My objections are:

1. The connection to the electrochemical performance is vague. No doubt that many defects are formed during electrochemical cycling, however, the nature of the defects could be very different from that created by high energy ion radiation. How does the current study improve our understanding on defect dynamics associated with electrochemical cycling? how does the charge/discharge profile change after irradiation? The authors did mention that "the dynamics of defect evolution and structural transformations through electrochemical cycling shares similarities to that under the high energy ion irradiation". Unfortunately, no further explanation was given, and no reference was provided.

2. the authors argued that the current study informs battery design under extreme conditions. However, are battery electrodes subject to high-energy ion bombardment directly? If so, are the energy and fluence of Kr irradiation studied in this manuscript representative of the real service environment?

3. The authors reported total fluence, but I couldn't find any information regarding fluence rate, which is also an important parameter. Would different fluence rate make a difference?

4. The authors reported a negative antisite energy for $LiNiO_2$ system. Does that make sense?

Dear Reviewers,

Thank you very much for your time and effort in reviewing this manuscript. We appreciate the detailed and constructive comments. We have taken every comments into consideration and performed addition calculations and experiments. We believe that addressing these constructive feedbacks has enabled us to enhance the quality of the manuscript.

Reviewer #1 (Remarks to the Authors):

In this manuscript, Rahman and the co-authors have reported a investigation on the behaviour of defects and structure evolution in Na- and Li- layered oxides with 3d transition metals, **which is conducted for the first time using in situ high-energy Kr ion irradiation** with transmission electron microscopy. The obtained experimental and theoretical results on the defect cluster alignment and the resistance of materials to the high-energy beam irradiation **could contribute a new insight in fundamental defect dynamics in layered oxide and good concept for designation of new cathode materials.**

Response: Thank you very much for your valuable comments and for recognizing the importance of our work. We have taken all the comments into consideration and performed necessary analyses and changes to the manuscript. We believe the changes have enriched the manuscript further.

1. As the discussion of author, the preferential alignment of defect clusters is highly related to the accumulation to form the dislocation loops as illustrated in Figure S26. The expansion in dislocation loops should be demonstrated by an evident

Response: Thank you for your suggestion. The expansion of the defect clusters to dislocation loops is drawn from the conclusion of our study on the defect cluster distribution and propagation, which is based on statistical analysis of thousands of gradient vectors (please see the detailed discussion of Figure 4 and 5 and related text for more detail). The defect clusters in the materials studied in this work are monitored through bright field images at different Kr ion irradiation doses. The clusters are manifested as black spots in the images because they diffract more beam away from those regions. We believe that defect clusters further evolve into dislocation loops based on two key points. First is the large interlayer spacing between the two transition metal layers. The d spacing is around 5.58 Å for (002) planes and 4.75 Å for (003) planes of the P2 and O3 type layered oxides, respectively (see insets of Figure 1a and 1b). Dislocation loops are formed when the interstitial defects accumulate to form an extra plane, as schematically illustrated in Fig. S26. Such accumulation of the interstitials will require a large enough interlayer space which is available in the layered oxide materials studied in this work.

Second and the more obvious evidence is the preferential defect alignment determined from our defect evolution study. The accumulation of the interstitial defects in the interlayer space indicates that the dislocation loop will have a preferred direction of evolution along the ion diffusion channels (interlayer space) on the a-b plane of the material. Our experimental evidence is in accordance with the dislocation loop formation in other layered materials such as graphite [*J. Nucl. Mater*. **412**, 321–326 (2011)]. In graphite, the dislocation loops are formed parallel to the basal

planes. The defect clusters distribution and propagation (Figure 4g and 4k) conclusively show that the defect clusters indeed tend to align along the direction of the ion diffusion channel, parallel to the basal planes (a-b plane) when viewed from the [100] zone axis (see Figure 4 and 5 and the related text for more detail). Figure 4 is also copied below for your reference.

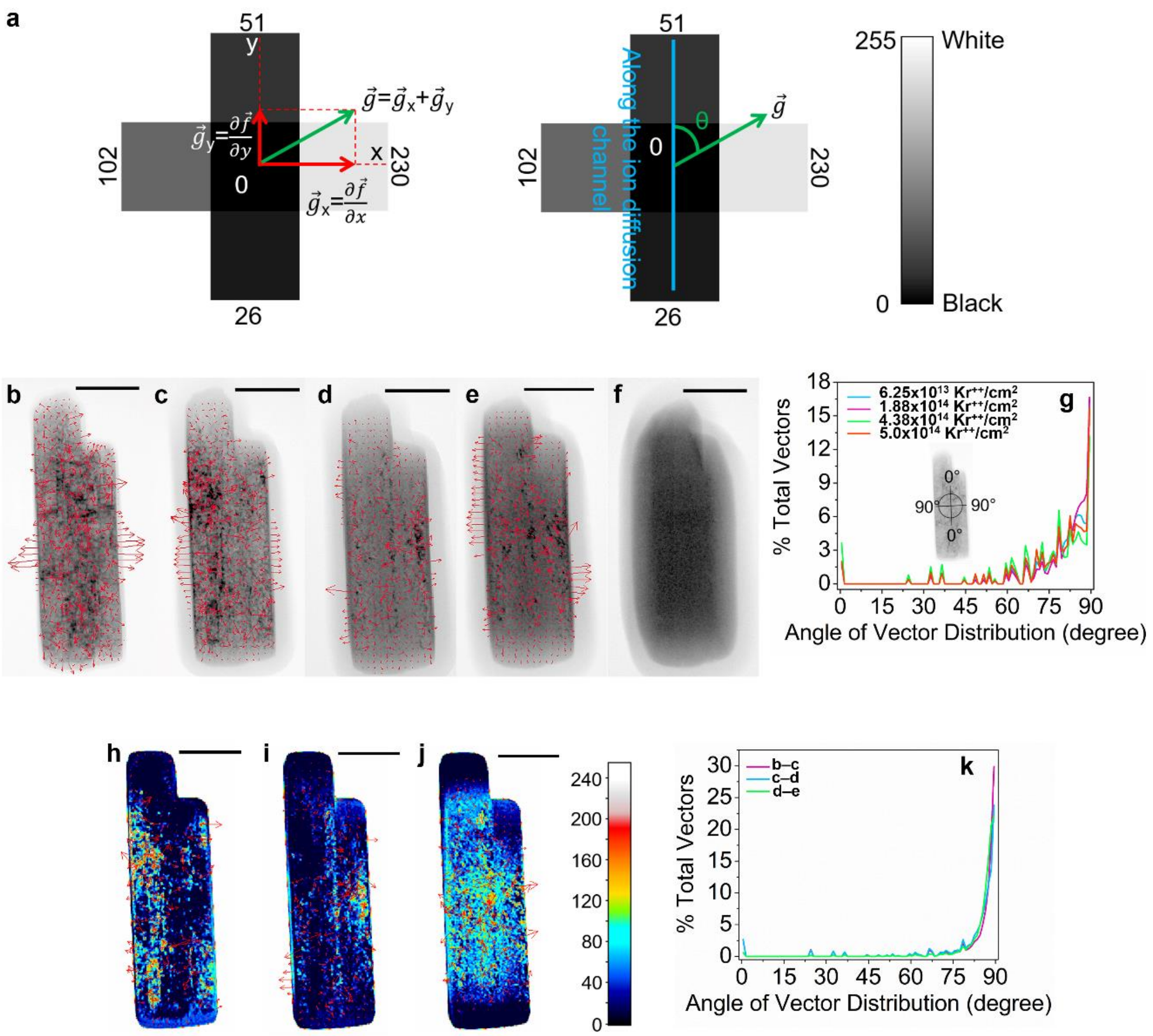

Figure 4. Quantitative analysis of the defect clusters distribution and evolution in $Na_{2/3}Fe_{1/2}Mn_{1/2}O_2$ particles irradiated by Kr ion at room temperature. Grayscale bright field 2-beam images are acquired to study the defect clusters distribution and evolution. (a) Scheme presenting the calculation of the gradient vector from a certain pixel of a bright field 2-beam image. The gradient vector points to the overall direction of the largest change in pixel value. Gradient vector calculated and superimposed on the bright field 2-beam image of a $Na_{2/3}Fe_{1/2}Mn_{1/2}O_2$ particle irradiated at the total fluence of (b) 6.25×10^{13} Kr^{++}/cm^2, (c) 1.88×10^{14} Kr^{++}/cm^2, (d) 4.38×10^{14} Kr^{++}/cm^2, and (e) 5.0×10^{14} Kr^{++}/cm^2. Bright field 2-beam image of a $Na_{2/3}Fe_{1/2}Mn_{1/2}O_2$ particle irradiated at the total fluence of (f) 6.25×10^{14} Kr^{++}/cm^2. The bright field images are taken

from the [100] zone axis. All the scale bars in the image b to f correspond to a length of 100 nm. (g) Distribution of the gradient vectors of image b to image e against the angle of the gradient vector. The inset shows the scheme of how the angle of the gradient vector is defined. Dynamic defect evolution in a $Na_{2/3}Fe_{1/2}Mn_{1/2}O_2$ particle with increasing fluence of Kr ion irradiation (Figure 4h-4j). The dynamic defect evolution is studied through the subtraction of the image acquired at higher irradiation dose from that of the lower irradiation dose (e.g. image c subtracted from image b). Defect evolution from (h) image b to image c, (i) image c to image d, and (j) image d to image e. All the scale bars from image h to image j correspond to a length of 100 nm. The color bar shows the corresponding values of the subtracted pixels after the subtracted grayscale image is converted to an RGB image. (k) Distribution of the gradient vectors of image h to image j against the angle of the gradient vector.

We explained both the aspects in the main text and copied below for your reference:

Page 20. This similar trend of preferential defect evolution in both layered materials points to the possible formation of interstitial-type defect clusters and potentially dislocation loops that are parallel to the Na ion or Li ion layers. The reason may be that in each material the interlayer space between two transition metal layers is large (Figures 1a and 1b). The large space provides free volume to accommodate the radiation-induced interstitial atoms. When interstitials accumulate in the interlayer space, they can form interstitial-type clusters or even an extra plane (dislocation loop) (see schematic in Figure S26). This mechanism is similar to the dislocation loop formation mechanisms in some other layered materials such as graphite under irradiation.[65,66] In graphite, accumulation of interstitials in-between basal planes (graphene layers) can form prismatic dislocation loops that are parallel to the basal planes, leading to lattice expansion in *c* direction and contraction in *a* direction.[65,66] The defect clusters or loops can cause lattice distortion[67] which will cause different contrast in the bright field images. Therefore, we believe that the large interlayer space in the layered oxide cathodes provides the needed free volume for the growth of the defect clusters or dislocation loops along the Na/Li ion diffusion channel. Furthermore, our conclusion is consistent with the experimental observation of edge dislocations in alkali-ion layered oxides.[68,69]

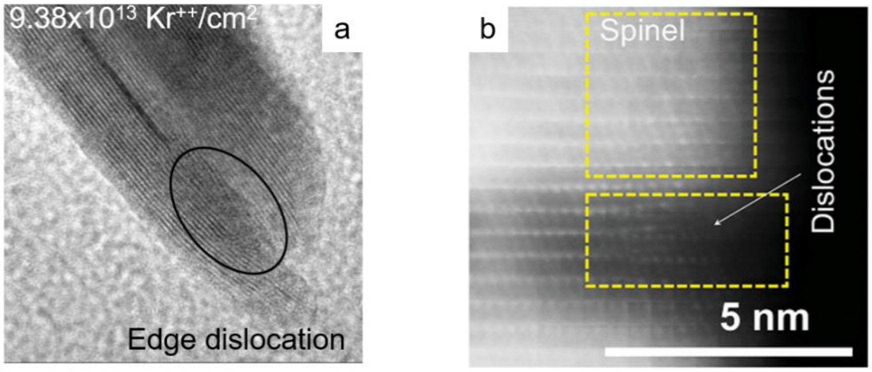

Figure R1. (a) Edge dislocation observed in Kr ion irradiated $Na_{2/3}Fe_{1/2}Mn_{1/2}O_2$ particle. (b) Dislocation observed in the particle of O3-$NaNi_{1/3}Fe_{1/3}Mn_{1/3}O_2$ after one cycle. Adapted with permission from reference 1.

Our conclusion of defect distribution is also consistent with edge dislocations observed in layered cathodes. Dislocations can be induced by ion irradiation (Figure R1a) or by electrochemical cycling (Figure R1b) and cause lattice distortion in the direction of the ion diffusion channels.

Furthermore, the ion irradiation dose utilized in this study are reported to form dislocation loops in many complex oxides [*Philos. Mag.* **93**, 4569–4581 (2013); *Nucl. Instruments Methods Phys. Res. Sect. B Beam Interact. with Mater. Atoms* **141**, 737–746 (1998); *J. Nucl. Mater.* **191–194**, 645–649 (1992); *Science* **318**, 923–924 (2007); *J. Nucl. Mater.* **443**, 71–77 (2013)] which further supports the claim of possible dislocation loops formation in our material.

2. In the discussion on effect of temperature, the author mentioned the formation of spinel phase in case of $Na_{2/3}Fe_{1/2}Mn_{1/2}O_2$ at 200 °C. Could the author give more discussion if the phase transition give any effects on enhancement in resistance of materials to beam irradiation at high temperature? Could the similar phenomenon be observed in $LiNiO_2$ case? A further explanation of the reason why high temperature could overcome the amorphization should be addressed

Response: Thanks to the reviewer for the insightful comment. Ceramic materials which are not resistant to high energy ion irradiation undergoes a transformation from a crystalline to an amorphous structure [*Nat. Mater.* **6**, 217–223 (2007)]. However, crystalline materials which are resistant to amorphization instead may undergo a phase transformation to an intermediate structure. For example, zirconate pyrochlores (e.g. $Gd_2Zr_2O_7$) are regarded as irradiation resistant because they undergo a phase transformation to a fluorite type structure rather than complete amorphization, when exposed to high energy ion irradiation [*Phys. Rev. B - Condens. Matter Mater. Phys.* **66**, 541081–541085 (2002); *Nuclear Instruments and Methods in Physics Research, Section B: Beam Interactions with Materials and Atoms* **218**, 236–243 (North-Holland, 2004)]. Resistance to amorphization also increases with an elevation of temperature because of the enhanced defect annihilation through the recombination of the point defects such as vacancies and interstitials [*Acta Mater.* **105**, 130–146 (2016); *Nucl. Instruments Methods Phys. Res. Sect. B Beam Interact. with Mater. Atoms* **405**, 15–21 (2017)]. At high temperatures, diffusion of point defects is fast so that interstitial-vacancy recombination becomes more effective. For such an enhanced defect recovery process at high temperature, the stability of Na- and Li-layered cathodes should enhance at high temperature. For example, our study shows that the resistance to amorphization of $Na_{2/3}Fe_{1/2}Mn_{1/2}O_2$ is enhanced at 200 °C in comparison to room temperature irradiation. Enhanced defect annihilation at high temperature should enhance the resistance to loss of crystallinity of $LiNiO_2$ as well. Since the irradiation induced structural transformations in layered oxide cathodes has remained largely unexplored, it was rather unknown until now how these materials would transform when exposed to high energy irradiation at a broad range of temperature. Our study shows that layered oxides such as $Na_{2/3}Fe_{1/2}Mn_{1/2}O_2$ transforms to a spinel phase instead of full amorphization at high temperature. This implies that some layered oxide materials can undergo phase transformation to a different crystalline phase at high temperature, similar to irradiation resistant pyrochlores. We have added the following statements in the “Conclusions and discussion” section to provide more insights to the reader:

Page 32. Like in many other oxide ceramics,[35] high temperature can lessen the severity of structural transformations of Na-layered oxide by accelerating the annihilation of radiation induced defects through the recombination of vacancies and interstitials.[82] Enhanced defect annihilation at high temperature should enhance the structural stability of Li-layered oxide as well. Instead of a direct crystalline to amorphous transformation, our study shows that Na-layered oxide undergoes a phase transformation to a spinel type structure at high temperature. Some irradiation resistant pyrochlores e.g. $Gd_2Zr_2O_7$ also undergoes a phase transformation to a fluorite type structure.[25] Such phase transformation is indicative of an intermediate phase formation rather than full disordering to an amorphous phase.

3. Why the Kr^{++} ion irradiation was used in this study? Do the authors consider about the size effect of this ion?

Response: Neutron irradiation in nuclear power industries and high energy solar flare particles or galactic cosmic radiation in outer space can cause extreme materials damage on prolonged exposure. Layered oxide battery materials working in these extreme environments are expected to undergo damage over time, similar to other complex oxides such as pyrochlores and fluorites [*Nat. Mater.* **6**, 217–223 (2007)]. Thus, in order to build a knowledge base for designing stable layered oxide cathodes under extreme environments, it is important to simulate the transformation of these materials over the entire service life within the practical time frame of laboratory experiments. High energy ion irradiation such as Kr ion irradiation can induce observable materials damage within a short period of time, which enables studying the transformation of materials *in situ* [*J. Nucl. Mater.* **37**, 1–12 (1970); *J. Nucl. Mater.* **216**, 78–96 (1994); *Scr. Mater.* **88**, 33–36 (2014)]. Besides, Kr ion irradiation can produce similar cascade damage profile to that of the neutron irradiation in a nuclear reactor, making the transformations of the materials reliable to the actual service environment [*Journal of Applied Physics* **107**, 071301 (2010)]. Hence, Kr ion irradiation was chosen for this study. Of course, other irradiation particles such as proton may also be used to produce radiation damage. We have added the following statement in the main text to reflect on this:

Page 5. Kr ion irradiation can induce observable damage within a short period of time.[46] The cascade damage profile produced by Kr ion irradiation is similar to neutron irradiation in a nuclear reactor.[45] Hence, efficient mirroring of defect and structural evolution throughout the actual service life in extreme environments is possible within the timescale of laboratory experiment.

As for the size effect of the Kr ion, the primary parameters controlling the damage of a material by a colliding ion are the mass and energy of the ion [*Reports Prog. Phys.* **18**, 1 (1955)]. The colliding ion transfers part of its kinetic energy to the target atom of a material called the primary knock-on atom (PKA) through elastic collision [*Philos. Mag.* **92**, 1469–1498 (2012)]. The PKA can have sufficient kinetic energy to displace other lattice atoms from their perfect lattice sites and form a cascade of point defects. Increasing the mass of the bombarding ions enhances the cross section of the elastic scattering, causing large number of point defects within the cascades [*Sci. Rep.* **7**, 1–11 (2017)]. For this reason, ions with light mass produce small cascades, and heavier ions create cascades dense with point defects. This helps to create observable materials damage

with high mass ions. As mentioned earlier, Kr ions can produce a cascade damage profile similar to neutron irradiation in nuclear reactors which is why Kr ion irradiation was chosen to mirror the materials damage in extreme environments. We should mention that this work is meant to kick start our project of developing stable battery materials for extreme environment. Beyond the current manuscript, we will take the reviewer's comment into account and potentially include different ions into our future studies. In a separate project, we have performed irradiation on electrode level with Ne ion. For the reviewing purpose, we have included the electrochemical performance of Ne ion irradiated $Na_{2/3}Fe_{1/2}Mn_{1/2}O_2$ electrode at two different total fluence (Figure R2). The degradation of the electrochemical performance (Figure R2a-d) and redox feature evolution (Figure R2e-f) on ion irradiation are apparent from the data. A drop of capacity retention from 77.5% to 70.1% (Figure R2c) is observed when the irradiation dose is increased. Voltage fades comparatively faster as well at higher irradiation dose (Figure R2d). We are performing detailed characterization to couple defect evolution and electrochemical performance. Along with the work reported in this manuscript, we believe we will be able to deliver concrete design principle and mechanistic understanding of the electrochemical performance of battery electrodes in extreme environments. We do not want to include the preliminary data in the present work because 1) the irradiation conditions are different, and 2) the focus on the present work is to understand the defect and structural evolution of layered cathode materials in general rather than the electrochemical performance.

Figure R2. Electrochemical performance of Ne ion irradiated $Na_{2/3}Fe_{1/2}Mn_{1/2}O_2$. Galvanostatic charge-discharge curves of the cell containing $Na_{2/3}Fe_{1/2}Mn_{1/2}O_2$ irradiated at a total fluence of (a) $2.42x10^{14}$ Ne^{++}/cm^2, and (b) $7.25x10^{14}$ Ne^{++}/cm^2 at C/5 rate for up to 20 cycles. (c) % capacity retention of $Na_{2/3}Fe_{1/2}Mn_{1/2}O_2$ irradiated at a total fluence of $2.42x10^{14}$ Kr^{++}/cm^2 and $7.25x10^{14}$ Kr^{++}/cm^2. (d) Average voltage vs cycle number of $Na_{2/3}Fe_{1/2}Mn_{1/2}O_2$ irradiated at a total fluence of $2.42x10^{14}$ Kr^{++}/cm^2 and $7.25x10^{14}$ Kr^{++}/cm^2. (e) Cyclic voltammetry curves of pristine $Na_{2/3}Fe_{1/2}Mn_{1/2}O_2$ at a scan rate of 0.15 mV/s. (f) Cyclic voltammetry curves of Ne ion irradiated ($2.42x10^{14}$ Ne ion/cm^2) $Na_{2/3}Fe_{1/2}Mn_{1/2}O_2$ at a scan rate of 0.15 mV/s.

4. In the Bader charge analysis, the "delocalized" and "localized" term should be further described. The supporting viewpoint of this result contributing to the antisite defect formation should be pointed out. The authors also demonstrated that the formation of antisite defect give to the defective materials a more metallic behaviour. Could the author give further explanation on this effect? Could the case of localized or delocalized charge or mixed behaviour give any change in this effect?

Response: According to the suggestion of the reviewer, we have added the definition of the terms "delocalized" and "localized" charge transfer in the main text.

Page 28. Here localized charge transfer means that the charge transfer is mainly concentrated at the antisite defect itself or its nearest neighbors; delocalized charge transfer means that the charge transfer spreads beyond this range.

Note that in the revised manuscript, we have also calculated the antisite formation energies for three Fe-Na and three Mn-Na antisite pairs in P2-$Na_{2/3}Fe_{1/2}Mn_{1/2}O_2$ directly (see main text for details). The ionic radii are nearly identical for Fe^{3+}/Mn^{3+}, and for Fe^{4+}/Mn^{4+}, which are all much

smaller than Na^+. Their antisite pair formation energies are much higher than that of Li-Ni in O3-$LiNiO_2$, which is fully consistent with our original claim. Charge transfer takes place on or near the antisite defect in a material. Hence, it is important to understand if there is any correlation between the charge transfer and the antisite formation energy. In addition to the charge transfer analyses in O3-$LiNiO_2$, O3-$NaFeO_2$, and P2-$NaFeO_2$, we have also performed similar charge transfer analysis for the additional six antisite pairs in $Na_{2/3}Fe_{1/2}Mn_{1/2}O_2$ (see Figure 7 and related text for more details) and found that the details of charge transfer depend on the specific materials system and local environment of the antisite defects. There is no clear correlation between the charge transfer behavior and the antisite defect formation energy. Therefore, we conclude that the main factor for affecting the TM-alkali antisite formation energy is ionic radius difference between them. We have added necessary explanation in the main text to explain this and copied below for your reference:

Page 29. The above analysis shows that the detailed charge transfer/redistribution mechanism is material specific. We have not observed a clear correlation between the detailed charge transfer mechanism and antisite formation energy. If other electronic configurations are used in our DFT modeling, the details of the charge transfer process may change somewhat. However, the trend of the antisite formation energy should not change significantly because the difference in ionic radius between TM and alkali cations is the key factor for determining the antisite defect formation energy.

For the reviewer's convenience, we have copied below our new Figure 7 and related explanation about the charge transfer mechanism in P2-$Na_{2/3}Fe_{1/2}Mn_{1/2}O_2$:

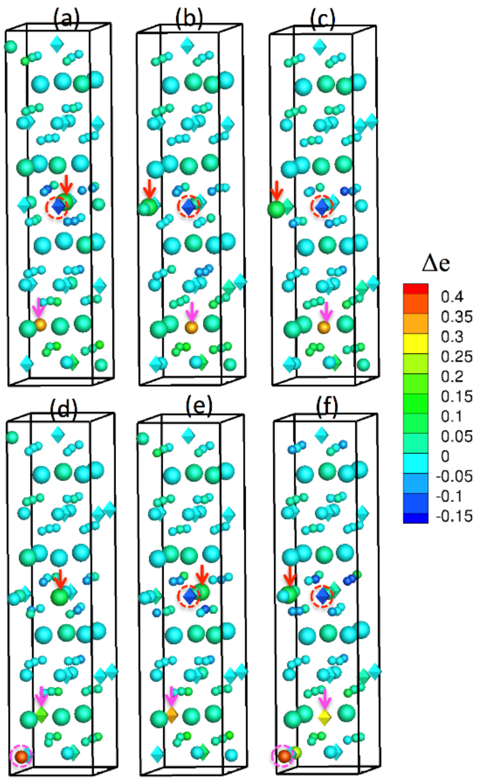

Figure 7. Illustration of the charge transfer distribution due to the formation of antisite defects in P2-$Na_{2/3}Fe_{1/2}Mn_{1/2}O_2$. Each atom is colored by the change of its valence electrons with

respect to its counterpart in the pristine system. Red and magenta arrows indicate the Na_{TM} and TM_{Na} antisite defects, respectively. Red dashed circles indicate a significant loss of electrons of some nearby Mn cations. Magenta dashed circles indicate a large gain of electrons of some nearby Fe cations. Large spheres: Na; Medium spheres: Fe; Medium diamonds: Mn; Small spheres: O. The antisite defect pairs are: (a) Fe_1 – Na_1. (b) Fe_2 – Na_2. (c) Fe_3 – Na_2. (d) Mn_1 – Na_1. (e) Mn_2 – Na_1. (f) Mn_3 – Na_2.

Page 28-30. The charge transfer in P2-$Na_{2/3}Fe_{1/2}Mn_{1/2}O_2$ is more complex, as shown in Figure 7. For the systems containing an Fe-Na antisite pair (Figs. 7a-7c), some nearby oxygen anions around the Na_{Fe} (at the middle of each figure in the vertical direction) lose electrons. Interestingly, one nearby Mn cation also loses some electrons, as indicated by the red dashed circle in each figure. This suggests that the oxidation state of the nearby Mn cation may increase to accommodate the charge difference between Na^+ and Fe^{3+}. At the Fe_{Na} antisite (near the bottom of each figure), the Fe_{Na} antisite defect gains some electrons, suggesting the oxidation state of Fe at the antisite may decrease. For the systems containing an Mn-Na antisite pair (Figs. 7d-7f), oxygen anions behave similarly as the cases with a Fe-Na antisite pair. Near the Na_{Mn} antisite (at the middle of each figure), a nearby Mn also tends to lose electrons, except in Fig. 7d. At the Mn_{Na} antisite (near the bottom of each figure), the Mn_{Na} antisite defect gains some electrons, indicating the Mn may lower the oxidation state. In two cases (bottom of Figs. 7d and 7f), a nearby Fe also gains some electrons. Overall, it seems that the oxidation state of Mn can either increase or decrease to accommodate antisite defects; while the oxidation state of Fe always tends to decrease. The different charge transfer behavior between Fe and Mn cations may shed a light on the experimental observation that Fe^{4+} is more difficult to form than Mn^{4+} in P2-$Na_{2/3}Fe_{1/2}Mn_{1/2}O_2$ during charging.[77] The above analysis shows that the detailed charge transfer/redistribution mechanism is material specific. We have not observed a clear correlation between the detailed charge transfer mechanism and antisite formation energy. If other electronic configurations are used in our DFT modeling, the details of the charge transfer process may change somewhat. However, the trend of the antisite formation energy should not change significantly because the difference in ionic radius between TM and alkali cations is the key factor for determining the antisite defect formation energy. Meanwhile, our density of states (DOS) calculations suggest that the introduction of antisite defects might give all these defective materials more metallic-like characteristics as their bandgaps disappear (Figures S28 and S29). However, such a prediction needs further experimental validation, which is beyond the scope of this work.

Evolution to a metallic like behavior is because of the disappearance of bandgap upon antisite defect formation as determined through our density of states calculation. We provided further explanation about the metallic behavior of the defective material in the Figure S28 and copied below for your reference.

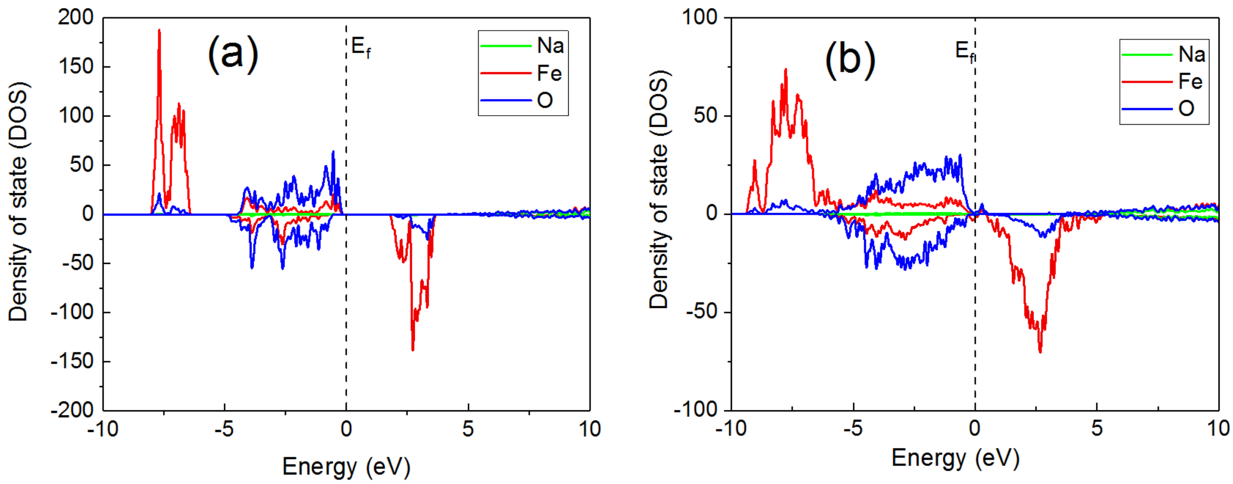

Figure S28. Density of states (DOS) for (a) perfect O3-$NaFeO_2$ and (b) defective O3-$NaFeO_2$ containing an antisite pair. To check how the formation of antisite defects modifies the electronic structures of the battery materials, density of states (DOS) are calculated. In both plots, the Fermi level is shifted to zero. In the perfect O3-$NaFeO_2$ (Figure a), a bandgap can be clearly seen between the valance band maximum (VBM) and conduction band minimum (CBM). The asymmetry in the up-spin and down-spin channels indicates that the material also has some magnetism. When the system contains a pair of antisite defects (Figure b), interestingly the VBM and CBM connect to each other and the bandgap disappears. Similar behavior also can be found in P2-$NaFeO_2$ and O3-$LiNiO_2$. The result suggests that the defective system might have a metallic characteristic. However, the insulating to metallic transition may be an outcome of the high antisite defect concentration in DFT calculations. In our simulation, the concentration of antisite defects is about 4% (1/24) with respect to the total cation sites in O3-$NaFeO_2$. It is unclear if such a high antisite concentration is achievable in the irradiation experiments or at the total fluence of 6.25×10^{14} Kr^{++}/cm^2 utilized to irradiate Na-layered cathode at room temperature. Thus, further experimental investigation is needed to validate this theoretical prediction in the future.

Our density of states calculation shows that the disappearance of bandgap and as a consequence of the development of metallic behavior is observed in all materials systems upon the introduction of antisite defects, irrespective of the specific charge transfer behavior. As shown in Figure S28 (copied above), the disappearance of the bandgap is observed in both defective O3-$NaFeO_2$ and defective P2-$NaFeO_2$. Additional calculation on the defective P2-$Na_{2/3}Fe_{1/2}Mn_{1/2}O_2$ also shows the disappearance of the bandgap upon antisite defect formation (see Figure S29 copied below for more details). Hence, the development of metallic characteristics is consistent in all four materials system and independent of the specific charge transfer behavior of the defective materials. We have revised our statement in the main text which is also copied below:

Page 29-30. Meanwhile, our density of states (DOS) calculations suggest that the introduction of antisite defects might give all these defective materials more metallic-like characteristics as their bandgaps disappear (Figures S28 and S29). However, such a prediction needs further experimental validation, which is beyond the scope of this work.

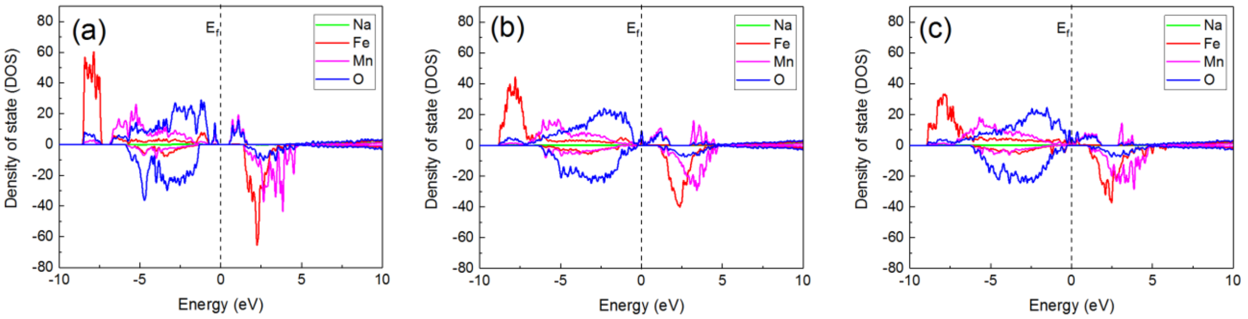

Figure S29. Density of states (DOS) for (a) a pristine P2-$Na_{2/3}Fe_{1/2}Mn_{1/2}O_2$, (b) a defective system containing an Fe-Na antisite pair, and (c) a defective system containing a Mn-Na antisite pair. Note that we did not use any hybrid functional to tune the bandgap width because the study of electronic properties is beyond the scope of this work. Similar to Figure S28, the bandgap vanishes in the two defective systems.

5. For explanation of better charge transfer and low formation energy in antisite defect, the simulation of NaFeO2 and LiNiO2 was applied. The reason regarded to ionic radius differences in case of Mn also seem not to be mentioned. The differences in electronic configuration may also give a significant change in charge transfer of antisite defect. Could the author give any comment on this doubting?

Response: To directly address the reviewer's comments, we have calculated the antisite formation energies for three Fe-Na and three Mn-Na antisite pairs in the complex P2-$Na_{2/3}Fe_{1/2}Mn_{1/2}O_2$ so that we can draw our conclusions without any ambiguity. For the reviewer's convenience, we have copied the atomic configuration of P2-$Na_{2/3}Fe_{1/2}Mn_{1/2}O_2$ and antisite pair positions, as shown below. Note for Mn^{3+} (0.58 Å, 0.645 Å), it has a nearly identical ionic radius as Fe^{3+} (0.55 Å, 0.645 Å), where the two values in "()" correspond to low spin and high spin states, respectively. For Mn^{4+} (0.585 Å) and Fe^{4+} (0.53 Å), which may present in P2-$Na_{2/3}Fe_{1/2}Mn_{1/2}O_2$, their ionic radius is also similar. Therefore, it is expected that a Mn-Na antisite pair should also have a high formation energy. Our independent DFT calculations indeed confirm this hypothesis and they are all much higher than that in $LiNiO_2$ (-0.54/0.23 eV in Table 1):

Fe1-Na1: 2.73 eV

Fe2-Na2: 3.22 eV

Fe3-Na2: 3.00 eV

Mn1-Na1: 4.04 eV

Mn2-Na1: 4.33 eV

Mn3-Na2: 5.05 eV.

We have updated our new results in Table 1. On page 24 - 25 of the main text, we have added the details about the DFT prediction of atomic configuration of P2-$Na_{2/3}Fe_{1/2}Mn_{1/2}O_2$, in particular

DFT predicts that two different Na sites (2d site and 2b site) exist, which is consistent with experiments [*Solid State Ionics* **161**, 31–39 (2003)]. We have added more discussion about the difference in ionic radii between TM and alkali, their correlation with antisite pair formation energy, and radiation tolerance. Table 1, Figure 6 and related texts explaining the results of the new DFT calculations are copied below for your reference:

Table 1. DFT results of the lattice parameters, bandgaps, and antisite formation energies in four model systems.

Materials	System size (atoms)	a (Å)	c (Å)	Bandgap (eV)	Antisite pair distance (Å)	Antisite pair formation energy (eV)
$LiNiO_2$ (O3)	96	2.88 (this work)<br>2.88 (Exp.)[71]	14.35 (this work)<br>14.19 (Exp.)[71]	2.07	12.0	-0.54 / 0.23*
$NaFeO_2$ (O3)	96	3.04 (this work)<br>3.03 (Exp.)[72]	16.09 (this work)<br>16.10 (Exp.)[72]	1.85	13.5	4.32
$NaFeO_2$ (P2)	64	3.03 (this work)<br>2.96 (DFT)[73]	10.81 (this work)<br>10.68 (DFT)[73]	1.84	8.8	4.52
$Na_{2/3}Fe_{1/2}Mn_{1/2}O_2$ (P2)	88	2.97 (this work)<br>2.93 (Exp.)[47]	11.15 (this work)<br>11.22 (Exp.)[47]	0.54	Fe_1-Na_1: 8.6<br>Fe_2-Na_2: 8.9<br>Fe_3-Na_2: 9.8<br>Mn_1-Na_1: 8.9<br>Mn_2-Na_1: 9.0<br>Mn_3-Na_2: 8.9	2.73<br>3.22<br>3.00<br>4.04<br>4.33<br>5.05

*The 0.23 eV is obtained using a 48-atom system.

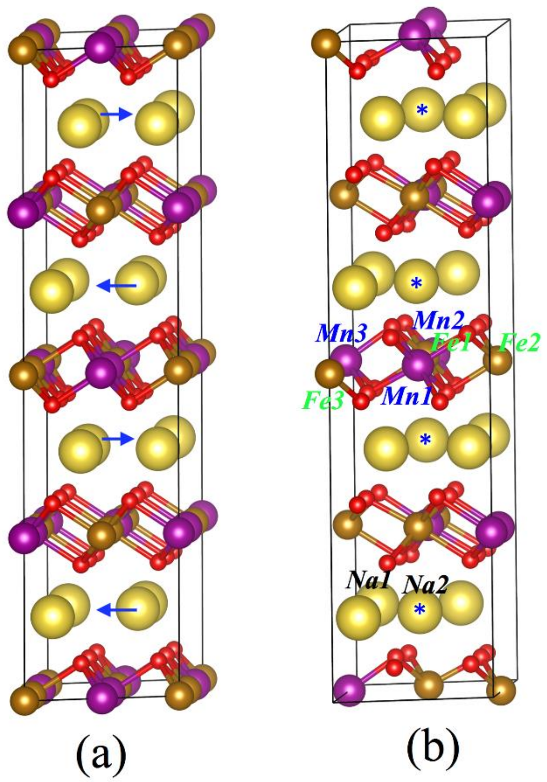

Figure 6. Atomic configurations of P2-$Na_{2/3}Fe_{1/2}Mn_{1/2}O_2$ and antisite defect pair positions. (a) Before structural relaxation. All Na cations are placed at 2d sites initially. The blue arrows indicate the moving directions of some Na cations after relaxation. (b) After structural relaxation. The Na cations with an asterisk (*) are those moving to the new 2b sites. The labeled TM and Na cations are those used to create antisite pairs. The two figures show some additional atoms at simulation box boundaries for visualization purpose (based on periodic boundary conditions). Large yellow spheres: Na; Medium brown spheres: Fe; Medium purple spheres: Mn; Small red spheres: O.

Page 24-25. As to Mn^{3+}, its ionic radius (0.58 Å, 0.645 Å) is nearly identical as Fe^{3+} for each spin state.[76] In P2-$Na_{2/3}Fe_xMn_{1-x}O_2$, Mn^{4+} and Fe^{4+} may also exist according to the X-ray absorption spectroscopy (XAS) measurements[77] and their ionic radii are also similar (0.585 Å vs 0.53 Å).[76] Therefore, if the ionic radius difference between alkali and TM cations is the key factor for affecting the antisite formation energy (and thus the radiation tolerance), a Mn-Na antisite pair should also have a high antisite formation energy. To prove this hypothesis, Mn-Na and Fe-Na antisite formation energies are directly calculated in P2-$Na_{2/3}Fe_{1/2}Mn_{1/2}O_2$. More complex than the ideal P2-$NaFeO_2$, the Na cations in $Na_{2/3}Fe_{1/2}Mn_{1/2}O_2$ do not have a full site occupancy and the TM layer consists of both Mn and Fe cations. Moreover, it has been shown experimentally that Na cations can stay in two different sites in $Na_{2/3}Fe_xMn_{1-x}O_2$: 2b (0, 0, 1/4) and 2d (2/3, 1/3, 1/4),[77] although it is unclear the exact arrangement of Na cations at the two sites. To predict the atomic configuration of P2-$Na_{2/3}Fe_{1/2}Mn_{1/2}O_2$, a P2-$NaFeO_2$ consisting of 3×2×2 unit cells (96 atoms in total) is created initially. In each of four TM layers, three out of six Fe cations are replaced by Mn

cations so that the Fe:Mn ratio is 1:1 in each TM layers (Figure 6a). All Na cations are initially placed at the 2d sites. Then two out of six Na cations in each of four Na layers are removed. Now the system has 88 atoms in total (16 Na, 12 Fe, 12 Mn, 48 O), which has the same stoichiometry as $Na_{2/3}Fe_{1/2}Mn_{1/2}O_2$. After structural relaxation, interestingly one Na cation in each of four Na layers moves from a 2d site to a 2b site. The moving directions of these Na cations are illustrated in Figure 6a and the final configuration is shown in Figure 6b. The final Na site occupancy factors are 0.5 for 2d site and 0.17 for 2b site in $Na_{2/3}Fe_{1/2}Mn_{1/2}O_2$, which are similar to 0.43 for 2d site and 0.26 for 2b site in $Na_{2/3}Fe_{1/3}Mn_{2/3}O_2$ as determined by experiments.[77] Therefore, our DFT calculation predicts reasonable Na site occupancy factors without any *a prior* assumptions. In addition, the predicted lattice parameters are also similar as the experimental values, as shown in Table 1.

The consistency of the newly calculated antisite formation energy in $Na_{2/3}Fe_{1/2}Mn_{1/2}O_2$ to our original claims further proves that the explanation of radiation resistance of Li- and Na-layered cathodes in terms of cationic antisite defect formation energy is justified. This further proves that the cationic size difference is the key parameter determining the radiation resistance of the layered cathode materials. This strengthens our conclusion on the design principle of stable layered cathode materials proposed in the study (copied below).

Page 31-32. The findings suggest that structural transformations in both Li- and Na-layered cathodes under irradiation follow the similar principle of cationic antisite defect formations, similar to pyrochlore oxides. Hence, our study provides a valuable guideline for designing stable layered cathodes under extreme conditions such as outer space exploration and nuclear power industries. Between different layered oxides (A_xTMO_2; where A is alkali ion, and TM is transition metal ion), a material with a smaller difference in the ionic size between A and TM will have a smaller cationic antisite defect formation energy and will be more resistant to radiation damage.

As for the effect of electronic configuration on charge transfer, we have addressed it together in the question 4. In short, we think it may affect the details of the charge transfer process, but the trend in the antisite defect formation energy should not change significantly as it is governed by the difference in the ionic radii between TM and alkali cations.

We have explained the issue in the main text and copied below for your reference:

Page 29. The above analysis shows that the detailed charge transfer/redistribution mechanism is material specific. We have not observed a clear correlation between the detailed charge transfer mechanism and antisite formation energy. If other electronic configurations are used in our DFT modeling, the details of the charge transfer process may change somewhat. However, the trend of the antisite formation energy should not change significantly because the difference in ionic radius between TM and alkali cations is the key factor for determining the antisite defect formation energy.

6. In the discussion on loss of crystallinity, while the full amorphization of Na2/3Fe1/2Mn1/2¬O2 is shown clearly, the similar result of LiNiO2 is not presented. And the area of amorphous layer in LiNiO2 at ion beam dose of 3.13x1014 Kr++/cm2 is larger than that of Na2/3Fe1/2Mn1/2¬O2 which is conflicted to the conclusion of the author. The further evident and explanation should be added.

Response: Thank you for the insightful comment. One of our main goals of this study was to figure out what factors govern the resistance to radiation damage of layered oxide cathodes so that design rules can be developed for stable cathode materials under irradiation. Our theoretical and experimental results present the fact that Li-layered cathode ($LiNiO_2$) is more resistant to radiation damage than Na-layered cathode ($Na_{2/3}Fe_{1/2}Mn_{1/2}O_2$) due to the smaller cationic antisite defect formation energy in the former (Figure 2 and Table 1 in the main text). In order to reach to this pivotal conclusion, observing the full amorphization of Li-layered cathode is not critical. Rather a comparison between the structural transformations between Na- and Li-layered cathode at the same irradiation fluence forms the basis of the conclusion. In fact, observing the earlier amorphization of Na-layered cathode did not necessitate figuring out the dose required for the full amorphization of Li-layered cathode. We still showed the structural transformations of Li-layered cathode beyond $6.25x10^{14}$ Kr^{++}/cm^2 (the dose required to amorphize the Na-layered cathode) in Figure S5 of the supplemental information which is also copied below.

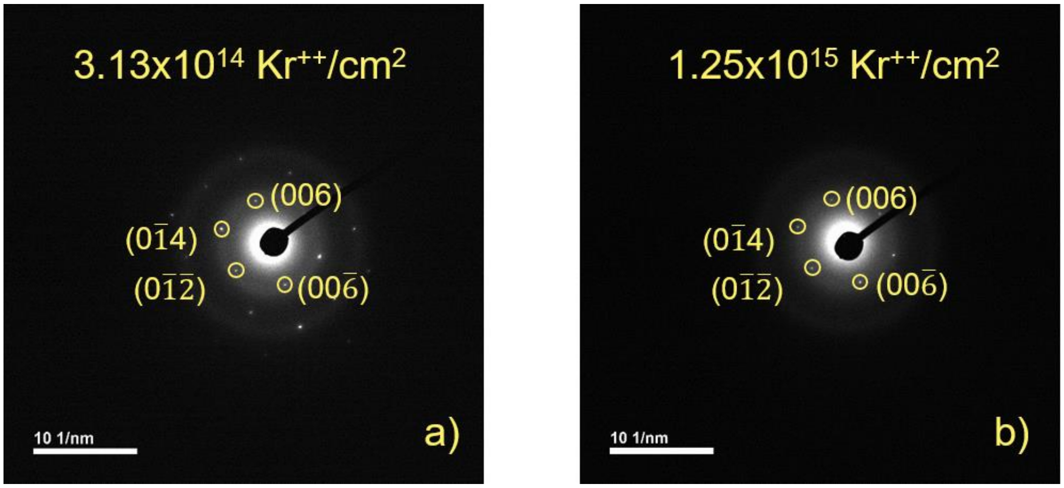

Figure S5. Electron diffraction pattern of $LiNiO_2$ at the fluence of (a) $3.13x10^{14}$ Kr^{++}/cm^2, and (b) $1.25x10^{15}$ Kr^{++}/cm^2.

In fact, at double the fluence of $6.25x10^{14}$ Kr^{++}/cm^2, complete amorphization of Li-layered cathode was still not observed (Figure S5b). This further strengthens our conclusion that Li-layered cathode is more resistant to radiation damage than Na-layered cathode.

While there was a continuous development of amorphous layers on both the materials, the nature of the two amorphous layers are fundamentally distinct. For $Na_{2/3}Fe_{1/2}Mn_{1/2}O_2$, the growth of the amorphous layer indicated the transformation of crystalline $Na_{2/3}Fe_{1/2}Mn_{1/2}O_2$ to amorphous $Na_{2/3}Fe_{1/2}Mn_{1/2}O_2$.

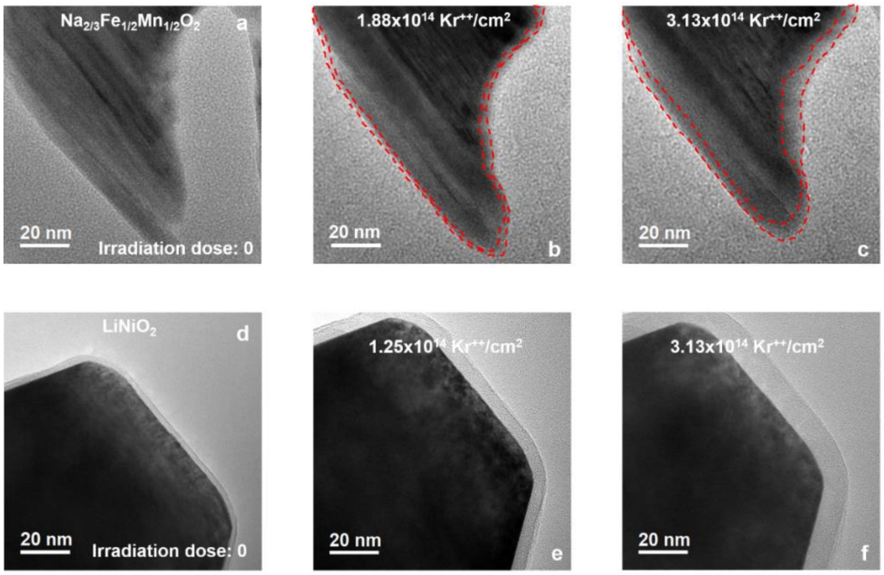

Figure 3. TEM images of $Na_{2/3}Fe_{1/2}Mn_{1/2}O_2$ and $LiNiO_2$ at various fluences of Kr ion irradiation at room temperature. (a) TEM image of $Na_{2/3}Fe_{1/2}Mn_{1/2}O_2$ before irradiation. TEM images of $Na_{2/3}Fe_{1/2}Mn_{1/2}O_2$ at the fluence of (b) $1.88x10^{14}$ Kr^{++}/cm^2, and (c) $3.13x10^{14}$ Kr^{++}/cm^2. (d) TEM image of $LiNiO_2$ before irradiation. TEM images of $LiNiO_2$ at the fluence of (e) $1.25x10^{14}$ Kr^{++}/cm^2, and (f) $3.13x10^{14}$ Kr^{++}/cm^2.

Based on the TEM images of $Na_{2/3}Fe_{1/2}Mn_{1/2}O_2$ (Figure 3a-3c), one can observe that the amorphous layer grew within the particle, which is supported by the electron diffraction results (Figure 2a-2e). Meanwhile, the amorphous layer on $LiNiO_2$ grew on the surface of the particle without any change of the crystallite shape (Figure 3d-3f). It is evident that the amorphous layer on the surface of the $LiNiO_2$ is a build-up of carbon contamination due to the entrapment of trace carbon by the electrons inside the TEM column. Such build-up of carbon deposit has been previously reported in the literature [*Br. J. Appl. Phys.* **4**, 101 (1953); *Br. J. Appl. Phys.* **5**, 27 (1954)]. The carbon contamination can also build up on $Na_{2/3}Fe_{1/2}Mn_{1/2}O_2$ but the distinction between the two different amorphous layers may become less discernible. We have added the following statement in the main text of the manuscript to clear up the confusion:

Page 11. It must be noted that the amorphous layers on these two materials are fundamentally distinct from each other. The growth of the amorphous layer within the particle of $Na_{2/3}Fe_{1/2}Mn_{1/2}O_2$ indicates a transformation from crystalline to amorphous phase, which is supported by the electron diffraction results (Figure 2a-2e). Meanwhile, the transparent amorphous

layer on the surface of $LiNiO_2$ indicates that the growth of this layer is due to the entrapment of trace carbon by electrons inside the TEM column.[54,55]

Reviewer #2 (Remarks to the Author):

The manuscript entitled "Defect and structural evolution under high energy ion irradiation informs battery materials design for extreme environments" reports in situ TEM characterization of defect evolution in high energy Kr ion irradiated Na- and Li-layered positive electrode materials (Na2/3Fe1/2Mn1/2O2 and O3-LiNiO2). Defect engineering is an emerging field in battery research. However, how defects influence the electrochemical and structural properties of battery materials is largely unknown because the characterization of defects or defect evolution is very challenging. New advanced characterization techniques or analyses are desired to explore the role of defects. The responses of electrode materials under ion irradiation can be used as an analog to understand the defect evolution within such materials upon electrochemical cycling in battery systems. In this work, the authors applied an unique and comprehensive mathematical analysis on bright field TEM images of the layered electrode materials under Kr+ irradiation at different fluences. The statistical analysis was done through pixel by pixel gradient vector (due to brightness change in image) computation. This is the first time such analysis is applied to understand defect evolution in ion irradiated battery materials. The analysis suggests the preferential alignment of defect clusters along the transport ion diffusion channels. The results are new and significant. They help elucidate defect evolution in electrode materials under ion irradiation. Through in situ TEM, it was also found that LiNiO2 is more resistant to radiation damage. Such phenomenon is explained through theoretical analysis (DFT), which revealed that antisite defect formation energy is smaller in LiNiO2 because of the small difference in ionic radius between Li+ and Ni3+, compared to P2-Na2/3Fe1/2Mn1/2O2 which have larger difference in ionic radius between Na+ and transition metals. **The results presented in this work will help to understand the defect evolution in solid state battery materials and therefore it is suggested for publication at Nat. Comm. with minor revision.**

Response: Thank you very much for identifying the novelty and the potential impact of our work and for your recommendation for the publication of the manuscript with minor revision.

(1) The direction of the Kr+ ion beam should be labelled in Figure 3 in order to better understand the propagation of the defects.

Response: Thank you for your suggestion. Ion irradiation is 30º away from the direction "into the plane of the paper". We have added the following statement in the "Materials and methods" section about the direction of the Kr ion irradiation:

Page 35. Electron irradiation for TEM imaging was in the direction "into the plane of the paper". Kr ion irradiation was incident at an angle of 30° with respect to the electron irradiation.

(2) The authors are suggested to include a short discussion till the end to relate the responses of

the layered electrode materials to ion irradiation and the corresponding defect evolution to their electrochemical performance such as cycling stability.

Response: Thank you for the suggestion. Evolution of the electrochemical performance of layered cathodes can be directly correlated to the defect evolution. Electrochemical cycling can induce the formation of point defects which can negatively impact the cycling performance. Interstitial type point defects are similar to transition metal migration observed during the electrochemical cycling of layered cathodes. Transition metal migration has been attributed to the voltage fading of Li-rich layered cathodes [*Nat. Mater*. **14**, 230–238 (2014)]. Voltage fading negatively impacts the energy efficiency of these materials which hinders their practical applications. A large quantity of antisite defects similar to transition metal migration are formed due to ion irradiation. The high density of antisite defects can modify the electrochemical performance of a layered cathode. As a proof of concept, we have performed ion irradiation on $Na_{2/3}Fe_{1/2}Mn_{1/2}O_2$ at the electrode level and conducted electrochemical performance evaluation. In the figure below, we have shown the CV profiles of pristine and ion irradiated $Na_{2/3}Fe_{1/2}Mn_{1/2}O_2$ (Figure R3). The appearance of an extra feature on the positive voltage swipe (peak marked by "*" in Figure R3b) indicates that the redox evolution of the irradiated electrode has been modified on ion irradiation. Detailed study is currently underway to mechanistically understand the evolution of electrochemical performance on irradiated cathodes and will be reported elsewhere.

Figure R3. Cyclic voltammetry curves of (a) pristine, and (b) Ne ion irradiated ($2.42x10^{14}$ Ne ion/cm^2) $Na_{2/3}Fe_{1/2}Mn_{1/2}O_2$ electrodes. The scan rate was 0.15 mV/s.

A large amount of antisite defect formation due to transition metal migration coupled with oxygen evolution can lead to phase transformation of layered cathodes to spinel or rocksalt structure [*Nat. Commun.* **5**, 3529 (2014)]. Such phase transformation leads to accelerated transition metal dissolution, cathode particle cracking, and impedance development [*ACS Appl. Mater. Interfaces* **11**, 37885–37891 (2019)]. Extensive material damage due to phase transformation and oxygen evolution may promote amorphization of layered cathodes, causing accelerated electrochemical performance degradation [*ACS Energy Lett.* **4**, 2409–2417 (2019)]. According to your suggestion, we have added the following discussion on the relationship between defect evolution and electrochemical performance of layered electrode materials.

Page 33. Point defects such as vacancies and interstitials can largely influence the electrochemical performance of layered cathodes. Interstitials resulting from the transition metal migration are reported to cause voltage decay in high energy Li-rich layered cathode materials.[43] Voltage decay results in subpar energy efficiency, which hinders the commercialization of these promising cathode materials. Large quantity of interstitial defects can cause phase transformation from layered to spinel or rocksalt phase,[42] leading to transition metal dissolution, cathode particle cracking, and high electrochemical impedance development.[86] Extensive material damage due to phase transformation and oxygen evolution may induce amorphization, leading to accelerated electrochemical performance degradation.[87] The aforementioned structural and chemical stability issues can be alleviated to some degree through doping chemistry.[48] Radiation creates a high

concentration of point defects. The impacts of irradiation-induced defects on the electrochemical performance of Li- and Na- layered cathodes and whether doping can play a role in the stability under irradiation deserve further studies in the future.

(3) The authors suggested "the possible formation of interstitial-type defect clusters…" what about the possibility of cation vacancies?

Response: Thanks to the reviewer for addressing the important topic of cationic vacancy. Radiation creates interstitials and vacancies in equal number (called "Frenkel pairs"). Interstitials can accumulate to form interstitial clusters or dislocation loops. Vacancies can accumulate to form voids. To form these extended defects, defect mobility is critical. Typically, interstitials diffuse must faster than vacancies in materials. Therefore, interstitial-type defect clusters or dislocation loops are typically observed first. Vacancy diffusion typically requires a high temperature. In this work, we have not observed any void formation, indicating that vacancy diffusion is not active at the temperatures studied here. We explained these aspects in the manuscript which is also copied below:

Page 4. Under irradiation, high-energy particles such as neutron or Kr ions can displace atoms away from their lattice sites and form a locally disordered region, called cascade.[36–38] A cascade can recover in a few picoseconds (10^{-12} sec) but some displaced atoms can form defects such as interstitials and vacancies. The aggregation of these point defects can form extended defects such as dislocation loops and voids.[39] Dislocation loop and void formation will require the diffusion of interstitials and vacancies at the temperature of irradiation, respectively.

In addition to cationic vacancies, anionic vacancies in the form of oxygen evolution can also take place in an ion irradiated material. Our experimental analysis shows that at high temperature, $Na_{2/3}Fe_{1/2}Mn_{1/2}O_2$ transforms into a spinel phase instead of a direct transformation to an amorphous phase. Such transformation to a cation dense spinel phase would require oxygen evolution as well as cation migration. We have added the following statement in the manuscript to highlight this aspect.

Page 12. Formation of the spinel phase may indicate oxygen evolution in order to form a cation densified state, according to previously reported literature.[57]

(4) The authors have done significant work in the past to study the effects of doping. Doping in electrode materials are known to improve structural stability. It would be interesting to know how doping will affect the responses of the layered materials under ion irradiation.

Response: Thanks to the reviewer for referring to this interesting point. From our previous studies, we have indeed found that the structural and electrochemical stability of a layered oxide cathode can be improved through doping chemistry. For example, the surface to bulk structural and chemical stability of $LiNiO_2$ can be improved through doping with Mg/Ti [*Chem. Mater.* **31**, 9769–9776 (2019)] and Mg/Mn [*ACS Appl. Mater. Interfaces* **12**, 12874–12882 (2020)] dual dopants.

Our work has also shown that manipulating the three-dimensional distribution of Ti dopant on Ni rich layered cathodes [*ACS Appl. Mater. Interfaces* **11**, 37885–37891 (2019)] can improve the oxygen stability. Improved oxygen stability can enhance the stability of layered materials against structural transformation to spinel or rocksalt phase during electrochemical cycling. Oxygen stability imparted by doping chemistry can similarly enhance the resistance to structural transformations during irradiation. Our previous studies have also shown that the density of the antisite defects can be manipulated through doping [*Chem. Mater.* **31**, 9769–9776 (2019); *ACS Appl. Mater. Interfaces* **12**, 12874–12882 (2020)]. Through manipulating the distribution of the dopants in the specific lattice site of the crystal structure, the concentration of the antisite defects in $LiNiO_2$-based materials can be tuned (Figure R4). Such control can provide an avenue to potentially control the radiation resistance of a layered oxide material through doping.

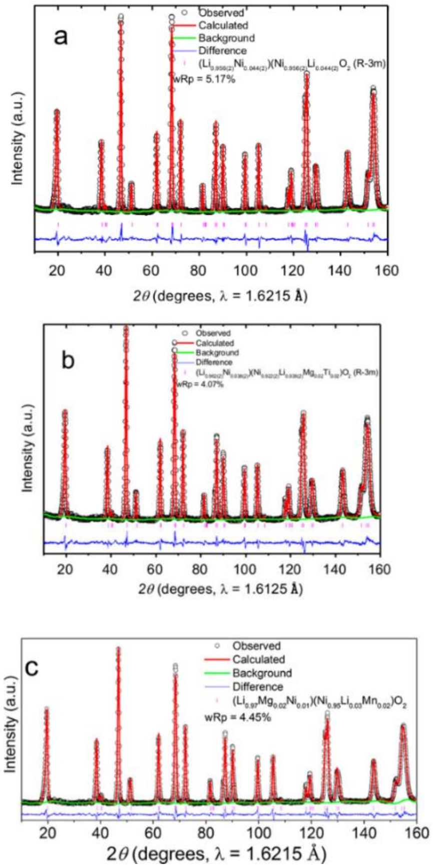

Figure R4. Neutron diffraction pattern with Rietveld refinement of (a) $LiNiO_2$ (Ni antisite defect was 4.4%), (b) Mg/Ti dual doped $LiNiO_2$ (Ni antisite defect was 3.8%), and (c) Mg/Mn dual doped $LiNiO_2$ (Ni antisite defect was 1%). Adapted with permission from references 2 and 3.

Our future studies relating to the stability of cathode materials in extreme environments will explore the effect of doping chemistry. However, in order to form a launching platform for deeper understanding, ion irradiation study on simple layered cathode materials as the first step can enable us to build a baseline to mechanistically explore the degradation of layered cathodes under extreme environments. We have added the following statement in light of the comment:

Page 33. The aforementioned structural and chemical stability issues can be alleviated to some degree through doping chemistry.[48] Radiation creates a high concentration of point defects. The impacts of irradiation-induced defects on the electrochemical performance of Li- and Na- layered cathodes and whether doping can play a role in the stability under irradiation deserve further studies in the future.

Reviewer #3 (Remarks to the Author):

This manuscript reports on defect evolution in layered electrode materials under high energy ion irradiation. Overall, **the study is of high quality**, although I'm not entirely convinced that a publication in nature communication is warranted. My objections are:

Response: Thank you for your valuable comments. We have responded to your comments in detail and performed necessary changes in our manuscript. We hope our elaborate response to your concerns and carefully revised manuscript will convince you to accept this work for publications in Nature Communications.

1. The connection to the electrochemical performance is vague. No doubt that many defects are formed during electrochemical cycling, however, the nature of the defects could be very different from that created by high energy ion radiation. How does the current study improve our understanding on defect dynamics associated with electrochemical cycling? how does the charge/discharge profile change after irradiation? The authors did mention that "the dynamics of defect evolution and structural transformations through electrochemical cycling shares similarities to that under the high energy ion irradiation". Unfortunately, no further explanation was given, and no reference was provided.

Response: Thank you for your comment. High energy ion irradiation can produce large quantity of vacancies and interstitials. Interstitial defects involving transition metal ions are produced when they migrate to the alkali-ion site (Li/Na site). Similar defect formation through transition metal migration to the alkali-ion site is widely observed during electrochemical cycling [*Nat. Mater*. **14**, 230–238 (2014)]. We acknowledge the fact that many different types of defects can form during electrochemical cycling. However, our study particularly provides insights about interstitial type defect which is mechanistically similar to transition metal migration during electrochemical cycling. Transition metal migration may lead to structural transformation and electrochemical performance degradation of layered cathodes [*Nat. Mater*. **14**, 230–238 (2014); *Nat. Commun.* **5**, 3529 (2014)]. Voltage fading is a critical issue in many high energy layered cathodes which is a direct consequence of transition metal migration [*Nat. Mater*. **14**, 230–238 (2014)]. Transition metal migration can be visualized through high resolution TEM imaging but the small field of view of the HRTEM images cannot represent the full picture of the whole particle [*Nano Lett.* **13**, 3857–3863 (2013); *Nat. Commun.* **6**, 1–9 (2015)]. Meanwhile, present state-of-the-art defect imaging such as Bragg coherent diffraction imaging can monitor dislocation lines on cathode materials at a much larger scale [*Science* **348**, 1344–1347 (2015)]. However, the limited resolution of the technique means that defects can only be imaged when they are large enough to form dislocation networks. Our quantitative mathematical analysis on the bright field images has enabled monitoring the defect evolution in the whole particle which is not necessarily limited to the formation of large dislocation networks. Hence, for the first time, we have shown that defect

clusters of vacancies and interstitials have a preferred direction of evolution (along the ion-diffusion channels) which can enrich our present understanding of defect evolution in cathode materials. Since interstitial defects are mechanistically similar to transition metal migration, our study can provide insight about defect evolution in layered cathodes during electrochemical cycling. However, we should emphasize that the focus of our work was to understand the irradiation damage of layered cathode materials so that design principles can be developed for stable cathode materials under extreme environments. Our results show that the structural transformations in both Li- and Na-layered cathodes under irradiation follows the similar principle of cationic antisite defect formations. Hence, our study provides a valuable guideline for designing stable layered cathodes under extreme conditions such as outer space exploration and nuclear power industries. Between different layered oxides (A_xTMO_2; where A is alkali ion, and TM is transition metal ion), a material with a smaller difference in the ionic size between A and TM will have a smaller cationic antisite defect formation energy and will be more resistant to radiation damage. Hence, the understanding of defect evolution of cathode materials is an added merit of our work. We have made substantial changes in our manuscript to clarify the issue and copied below for your reference:

Page 3-4. Defect and structural evolution can be accelerated in complex oxides through high energy ion irradiation.[25,26] Ion irradiation in conjunction with TEM have been utilized to understand the irradiation damage in nuclear reactor materials and fuels.[27–30] Alkali-ion batteries have the potential to be utilized in extreme environments such as outer space and nuclear power industries, where high energy irradiation can impart significant damage to materials.[31,32] Accelerated degradation of cell components such as cathode and electrolyte has been observed under neutron and gamma irradiation.[31,33] Radiation induced hardness is observed in perovskite tandem solar cells.[34] Structural transformation e.g. amorphization can take place in a crystalline material under extreme irradiation.[35] For reliable performance of battery materials in extreme environments, these materials are required to be resistant to such structural damage. Under irradiation, high-energy particles such as neutron or Kr ions can displace atoms away from their lattice sites and form a locally disordered region, called cascade.[36–38] A cascade can recover in a few picoseconds (10^{-12} sec) but some displaced atoms can form defects such as interstitials and vacancies. The aggregation of these point defects can form extended defects such as dislocation loops and voids.[39] Dislocation loop and void formation will require the diffusion of interstitials and vacancies at the temperature of irradiation, respectively. In comparison, interstitial type defects are also formed during electrochemical cycling through transition metal migration in the interlayer space.[40,41] Such migration can lead to structural transformation[42] and voltage fading.[43,44] Vacancy cluster formation in $Na_{0.75}Li_{0.25}Mn_{0.75}O_2$ is reported in as early as the first cycle.[15] Since vacancies and interstitials are also formed under ion irradiation, the material damage due to ion irradiation shares some similarities with the electrochemical cycling. Furthermore, the ability to create high-density defects in a short time through ion irradiation enables studying defect and structural evolution *in situ*,[45] thus overcoming the limitation of slow defect evolution through electrochemical cycling.

Figure R5. Electrochemical performance of Ne ion irradiated $Na_{2/3}Fe_{1/2}Mn_{1/2}O_2$. Galvanostatic charge-discharge curves of the cell containing $Na_{2/3}Fe_{1/2}Mn_{1/2}O_2$ irradiated at a total

fluence of (a) 2.42x10^{14} Ne^{++}/cm^2, and (b) 7.25x10^{14} Ne^{++}/cm^2 at C/5 rate for up to 20 cycles. (c) % capacity retention of $Na_{2/3}Fe_{1/2}Mn_{1/2}O_2$ irradiated at a total fluence of 2.42x10^{14} Kr^{++}/cm^2 and 7.25x10^{14} Kr^{++}/cm^2. (d) Average voltage vs cycle number of $Na_{2/3}Fe_{1/2}Mn_{1/2}O_2$ irradiated at a total fluence of 2.42x10^{14} Kr^{++}/cm^2 and 7.25x10^{14} Kr^{++}/cm^2. (e) Cyclic voltammetry curves of pristine $Na_{2/3}Fe_{1/2}Mn_{1/2}O_2$ at a scan rate of 0.15 mV/s. (f) Cyclic voltammetry curves of Ne ion irradiated (2.42x10^{14} Ne ion/cm^2) $Na_{2/3}Fe_{1/2}Mn_{1/2}O_2$ at a scan rate of 0.15 mV/s.

Nevertheless, we do acknowledge the fact that the electrochemical performance can be influenced by the accelerated defect evolution in the cathode material under ion irradiation. In the present work, our samples for in-situ irradiation must be electron transparent. Therefore, ion irradiation and TEM characterization were performed on primary particle of few hundred nanometers thickness. Recently, we have used Ne ions to conduct ex-situ irradiation on bulk (thicker) electrode materials. We will report the work elsewhere in the future. We chose not to include the preliminary data in the present work because 1) the irradiation conditions are different, and 2) the focus on the present work is to understand the defect and structural evolution of layered cathode materials in general under irradiation rather than the electrochemical performance.

In this response letter, for the reviewing purpose we would like to share some preliminary data of our ongoing ex-situ irradiation work. The ex-situ irradiation was performed with high energy Ne ion (400 KeV) at the total fluence of 2.42x10^{14} Ne^{++}/cm^2 and 7.25x10^{14} Ne^{++}/cm^2 on Na-layered cathode ($Na_{2/3}Fe_{1/2}Mn_{1/2}O_2$). From Figure R5a-d, it can be seen that the cycling stability of the irradiated $Na_{2/3}Fe_{1/2}Mn_{1/2}O_2$ is compromised with an increase in irradiation. The capacity retention was 77.5% after 20 cycles at C/5 rate for the electrode irradiated under the total fluence of 2.42x10^{14} Ne^{++}/cm^2 (Figure R5c). The capacity retention goes down to 70.1% after 20 cycles at C/5 rate for the electrode irradiated under the fluence of 7.25x10^{14} Ne^{++}/cm^2 (Figure R5c). Voltage fading is comparatively faster at higher irradiation dose (Figure R5d). In comparison, the pristine electrode has 86% capacity retention, even after cycling at a slower rate of C/10 for 20 cycles (see Figure S1a copied below).

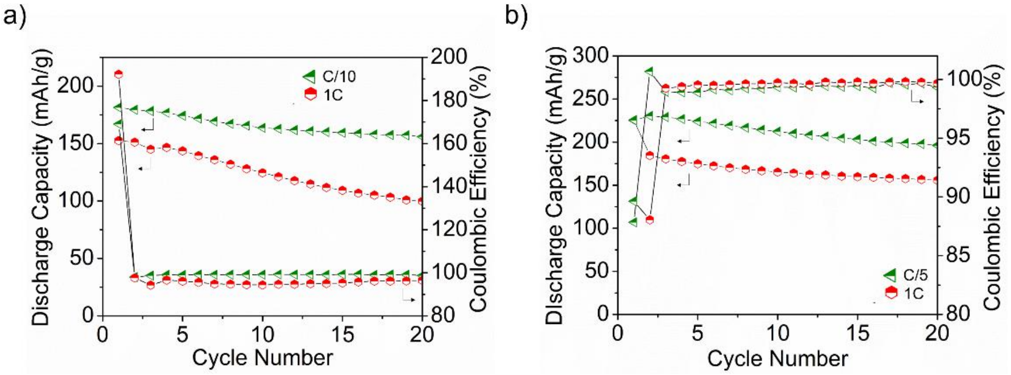

Figure S1. Capacity retention and Coulombic efficiency of (a) $Na_{2/3}Fe_{1/2}Mn_{1/2}O_2$, and (b) $LiNiO_2$ in Na half cell and Li half cell, respectively.

The CV curves in Figure R5e-f show the evolution of new redox feature on the positive voltage swipe in irradiated $Na_{2/3}Fe_{1/2}Mn_{1/2}O_2$ (marked by "*" in Figure R5f). This signifies a modification

of the redox response of the cathode materials after irradiation. Overall it is evident that the electrochemical performance is influenced by irradiation. Thus, irradiation damage in extreme environments will play a key role in overall degradation of the cathode materials during electrochemical cycling. However, figuring out the relationship between the irradiation damage and electrochemical performance will require a careful spectroscopic and imaging study so that the physicochemical aspects of cathode materials that are influencing the electrochemical performance can be determined. We should mention that we resonate with the concern of the reviewer and are performing additional experiments to establish a concrete relationship between the electrochemical performance and the ion irradiation which will be published elsewhere. We should also mention that the key focus of the present study is to understand defect dynamics and structural evolution of layered cathodes in general under ion irradiation so that design principles can be developed to synthesize stable layered cathodes under irradiation. The conclusion of this study states the successful attainment of our key goal of this particular study and copied below for your reference.

Page 31-32. The findings suggest that structural transformations in both Li- and Na-layered cathodes under irradiation follow the similar principle of cationic antisite defect formations, similar to pyrochlore oxides. Hence, our study provides a valuable guideline for designing stable layered cathodes under extreme conditions such as outer space exploration and nuclear power industries. Between different layered oxides (A_xTMO_2; where A is alkali ion, and TM is transition metal ion), a material with a smaller difference in the ionic size between A and TM will have a smaller cationic antisite defect formation energy and will be more resistant to radiation damage.

The relevance of the connection between electrochemical performance and interstitial defect formation is evident from the recent literature as well. Electrochemical cycling can induce the formation of point defects which can negatively impact the cycling performance. Interstitial type point defects are similar to transition metal migration observed during the electrochemical cycling of layered cathode materials. Transition metal migration has been attributed to the voltage fading of Li-rich layered cathodes [*Nat. Mater.* **14**, 230–238 (2014)]. Voltage fading negatively impacts the energy efficiency of these materials which is hindering their practical applications. A large quantity of interstitial defects similar to transition metal migration are formed due to ion irradiation. The high density of interstitial defects can modify the electrochemical performance of a layered cathode. Extensive amount of interstitial defect formation due to transition metal migration as well as oxygen evolution can lead to phase transformation of layered cathodes to spinel or rocksalt phase [*Nat. Commun.* **5**, 3529 (2014)]. Such phase transformation leads to accelerated transition metal dissolution, cathode particle cracking, and impedance development [*ACS Appl. Mater. Interfaces* **11**, 37885–37891 (2019)]. Extensive material damage due to phase transformation and oxygen evolution may promote amorphization of layered cathodes, causing accelerated electrochemical performance degradation [*ACS Energy Lett.* **4**, 2409–2417 (2019)]. We have highlighted this aspect in the “Conclusions and discussion” section of the manuscript:

Page 33. Point defects such as vacancies and interstitials can largely influence the electrochemical performance of layered cathodes. Interstitials resulting from the transition metal migration are

reported to cause voltage decay in high energy Li-rich layered cathode materials.[43] Voltage decay results in subpar energy efficiency, which hinders the commercialization of these promising cathode materials. Large quantity of interstitial defects can cause phase transformation from layered to spinel or rocksalt phase,[42] leading to transition metal dissolution, cathode particle cracking, and high electrochemical impedance development.[86] Extensive material damage due to phase transformation and oxygen evolution may induce amorphization, leading to accelerated electrochemical performance degradation.[87] The aforementioned structural and chemical stability issues can be alleviated to some degree through doping chemistry.[48] Radiation creates a high concentration of point defects. The impacts of irradiation-induced defects on the electrochemical performance of Li- and Na- layered cathodes and whether doping can play a role in the stability under irradiation deserve further studies in the future.

2. the authors argued that the current study informs battery design under extreme conditions. However, are battery electrodes subject to high-energy ion bombardment directly? If so, are the energy and fluence of Kr irradiation studied in this manuscript representative of the real service environment?

Response: Alkali-ion batteries have the potential for application in extreme conditions such as outer space and nuclear power industries. In outer space, alkali-ion batteries power electronic devices, satellites, probes, planetary rovers, and for load leveling applications [*Nucl. Instruments Methods Phys. Res. Sect. B Beam Interact. with Mater. Atoms* **345**, 27–32 (2015); *J. Power Sources* **318**, 242–250 (2016)]. Alkali-ion batteries are also used to power robotics for sampling or rescue efforts in incidents involving nuclear accidents, detonations, or in radioactive hot cells [*Nucl. Instruments Methods Phys. Res. Sect. B Beam Interact. with Mater. Atoms* **345**, 27–32 (2015)]. Failure of robots deployed for sampling and rescue mission due to intense irradiation is well documented in the 2011 Fukushima Daiichi nuclear disaster incident [*Ind. Rob.* **39**, 428–435 (2012)]. For application in outer space or in nuclear power industries, the batteries are required to be robust and capable of working in extreme temperature and irradiation. For example, batteries for outer space exploration are required to work in temperature ranging from -120 °C (in Mars) to 475 °C (in Venus) [*IEEE Aerospace Conference Proceedings* (2007). doi:10.1109/AERO.2007.352728]. These batteries are also expected to be tolerant to extreme irradiation (e.g. around 4 MRad in Jupiter). Materials in outer space are constantly exposed to high energy particles ejected during solar flares, coronal mass ejections, and galactic cosmic rays (https://www.nasa.gov/analogs/nsrl/why-space-radiation-matters). The energy of the outer space radiation may vary from KeV to GeV. Meanwhile, materials can be damaged due to high energy neutron irradiation of around 14 MeV in nuclear power plants [*J. Nucl. Mater.* **174**, 196–209 (1990)]. Prolonged exposure to these radiations will compromise the structural integrity of material and the performance of functional materials are expected to degrade over time [*Electrochim. Acta* **51**, 6320–6324 (2006)]. Hence, understanding the irradiation damage is a crucial step towards developing safe and reliable energy storage devices for applications in extreme environments. There are several studies that show that battery materials when exposed to neutron or gamma irradiation undergoes structural and chemical evolution that degrade their electrochemical performance. For example, He et al. showed that the cathode particle size and roughness increased on exposure to neutron and gamma irradiation [*Nucl. Instruments Methods Phys. Res. Sect. B Beam Interact. with Mater. Atoms* **345**, 27–32 (2015)]. Structural disordering is also increased on exposure to irradiation. Accelerated electrolyte decomposition is another observed issue [*J. Power*

Sources **318**, 242–250 (2016)]. Similar to alkali metal-ion batteries, perovskite solar cells are also degraded on exposure to irradiation such as proton irradiation [*Joule* **4**, 1054–1069 (2020)]. Hence, the degradation of battery materials is a real issue on environments with extreme irradiation. Developing stable materials under extreme environment will require mechanistically understanding the transformation of battery materials when exposed to irradiation. Temperature is another key parameter that can aggravate or alleviate the irradiation damage of a material. Our study, for the first time, provides valuable principle for the design of stable battery materials in extreme environment by fundamentally understanding defect evolution and structural transformation at a broad range of temperature under high energy irradiation. Hence, we believe, the findings of this study will benefit the design of stable battery materials in extreme environments. The manuscript is edited accordingly to highlight the practical importance of irradiation resistance of batteries and copied below for your reference:

Page 3-4. Defect and structural evolution can be accelerated in complex oxides through high energy ion irradiation.[25,26] Ion irradiation in conjunction with TEM have been utilized to understand the irradiation damage in nuclear reactor materials and fuels.[27–30] Alkali-ion batteries have the potential to be utilized in extreme environments such as outer space and nuclear power industries, where high energy irradiation can impart significant damage to materials.[31,32] Accelerated degradation of cell components such as cathode and electrolyte has been observed under neutron and gamma irradiation.[31,33] Radiation induced hardness is observed in perovskite tandem solar cells.[34] Structural transformation e.g. amorphization can take place in a crystalline material under extreme irradiation.[35] For reliable performance of battery materials in extreme environments, these materials are required to be resistant to such structural damage.

Kr ion irradiation was chosen for this study to understand the microstructural evolution of battery materials under high energy irradiation environment. Typically, the utilized flux and fluence of Kr ion irradiation are higher than the normal service environment in nuclear reactors or in outer space. However, accelerated ion irradiation helps to impart observable materials damage within a short period of time. This enables studying the transformation of materials within a few hours which otherwise would have taken years in actual service environment [*J. Nucl. Mater.* **37**, 1–12 (1970); *J. Nucl. Mater.* **216**, 78–96 (1994); *Scr. Mater.* **88**, 33–36 (2014)]. Thus, *in situ* study of the impact of irradiation on a material is possible. In addition, the neutron-irradiated materials are radioactive and require special facilities to handle them (there are very limited facilities in the world that can handle neutron-irradiated materials). Therefore, using ion irradiation such as Kr irradiation to emulate neutron damage is a fast, safe, and economic way as it captures the key defect and structural evolution behavior under neutron irradiation [*Journal of Materials Research* **30**, 1158–1182 (2015)]. Besides, the cascade damage profile produced by Kr ion irradiation is similar to neutron irradiation in nuclear reactor [*Journal of Applied Physics* **107**, 071301 (2010)], thus efficiently epitomizing the transformation of battery materials throughout the actual service life. We have added the explanation of this aspect in the manuscript and copied below for your reference:

Page 5. Kr ion irradiation can induce observable damage within a short period of time.[46] The cascade damage profile produced by Kr ion irradiation is similar to neutron irradiation in a nuclear reactor.[45] Hence, efficient mirroring of defect and structural evolution throughout the actual service life in extreme environments is possible within the timescale of laboratory experiment.

3. The authors reported total fluence, but I couldn't find any information regarding fluence rate, which is also an important parameter. Would different fluence rate make a difference?

Response: According to the reviewer's suggestion, we have included the information of the fluence rate of Kr ion irradiation in the "Materials and methods" section. The sentence is copied here for the reviewer's reference.

Page 35. The fluence rate of Kr ion irradiation was 6.25×10^{10} $Kr^{++}/cm^2/s$.

We agree with the reviewer that the fluence rate is an important parameter. However, in order to make a fair comparison between the two materials, we had to keep the fluence rate constant. The focus of our study was not study the effect of fluence rate but to study the transformations of the materials under similar conditions (e.g. fluence rate, irradiation energy, and temperature). Therefore, we believe ion irradiation at a defined set of conditions have made the conclusions in this study fair and allowed us to identify the stable layered cathodes under extreme irradiation conditions.

4. The authors reported a negative antisite energy for LiNiO2 system. Does that make sense?

Response: Yes, the result makes sense and is consistent with independent experimental observations. As discussed below, many experiments show that a high concentration of Li-Ni antisite defects exist in $LiNiO_2$ even without irradiation, indicating antisite defects can form spontaneously in $LiNiO_2$ (which requires a negative antisite defect formation energy). Our calculation shows that for one antisite defect pair, the formation energy is negative (-0.54 eV) in the 96-atom system in which the antisite defect concentration is 4.2%. The formation energy becomes positive at a higher concentration of antisite defect of 8.3% in the 48-atom system, indicating antisite defect concentration in $LiNiO_2$ can be a few percent. In experiments, it has been shown that it is difficult to obtain antisite-free $LiNiO_2$ [*J. Mater. Chem. A* **2**, 7988–7996 (2014)], even without irradiation. In fact, in many $LiNiO_2$ reported in the literature, a few percent of total Ni can be found sitting in the Li site, even in the pristine state [*Chem. Mater.* **31**, 9769–9776 (2019); *Angew. Chemie Int. Ed.* **58**, 10434–10458 (2019)]. Hence, our calculation is in accordance with the experimental observations reported in the literature for $LiNiO_2$. We have added the following sentence in the main text to explain the issue:

Page 23. Note that the negative antisite formation energy (-0.54 eV in 96-atom system) indicates that a perfect $LiNiO_2$ is difficult to obtain due to the spontaneous formation of Li-Ni antisite defects, even in the pristine state. In fact, a few percent of Ni sitting in the Li site is widely reported in the literature.[48,74] In some other $LiNiO_2$ based materials, the antisite concentration can be as high as 11.8% (Table 2 in Ref. 75). Therefore, our DFT results are consistent with these experimental observations.

Previous experimental studies on $LiNiO_2$ and multi-element doped $LiNiO_2$ performed in our group [*Chem. Mater.* **31**, 9769–9776 (2019); *ACS Appl. Mater. Interfaces* **12**, 12874–12882 (2020)] showed that around 1-4% Ni can be found on the Li site in the pristine state (Figure R6). A small amount of antisite defects in the pristine state indicates a low concentration of antisite defect formation in $LiNiO_2$ is spontaneous which is consistent with our computational result.

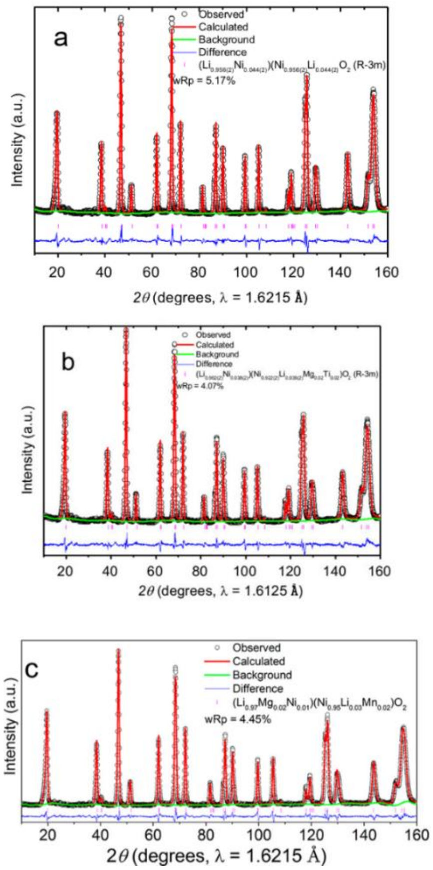

Figure R6. Neutron diffraction pattern with Rietveld refinement of (a) $LiNiO_2$ (Ni antisite defect was 4.4%), (b) Mg/Ti dual doped $LiNiO_2$ (Ni antisite defect was 3.8%), and (c) Mg/Mn dual doped $LiNiO_2$ (Ni antisite defect was 1%). Adapted with permission from references 2 and 3.

Besides, some other studies also report a negative antisite defect formation energy in some complex oxides such as pyrochlores, indicating that spontaneous antisite defect formation is possible in some oxides [*Science* **289**, 748–751 (2000)].

Reference

1. Mu, L. *et al.* Deciphering the Cathode-Electrolyte Interfacial Chemistry in Sodium Layered Cathode Materials. *Adv. Energy Mater.* **8**, 1801975 (2018).
2. Mu, L. *et al.* Dopant Distribution in Co-Free High-Energy Layered Cathode Materials. *Chem. Mater.* **31**, 9769–9776 (2019).
3. Mu, L. *et al.* Structural and Electrochemical Impacts of Mg/Mn Dual Dopants on the LiNiO2 Cathode in Li-Metal Batteries. *ACS Appl. Mater. Interfaces* **12**, 12874–12882 (2020).

REVIEWERS' COMMENTS:

Reviewer #1 (Remarks to the Author):
After carefully considering the response letter from the authors, all of the mentioned issues have been addressed. Therefore, I highly recommend that the manuscript titled as “Defect and structural evolution under high-energy ion irradiation informs battery materials design for extreme environments” of Muhammad M. R. et al. should be accepted for publication on Nature Communication journal.

Reviewer #2 (Remarks to the Author):
The authors have addressed the reviewer's comments and the updated manuscript is enhanced by addressing all feedbacks by the reviewers. Therefore, I suggestion its publication at Nature Communications.

Reviewer #3 (Remarks to the Author):
the authors did a good job responding to my questions (as well as concerns from other reviewers). I have read it through and do not have other questions. Overall, I think it is a nice manuscript and should be published in Nat. commun.

Reviewer #1 (Remarks to the Author):

After carefully considering the response letter from the authors, all of the mentioned issues have been addressed. Therefore, I highly recommend that the manuscript titled as “Defect and structural evolution under high-energy ion irradiation informs battery materials design for extreme environments” of Muhammad M. R. et al. should be accepted for publication on Nature Communication journal.

Response: We really appreciate the reviewer’s recommendation to publish the manuscript in Nature Communications.

Reviewer #2 (Remarks to the Author):

The authors have addressed the reviewer's comments and the updated manuscript is enhanced by addressing all feedbacks by the reviewers. Therefore, I suggestion its publication at Nature Communications.

Response: We thank the reviewer for the recommendation of publication of this manuscript.

Reviewer #3 (Remarks to the Author):

the authors did a good job responding to my questions (as well as concerns from other reviewers). I have read it through and do not have other questions. Overall, I think it is a nice manuscript and should be published in Nat. commun.

Response: We would like to extend our gratitude to the reviewer for the recommendation of publication of this manuscript in Nature Communications.
